# Sex-Specific Cardiovascular Protection in Developing Metabolic Syndrome: The Role of AMPK

**DOI:** 10.3390/antiox14070843

**Published:** 2025-07-09

**Authors:** Miroslava Kvandova, Anna Zemancikova, Andrea Berenyiova, Iveta Waczulikova, Silvia Magyarova, Andrea Micurova, Jozef Torok, Marian Grman, Lenka Tomasova, Anton Misak, Zuzana Vysoka, Martina Manikova, Milan Zvarik, Patrick Mydla, Jana Vlkovicova, Peter Balis, Angelika Puzserova

**Affiliations:** 1 Institute of Normal and Pathological Physiology, Centre of Experimental Medicine, Slovak Academy of Sciences, Dúbravská cesta 9, 841 04 Bratislava, Slovakia; anna.zemancikova@savba.sk (A.Z.); andrea.berenyiova@savba.sk (A.B.); silvia.magyarova@savba.sk (S.M.); andrea.micurova@savba.sk (A.M.); jozef.torok@savba.sk (J.T.); 1zuzana.vysoka@gmail.com (Z.V.); manikova.mata@gmail.com (M.M.); peter.balis@savba.sk (P.B.); angelika.puzserova@savba.sk (A.P.); 2 Institute of Pathophysiology, Faculty of Medicine, Comenius University Bratislava, Sasinkova 4, 811 08 Bratislava, Slovakia; 3 Faculty of Mathematics, Physics and Informatics, Comenius University Bratislava, Mlynská dolina F1, 842 48 Bratislava, Slovakia; iveta.waczulikova@fmph.uniba.sk (I.W.); milan.zvarik@fmph.uniba.sk (M.Z.); patrick.mydla@fmph.uniba.sk (P.M.); 4 Institute of Clinical and Translational Research, Biomedical Research Center, Slovak Academy of Sciences, Dúbravská cesta 9, 845 05 Bratislava, Slovakia; marian.grman@savba.sk (M.G.); lenka.tomasova@savba.sk (L.T.); anton.misak@savba.sk (A.M.); 5 Faculty of Chemical and Food Technology, Slovak University of Technology in Bratislava, Radlinského 9, 812 37 Bratislava, Slovakia; 6 Institute for Heart Research, Centre of Experimental Medicine, Slovak Academy of Sciences, Dúbravská cesta 9, 841 04 Bratislava, Slovakia; jana.vlkovicova@savba.sk

**Keywords:** AMPK, endothelial dysfunction, estrogen signalling, metabolic syndrome, inflammation, oxidative stress, sexual dimorphism

## Abstract

Metabolic syndrome (MetS) increases the risk of cardiovascular disease development, with sex differences playing a significant role. AMP-activated protein kinase (AMPK), a key regulator of cellular energy homeostasis, becomes dysregulated in MetS, making it a potential therapeutic target. Therefore, we aimed to investigate the role of AMPK in the development of cardiovascular comorbidities in male and female rats with MetS. MetS was induced in young Wistar–Kyoto (WKY) rats through a high-fat diet (HFD; 10 weeks), and the function of AMPK was studied using Compound C (Cmpd C; 1.5 mg/kg, twice per week, during the last 4 weeks). An HFD induced MetS in males, but, in females, it did not affect body weight, blood pressure, or glycemia until AMPK inhibition occurred. Endothelial dysfunction, oxidative stress, and inflammation developed in both HFD male groups, while, in females, these arose only with AMPK inhibition. In both sexes, α1-AMPK activation decreased with eNOS and Nrf2 protein levels after HFD + Cmpd C treatment. Estradiol levels significantly dropped in HFD and Cmpd C females, whereas testosterone levels remained unchanged. Our results suggest that MetS and related cardiovascular comorbidities in males are driven by oxidative stress, inflammation, and endothelial dysfunction, with minimal additive effect of AMPK. In females, MetS arose only when inhibition of AMPK impaired estrogen signalling, emphasising their protective roles. Targeting AMPK-estrogen pathways may provide a therapeutic strategy, particularly for high-risk cardiovascular females and menopausal women.

## 1. Introduction

Metabolic syndrome (MetS) is a complex cluster of interrelated risk factors that significantly increase the likelihood of developing cardiovascular diseases (CVDs) and type 2 diabetes mellitus. The prevalence of MetS is rising globally, posing a substantial public health challenge due to its association with increased morbidity and mortality rates from cardiovascular events [1]. MetS induced by a high-fat diet (HFD) promotes oxidative stress, which leads to endothelial dysfunction (ED) due to reduced nitric oxide (NO) bioavailability, increased inflammatory cytokines, and impaired antioxidant defences, which contribute to vascular inflammation [2]. Research has demonstrated significant sex-related differences in both the manifestation and consequences of MetS [3]. These differences are apparent in the onset and prevalence of CVDs throughout life. Men generally exhibit higher rates of MetS at younger ages, while women tend to have a higher prevalence in post-menopause [4]. Estrogen has been shown to provide protective cardiovascular effects in premenopausal women, which may account for their lower risk of CVD compared to men at a similar age [5]. Thus, diagnosing and treating CVDs requires sex-specific management due to inherent differences in cardiovascular physiology between sexes. Identifying critical factors contributing to cardiovascular protection is key to improving primary and secondary prevention strategies. One such factor may be adenosine monophosphate-dependent protein kinase (AMPK), which has been demonstrated to play a protective role in the cardiovascular system [6]. Notably, AMPK is regulated by sex hormones, including estrogen [7] and testosterone [8], highlighting its potential involvement in sex-specific cardiometabolic protection. AMPK and sex hormones are interconnected through a reciprocal regulatory relationship. Sex hormones activate AMPK via phosphor-rylation mediated by upstream kinases such as LKB1 or CaMKK2. Conversely, AMPK regulates sex hormone production by suppressing hypothalamic Kiss1 expression, which subsequently leads to reduced GnRH secretion and gonadotropin release. Moreover, AMPK directly inhibits steroid hormone biosynthesis in the gonads and influences sex hormone receptor expression and activity [9]. Given its unique biological properties, AMPK serves as a crucial mediator between metabolic stress and CVD pathogenesis. Therefore, this study focuses on the protective role of AMPK against cardiovascular comorbidities triggered by the development of MetS.

## 2. Materials and Methods

### 2.1. Animal Model

All animal experiments were conducted in accordance with the European Guidelines on Laboratory Animal Care and aligned with the Federation of European Laboratory Ani-mal Science Associations (FELASA) guidelines. The animal study protocol was approved by the Ethics Committee of the Centre of Experimental Medicine of the Slovak Academy of Sciences (EC/CEM/2022/4, dated September 2022; and EC/CEM/2024/3, dated March 2024) and the State Veterinary and Food Administration of the Slovak Republic (Record No. 8704/2022, File No. 6023/2022-220, date of approval 11 October 2022; Record No. 3380/2024, File No. 5634/2024-220, date of approval 18 April 2024).

MetS was induced in 8-week-old male and female Wistar–Kyoto (WKY) rats by feeding a high-fat diet (HFD; ssniff, Soest, Germany; #E15721-34) containing 42% of total ener-gy from fat (21.1% of the total food composition) and 0.21% cholesterol ad libitum for 10 weeks. The role of AMPK was investigated using the inhibitor Compound C (Cmpd C; 1.5 mg/kg, administered intraperitoneally twice weekly for 4 weeks; Calbiochem, San Diego, CA, USA). Control (CTR) animals were maintained on a standard diet. All animals were treated consistently with the same vehicle preparation. Specifically, both the CTR + DMSO and HFD + DMSO groups were administered with the vehicle solution (10% DMSO; Merck KGaA, Darmstadt, Germany) alone, without the active Cmpd C. Refer to the treatment protocol scheme (Supplementary Figure S1) for further details.

Animals were euthanised by isoflurane overdose followed by decapitation. Blood and tissues (aorta, femoral and mesenteric artery, retroperitoneal white adipose tissue (rWAT), and liver) were collected.

### 2.2. Blood Pressure Measurement

Non-invasive systolic blood pressure (sBP) measurement was performed using the plethysmography technique with the CODA system (Kent Scientific Corporation, Torrington, CT, USA). To minimise stress-related variability, measurements were conducted following repeated training sessions.

### 2.3. Determination of Plasma Glucose and Triglyceride Levels and Oral Glucose Tolerance Test

Following the manufacturer’s protocol, glucose and triglyceride (TAG) levels were measured in K_2_EDTA plasma using the Celercare^®^ M5 Chemistry Analyser (Tianjin MNCHIP Technologies Co., Ltd, Tianjin, China) with MNCHIP Celercare—General Chemistry IV.

The oral glucose tolerance test (OGTT) was performed as described in [10]. The test was conducted one day before the sacrifice of fasting animals (8 h). A glucose solution (1.5 g/kg; Merck KGaA, Darmstadt, Germany) was administered via oral gavage (10 μL/g). Blood glucose levels were measured using a glucometer (Roche ACCU-CHECK Aviva, Indianapolis, IN, USA) in tail vein blood at 0, 15, 30, 60, 90, and 120 min post-administration of glucose.

### 2.4. Endothelial Function

The endothelial function was measured using isometric tension studies in the large conductance artery, specifically the thoracic aorta, within an organ bath setup. Endothelium-dependent relaxation of intact rat aortic rings (3 mm in length, devoid of perivascular fat and connective tissue) was evaluated by applying acetylcholine (Ach, 10^−10^–10^−5^ mol/L; Merck KGaA, Darmstadt, Germany) following pre-constriction with noradrenaline (NA, 10^−6^ mol/L; Zentiva, Prague, Czech Republic). In NA-contracted (and in the case of the femoral artery in serotonin (Ser)-contracted) rings with endothelium, Ach induces relaxations that, in some cases, are blunted at higher concentrations, indicating the release of endothelium-derived contracting factors (EDCFs). To determine EDCF production, the maximal response and the response at higher Ach concentration at a particular response curve were compared. Isometric tension of the aorta was measured using a sensor (FSG-01, MDE GmbH, Budapest, Hungary) connected to a NI USB-6221 AD converter (MDE GmbH, Budapest, Hungary), and data acquisition was facilitated through the S.P.E.L. Advanced Kymograph software (version 3.97; MDE GmbH, Budapest, Hungary) [11].

Vascular parameters were also evaluated in freshly isolated and cleaned femoral and mesenteric arteries with intact endothelium. The artery segments were positioned in the chambers of wire myographs (Dual Wire Myograph system 410A and Multi Myograph System 620M and 630MA, Danish Myo Technology A/S, Aarhus, Denmark), with vascular reactivity monitored via LabChart 8 software (ADInstruments NZ Limited, Dunedin, New Zealand). The chambers were filled with physiological saline solution (PSS, in mmol/L: 119 NaCl, 4.7 KCl, 1.17 MgSO_4_·7H_2_O, 25 NaHCO_3_, 1.18 KH_2_PO_4_, 0.03 Na_2_EDTA, 2.5 CaCl_2_·2H_2_O, 5.5 glucose, 37 °C, pH 7.4; Merck KGaA, Darmstadt, Germany) and aerated with 95% O_2_ and 5% CO_2_.

Experimental protocols were carried out as detailed elsewhere [12]. In brief, endothelium-dependent relaxations induced by Ach (1 nmol/L to 10 μmol/L) were measured in femoral arteries pre-contracted withSer (1 μmol/L; Merck KGaA, Darmstadt, Germany) and in mesenteric arteries pre-contracted with NA (10 μmol/L). After washout and stabilisation, the activity of nitric oxide (NO) synthase was inhibited by adding the non-specific inhibitor N^G^-nitro-L-arginine methyl ester (L-NAME, 300 μmol/L, 25 min; Merck KGaA, Darmstadt, Germany) to the chamber. In both groups, Ach-induced relaxation was remeasured following constriction, as done previously, and Ach-induced relaxation was observed again. In the final step, after a PSS wash and a 20 min stabilisation period, the vascular segments were constricted once more, and endothelium-independent relaxation was tested using the exogenous NO donor sodium nitroprusside (SNP, 1 nmol/L to 10 μmol/L; Merck KGaA, Darmstadt, Germany).

### 2.5. Protein and mRNA Expression Analysis

Western blot analysis was employed to determine changes in protein expression. Aortic tissue was homogenised in a cell lysis buffer containing 2 mmol/L Tris-HCl, 250 mmol/L saccharose, 3 mmol/L EGTA, 20 mmol/L EDTA, 0.5 mmol/L PMSF, 1% Triton-X100, 0.5 mmol/L sodium vanadate, 2.5 mmol/L sodium fluoride, a protease inhibitor cocktail (P8340; Merck KGaA, Darmstadt, Germany), and a 1% phosphatase inhibitor cocktail (P2850; Merck KGaA, Darmstadt, GermanyProtein samples (20 μg per lane) were separated by SDS-PAGE and transferred to nitrocellulose membranes. Polyclonal rabbit anti-α1-AMPK (Cell Signalling, #2532, Boston, MA, USA; 1:1000), monoclonal rabbit anti-Cata-lase (Cell Signalling, #14097, Boston, MA, USA; 1:1000), monoclonal rabbit anti-eNOS (Cell Signalling, #32027, Boston, MA, USA; 1:1000), monoclonal rabbit anti- heme oxygen-nase 1 (HO-1; Cell Signalling, #82206, Boston, MA, USA; 1:1000), monoclonal ra-bbit anti-Nrf2 (Cell Signalling, #33649, Boston, MA, USA; 1:1000), polyclonal rabbit anti-p-α1-AMPK (Thr172) (Cell Signalling, #2531, Boston, MA, USA; 1:1000), and monoclonal rabbit anti-SOD1 (Cell Signalling, #37385, Boston, MA, USA; 1:1000) antibodies were used for protein expression analysis. All samples were normalised to the α-Actinin (polyclonal rabbit anti-α-Actinin; Cell Signalling, #3134, Boston, MA, USA; 1:1000). Data were evaluated using ChemiDoc Imaging System (version 1.0.0.15; Bio-Rad, Hercules, CA, USA) and Image Lab Software (version 5.2 build 14; Bio-Rad, Hercules, CA, USA).

Changes in mRNA expression were determined by qRT-PCR as described previously [13]. Briefly, the total RNA of the samples was isolated by the acid guanidinium thiocyanate-phenol method [14]. The total RNA was spectrophotometrically quantified at 260/280 nm and 260/230 nm using a NanoDrop spectrophotometer (Thermo Scientific, Waltham, MA, USA). In the next step, the isolated RNA was reverse transcribed into cDNA using the Eppendorf Mastercycler (Eppendorf AG, Elbmarsch, Germany) and the iScript™ cDNA Synthesis Kit (Bio-Rad, Hercules, CA, USA) reaction mixture, according to the manufacturer’s instructions. Gene amplification was performed using qPCR on a CFX96 Real-Time PCR detection system (Bio-Rad, Hercules, CA, USA). Sso Advanced Universal SYBR Green Supermix (Bio-Rad, Hercules, CA, USA) was used for gene amplification. All samples were normalised to β-Actin as an internal control. The comparative ΔΔCt method was used for quantification of the relative mRNA expression. Gene expre-ssion of the target gene in each sample was expressed as the percentage of m_CTR. The qPCR primer sequences were as follows: *-Actb*_forward: CTC TGT GTG GAT TGG TGG CT, *Actb*_reverse: CGC AGC TCA GTA ACA GTC CG; *Nos3* _forward: GAT CCC CCG GAG AAT GGA GA, *Nos3*_reverse: TCG GAT TTT GTA ACT CTT GTG CT; *Cox2*_forward: CTA CCA TCT GGC TTC GGG AG, *Cox2*_reverse: TGG AAC AGT CGC TCG TCA TC; *Il1b*_forward: CAC CTC TCA AGC AGA GCA CAG, *Il1b*_reverse: GGG TTC CAT GGT GAA GTC AAC; *Inos*_forward: AAA CGC TAC ACT TCC AAC GC, *Inos*_reverse: TGC TGA GAG CTT TGT TGA GGT C; *Tnf*_forward: CGT CAG CCG ATT TGC CAT TTC, *Tnf*_reverse: TGG GCT CAT ACC AGG GCT T.

### 2.6. Oxidative Stress

The aortic production of superoxide was measured using a high-performance liquid chromatography (HPLC)-based dihydroethidium (DHE; Merck KGaA, Darmstadt, Germany) assay as described previously [15].

SOD (superoxide dismutase) activity was determined in plasma and mitochondria samples using Superoxide Dismutase, SOD, Activity Assay Kit (CS0009-1KT, Merck KGaA, Darmstadt, Germany) and following the manufacturer’s protocol.

### 2.7. Sex Hormone Level

The concentrations of sex hormones were quantified in plasma samples using Rat E2 (Estradiol) ELISA Kit (ER1507, FineTest, Wuhan, China) and Rat T (Testosterone) ELISA Kit (ER1462, FineTest, Wuhan, China), following the manufacturer’s protocol.

### 2.8. Mitochondrial Function

Liver mitochondria were isolated using a modified method described by Gizatullina, et al. [16]. Used isolation buffer: 250 mmol/L sucrose, 20 mmol/L MOPS, 1 mmol/L EGTA, pH 7.2 adjusted by KOH and supplemented with 0.1% *w*/*v* BSA (all from Merck KGaA, Darmstadt, Germany) in the first and second steps of isolation and centrifugation. All steps were carried out at 4 °C. Briefly, the liver was minced using scissors into small pieces. The mash was washed a few times with isolation buffer with BSA to remove blood during this procedure. At the end, the final mash was homogenised by a Potter–Elvehjem homogeniser with six strokes at ~400 rpm. The homogenate (5–10 mL of isolation buffer per gram of tissue) was centrifuged at 940× *g* for 9 min. The obtained supernatant was transferred to a new precooled tube and centrifuged at 8500× *g* for 18 min at 4 °C. The pellet was resuspended in isolation buffer without BSA and centrifuged at 8500× *g* for 18 min at 4 °C. Finally, the obtained pellet was resuspended in a small volume of isolation buffer without BSA (100 μL/g of tissue). The BCA protein assay determined the total final protein mass (mg/mL). Mitochondrial preparations were used within four hours.

#### 2.8.1. Measurement of Mitochondrial Respiration

Functional parameters of isolated mitochondria were estimated polarographically by the Mitocell MS200A system (Strathkelvin Instruments, North Lanarkshire, Scotland). Mitochondrial respiration was measured in a respiration buffer MiR05 (110 mmol/L D-sucrose, 60 mmol/L Lactobionic acid, 20 mmol/L Taurine, 10 mmol/L KH_2_PO_4_, 20 mmol/L HEPES, 3 mmol/L MgCl_2_•6H_2_O, 0.5 mmol/L EGTA, ~KOH, final pH 7.1; Merck KGaA, Darmstadt, Germany) in the presence of a combination of various substrates (10 mmol/L glutamate + 5 mmol/L malate or 10 mmol/L succinate + 2 μmol/L rotenone as inhibitor of Complex I; Merck KGaA, Darmstadt, Germany). Next, 200 μmol/L ADP (Merck KGaA, Darmstadt, Germany) was added to induce state 3 (phosphorylation of ADP to ATP). State 4 was evaluated as the respiration rate after adding 200 μmol/L ADP, which was depleted. Respiration rate was calculated as the negative slope of oxygen concentration and normalised to the protein mass of added mitochondria. All measurements were performed at 30 °C.

#### 2.8.2. Mitochondrial ROS Production

The production of superoxide was determined in mitochondria samples using MitoSOX Red (M36009, ThermoFisher, Waltham, MA, USA) and following the manufacturer’s protocol.

### 2.9. Statistical Analysis

Statistical analysis was performed using Prism for Windows, version 10 (GraphPad Software Inc., La Jolla, CA, USA). Two-way ANOVA followed by Fisher’s LSD post hoc test was used to compare multiple group means. For all concentration–response curves (repeated measures) involving four groups and three independent factors (HFD, AMPK inhibition by Cmpd C, and sex), a four-parameter logistic (4PL) nonlinear regression ana-lysis was applied. EC_50_ values derived from acetylcholine (Ach) or SNP concentration–response curves were compared using the Tukey–Kramer multiple comparisons test, provided that the data distribution appeared symmetrical and the test for homogeneity of variances was non-significant. If these assumptions were violated, the nonparametric Kruskal–Wallis test was used instead. All results are expressed as mean ± SEM, and statistical significance was considered at *p* < 0.05. To emphasise sex-related differences, all measured data (male and female) are presented relative to the male control group, which serves as the common baseline due to its widespread use in previous studies.

## 3. Results

### 3.1. Induction of MetS in Male and Female Rats—Role of AMPK

All experimental animals were fed an HFD to induce MetS. Administration of the HFD to young male WKYs resulted in a significant elevation in body weight (BW) (Supplement Figure S2A), the ratio of rWAT to BW (Supplementary Figure S2C), sBP (Supplementary Figure S2E), glycemia (Supplementary Figure S2I), and the development of insulin resistance (Supplementary Figure S2K). Plasma TAG levels in HFD-fed animals showed an upward trend but did not reach statistical si-gnificance (Supplementary Figure S2G). AMPK inhibition with Cmpd C had no additive or only minimal effects on the metabolic alterations induced by HFD in male WKYs.

In contrast, in female WKYs, HFD significantly increased only the rWAT/BW ratio. Meanwhile, other metabolic parameters, except for TAG concentration, remained largely unaffected, suggesting a female-dependent protective mechanism against MetS development. However, when AMPK was inhibited during HFD, treatments resulted in the full manifestation of MetS features in females (Supplementary Figure S2).

Our results indicated sex-specific differences in nearly all analysed parameters, except for rWAT/BW ratio and glycemia, where statistically significant differences were observed only between experimental groups, not sexes (Supplementary Figure S12, Supplementary Table S2). Moreover, BW, TAG level, and glycemia showed a significant interaction, indicating a differential metabolic response to HFD between males and females (Supplementary Figure S12, Supplementary Table S2).

### 3.2. Induction of Endothelial Dysfunction in Male and Female Rats—Role of AMPK

**Figure 1:**
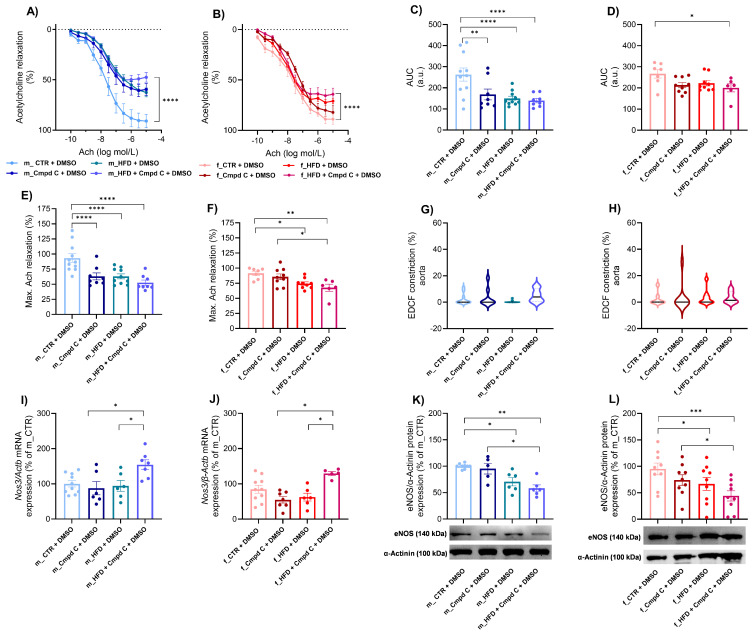
Induction of ED in the aorta in male and female rats—role of AMPK. The development of ED was analysed in intact rat aortic rings using an organ bath system and expressed as an Ach response curve in (**A**) young male WKYs (n= 8–11) and (**B**) young female WKYs (n = 6–9); an AUC of Ach curve in (**C**) young male WKYs (n = 8–11) and (**D**) young female WKYs (n = 6–9); and maximal Ach relaxation in (**E**) young male WKYs (n = 8–11) and (**F**) young female WKYs (n = 6–9). From the Ach relaxation curves, the EDCF constriction was calculated in (**G**) young male WKYs (n = 8–11) and (**H**) young female WKYs (n = 6–9). The expression of eNOS was determined by qRT-PCR in aortic tissue in (**I**) young male WKYs (n = 6–10) and (**J**) young female WKYs (n = 5–10) or by Western blot in (**K**) young male WKYs (n = 5–9) and (**L**) young female WKYs (n = 9). Data are presented as mean ± SEM. *p*-values < 0.05; were considered significant; * *p* ≤ 0.05, ** *p* ≤ 0.01, *** *p* ≤ 0.001, **** *p* ≤ 0.0001. Actb—β-actinin; *Cmpd C—Compound C*; *CTR—control rats*; *EDCF—endothelium-derived contracting factors*; *eNOS—endothelial nitric oxide synthase (protein)*; *HFD—high-fat diet*; *Nos3—endothelial nitric oxide synthase (gene)*.

HFD treatment induced ED in the aorta of male rats, with only minimal additional effects observed upon AMPK inhibition (Figure 1A,C), accompanied by reduced maximal Ach-induced relaxation (Figure 1E). Similarly, Cmpd C reduced the area under the curve (AUC) and maximal Ach relaxation in males. In contrast, female rats did not develop ED in response to HFD alone. However, simultaneous AMPK inhibition du-ring HFD exposure also resulted in the manifestation of ED in females. Interestingly, in females, maximal relaxation was already reduced by HFD alone (Figure 1F). EDCF-dependent constriction remained unchanged in both sexes (Figure 1G,H). Statistical analysis demonstrated that HFD and AMPK inhibition exert significant independent effects between sexes (Supplementary Figure S5).

ED was associated with increased *Nos3* (endothelial nitric oxide synthase gene) mRNA expression in the aortic tissue of both sexes following combined HFD and AMPK inhibition (Cmpd C treatment) (Figure 1I,J). In contrast, eNOS (endothelial nitric oxide synthase protein) protein expression was reduced in both male and female rats subjected to HFD or HFD + Cmpd C treatment (Figure 1K,L). Our findings confirm that HFD and AMPK inhibition significantly affect *Nos3* mRNA expression, and that sex differences exist in this effect. In contrast, alterations in eNOS protein expression were attributable primarily to treatment effects, rather than sex or a sex-by-treatment interaction (Supplementary Table S2).

**Figure 2:**
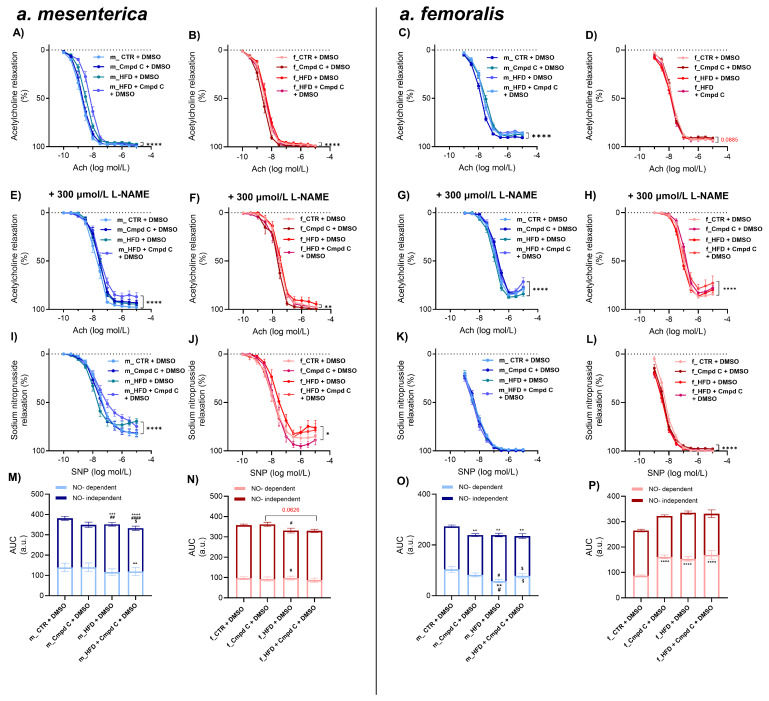
Induction of ED in mesenteric and femoral arteries in male and female rats—role of AMPK. The development of ED was analysed by Ach response curve in intact rat small mesenteric artery of (**A**) young male WKYs (n = 12–16) and (**B**) young female WKYs (n = 9–15), and in intact rat femoral artery of (**C**) young male WKYs (n = 14–18) and (**D**) young female WKYs (n = 13–14). NO-independent components of Ach-induced relaxation were analysed using the non-specific inhibitor of NOS L-NAME (300 μmol/L) in mesenteric artery of (**E**) young male WKYs (n = 12–16) and (**F**) young female WKYs (n = 9–15), and in femoral artery of (**G**) young male WKYs (n = 14–18) and (**H**) young female WKYs (n = 13–14). Endothelium-independent relaxation was analysed using SNP in mesenteric artery of (**I**) young male WKYs (n = 12–16) and (**J**) young female WKYs (n = 9–15), and in femoral artery of (**K**) young male WKYs (n = 14–18) and (**L**) young female WKYs (n = 13–14). The NO-dependent and NO-independent components of Ach-induced relaxation were calculated and expressed as AUC in the mesenteric artery of (**M**) young male WKYs (n = 12–16) and (**N**) young female WKYs (n = 9–15) and in the femoral artery of (**O**) young male WKYs (n = 14–18) and (**P**) young female WKYs (n = 13–14). Data are presented as mean ± SEM. *p*-values <0.05 were considered significant; * *p* ≤ 0.05, ** *p* ≤ 0.01, *** *p* ≤ 0.001, **** *p* ≤ 0.0001. * vs. CTR, # *p* ≤ 0.05, ## *p* ≤ 0.01, #### *p* ≤ 0.0001 vs. Cmpd C, $ vs. HFD. *Ach—acetylcholine*; *a.u.—arbitrary units*; *AUC—area under the curve*; *Cmpd C—Compound C*; *CTR—control rats*; *L-NAME—N^G^-nitro-L-arginine methyl ester*; *NO—nitric oxide*; *HFD—high-fat diet*; *SNP—sodium nitroprusside*.

HFD treatment also impaired endothelial function in the small mesenteric arteries of male WKY rats, with minimal additive effects of AMPK inhibition (Figure 2A,M). In these arteries, nitric oxide (NO)-dependent and NO-independent relaxation responses were not significantly altered by HFD alone (Figure 2M). However, AMPK inhibition during HFD treatment significantly impaired NO-independent relaxation. In contrast, female rats did not exhibit ED in mesenteric arteries in response to either HFD alone or to combined HFD and AMPK inhibition compared to their respective controls (Figure 2N). These results indicate that the effects of HFD and AMPK inhibition on total Ach-induced relaxation were sex-dependent. However, no consistent sex-specific differences were observed across all experimental conditions (Supplementary Figure S6).

In the femoral artery, vascular reactivity was significantly impaired in male WKY rats treated with Cmpd C, HFD, or both (Figure 2C,O). HFD alone impaired the NO-independent relaxation component compared to the Cmpd C group, and NO-dependent relaxation was diminished compared to the control. Inhibition of AMPK during HFD altered the balance between NO-dependent (increased) and NO-independent (decreased) relaxation components compared to HFD alone.

In contrast, female rats did not develop femoral artery ED in response to an HFD alone (Figure 2D). Interestingly, in females, inhibition of AMPK and combined inhibition of AMPK and HFD led to an increase in the NO-dependent relaxation component (Figure 2P). Changes in total AUC induced by HFD and AMPK inhibition were sex-dependent, with males and females responding differently to the combined interventions. However, no uniform main effect of treatment alone was detected across all groups (Supplementary Figure S6, Supplementary Table S2).

Maximal relaxation was reduced only in the male femoral artery in HFD and HFD + Cmpd C groups (Supplementary Figure S3C). NOS inhibition by L-NAME further contributed to the reduction of maximal relaxation in the mesenteric artery in male HFD, combined with AMPK inhibition (Supplementary Figure S3E). In contrast, in the femoral artery, this occurred only in female HFD + Cmpd C (Supplementary Figure S3H). EDCF-dependent contraction was observed in the mesenteric artery only in male HFD + Cmpd C when NOS was inhibited (Supplementary Figure S3I,M). In the male femoral artery, EDCF production was significantly elevated by AMPK inhibition in both groups, with the most pronounced effect observed in the HFD + Cmpd C group (Supplementary Figure S3K). NOS inhibition further emphasised this effect (Supplementary Figure S3O).

Finally, the cumulative addition of the NO, SNP eli-cited similar relaxation responses in the mesenteric and femoral arteries across all experimental groups (Figure 2I–L).

### 3.3. Induction of Vascular Oxidative Stress in Male and Female Rats—Role of AMPK

**Figure 3:**
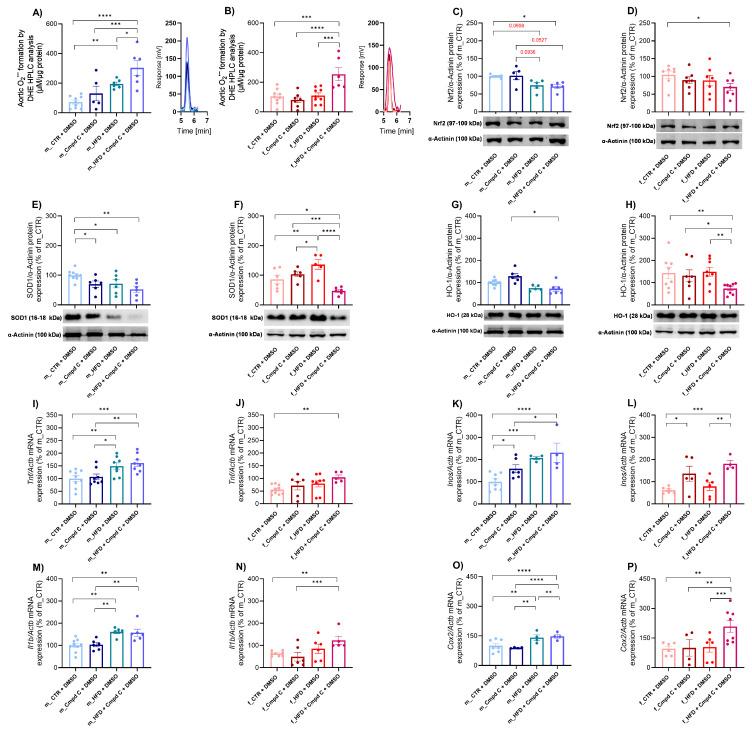
Induction of vascular oxidative stress and inflammation in male and female rats—role of AMPK. Vascular oxidative stress development was evaluated by analysing oxidative stress mar-kers. Superoxide production in rat aortic rings was measured using DHE-HPLC analysis for (**A**) young male WKYs (n = 5–9) and (**B**) young female WKYs (n = 6–9), with data shown through chromatograms. The protein expression was analysed by Western blot as follows. Aortic Nrf2 protein expression in (**C**) young male WKYs (n = 7–9) and (**D**) young female WKYs (n = 6); aortic SOD1 protein expression in (**E**) young male WKYs (n= 5–9) and (**F**) young female WKYs (n = 5–6) and aortic HO-1 protein expression in (**G**) young male WKYs (n = 5–9) and (**H**) young female WKYs (n = 7–8). The expression analysis of inflammatory markers assessed the development of vascular inflammation in the rat aortic tissue. The expression of *Tnf* mRNA was determined by qRT-PCR in aortic tissue in (**I**) young male WKYs (n = 8–9) and (**J**) young female WKYs (n = 5–10); aortic *Inos* mRNA in (**K**) young male WKYs (n = 4–8) and (**L**) young female WKYs (n = 4–6); aortic *Il1b* mRNA in (**M**) young male WKYs (n = 6–9) and (**N**) young female WKYs (n = 5–8); and aortic Cox2 mRNA in (**O**) young male WKYs (n = 4–7) and (**P**) young female WKYs (n = 4–8). Data are presented as mean ± SEM. *p*-values < 0.05 were considered significant; * *p* ≤ 0.05, ** *p* ≤ 0.01, *** *p* ≤ 0.001, **** *p* ≤ 0.0001. *Cmpd C—Compound C*; *Cox2—cyclooxygenase-2*; *CTR—control rats*; *DHE—dihydroethidium*; *HFD—high-fat diet*; *HO-1—heme oxygenase 1*; *HPLC—high-performance liquid chromatography*; *Il1b—interleukin 1-β*; *Inos—inducible nitric oxide synthase*; *Nrf2—nuclear factor erythroid 2-related factor 2*; *SOD1—superoxide dismutase 1*; *Tnf—tumour necrosis factor-alpha*.

We demonstrated that a 10-week HFD significantly increased aortic superoxide production in males, with an additive effect of AMPK inhibition (Figure 3A). In contrast, females did not exhibit elevated superoxide production in response to HFD alone; however, a significant increase was observed when HFD was combined with AMPK inhibition (Figure 3B). These findings reveal a sex-dependent pattern of superoxide production; however, no consistent main effect of treatment group was observed (Supplementary Figure S7).

Furthermore, the applied interventions modulated the nuclear factor erythroid 2-related factor 2 (Nrf2)-dependent antioxidant defence system. In male WKY rats, both HFD (by trend) alone and HFD combined with Cmpd C reduced aortic Nrf2 protein expression (Figure 3C). A significant decrease in Nrf2 protein expression occurred in females only when HFD was administered with AMPK inhibition (Figure 3D). HFD and/or AMPK inhibition significantly influenced Nrf2 protein expression regardless of sex, with no evidence of sex-specific effects or interaction between sex and treatment (Supplementary Figure S7, Supplementary Table S2).

To further assess systemic antioxidant capacity, we evaluated total superoxide dismutase (SOD) activity in plasma samples, along with the expression of SOD1 protein. SOD1 protein expression was significantly decreased in males in all treatment groups (Figure 3E). In females, the HFD increased SOD1 expression, while a decrease in SOD1 expression was observed only in the HFD group treated with the AMPK inhibitor (Figure 3F). The effects of HFD and AMPK inhibition on SOD1 protein expression exhibited sex-dependent and interactive characteristics, indicating that the combination of sex and treatment produces unique, non-additive responses (Supplementary Table S2). Moreover, both male and female WKY rats demonstrated decreased total SOD activity following treatment with Cmpd C, either alone or in combination with HFD (Supplementary Figure S4). Alterations in plasma SOD activity induced by HFD and/or AMPK inhibition were similar across sexes, with no detectable sex-specific differences or interaction (Supplementary Figure S7, Supplementary Table S2).

Additionally, the combination of HFD and AMPK inhibition significantly reduced aortic heme oxygenase-1 (HO-1) protein expression in both sexes, whereas HFD alone did not affect HO-1 levels (Figure 3G,H). Both sex and treatment independently modulated HO-1 expression (Supplementary Figure S7, Supplementary Table S2).

### 3.4. Induction of Vascular Inflammation in Male and Female Rats—Role of AMPK

The expression levels of various inflammatory markers were modulated by HFD and HFD combined with Cmpd C in a sex-specific manner. A ten-week exposure to HFD resulted in a significant upregulation of *Tnf* mRNA expression in the aortic tissue of male rats, without further modification by AMPK inhibition (Figure 3I). In contrast, HFD alone did not influence *Tnf* expression in females. However, AMPK inhibition during HFD exposure significantly increased *Tnf* mRNA levels in female rats (Figure 3J). Similarly, *Il1b* mRNA expression was significantly elevated in the HFD group of male WKY rats (Figure 3M), whereas in females, HFD alone did not alter *Il1b* mRNA expression (Figure 3N). HFD, AMPK inhibition, and sex independently modulated *Tnf* and *Il1b* mRNA expression. However, no significant sex-treatment interaction was observed (Supplementary Figure S8, Supplementary Table S2).

In male WKY rats, HFD and HFD + Cmpd C significantly increased *Inos* mRNA expression (Figure 3K). In contrast, females showed no significant changes in *Inos* mRNA expression in response to HFD alone (Figure 3L). Both HFD and/or AMPK inhibition, as well as sex, independently influenced *Inos* mRNA expression, without evidence of a sex-treatment interaction (Supplementary Figure S8, Supplementary Table S2).

Similarly, *Cox2* mRNA expression was significantly upregulated in males following HFD exposure, with a further increase observed upon co-administration of Cmpd C. In females, *Cox2* mRNA expression remained unchanged following HFD alone but was si-gnificantly elevated when HFD was combined with AMPK inhibition (Figure 3O,P). The effects of HFD and AMPK inhibition on *Cox2* mRNA expression differed between sexes and treatment groups, suggesting sex-specific regulatory mechanisms and interactions between HFD and AMPK signalling (Supplementary Figure S8, Supplementary Table S2).

### 3.5. Induction of Mitochondrial Dysfunction in Male and Female Rats—Role of AMPK

**Figure 4:**
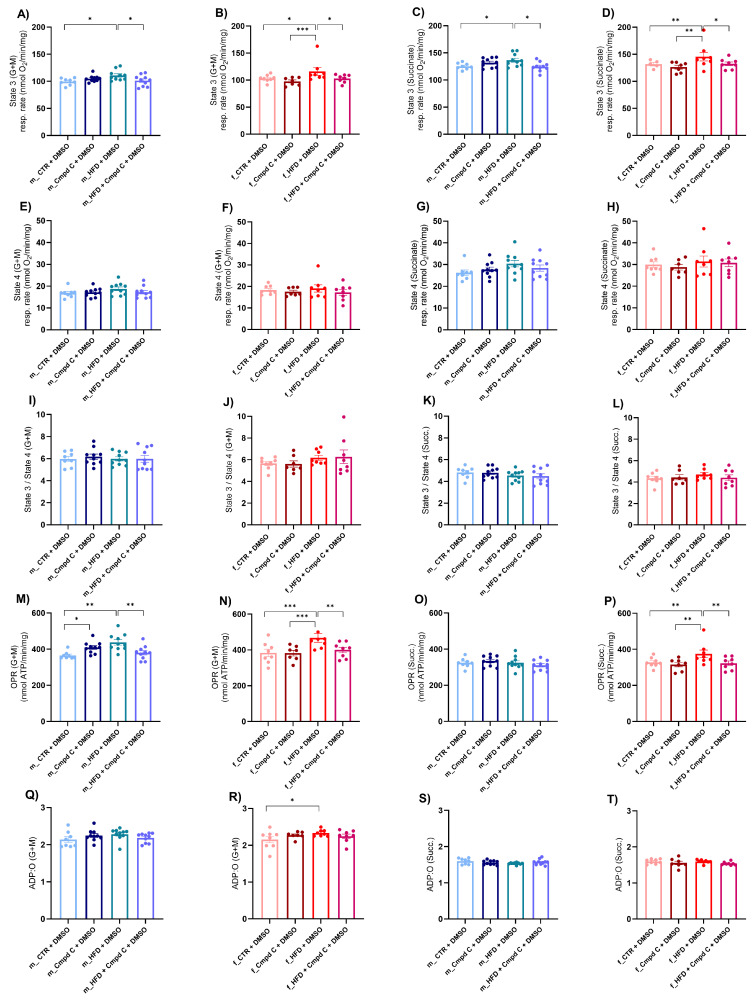
Induction of mitochondrial respiration in male and female rats—role of AMPK. The development of mitochondrial dysfunction was assessed in the mitochondria isolated from the liver. Mitochondrial respiration was analysed as (**A**) State 3 (G + M) in young male WKYs (n = 8–10); (**B**) State 3 (G + M) in young female WKYs (n = 7–8); (**C**) State 3 (Succ.) in young male WKYs (n = 8–10); (**D**) State 3 (Succ.) in young female WKYs (n = 7–8); (**E**) State 4 (G + M) in young male WKYs (n = 8–10); (**F**) State 4 (G + M) in young female WKYs (n = 7–8); (**G**) State 4 (Succ.) in young male WKYs (n = 8–10); (**H**) State 4 (Succ.) in young female WKYs (n = 7–8); (**I**) State 3/4 (G + M) in young male WKYs (n= 8–10); (**J**) State 3/4 (G + M) in young female WKYs (n = 7–8); (**K**) State 3/4 (Succ.) in young male WKYs (n = 8–10); (**L**) State 3/4 (Succ.) in young female WKYs (n = 7–8); (**M**) OPR (G + M) in young male WKYs (n = 8–10); (**N**) OPR (G + M) in young female WKYs (n = 7–8); (**O**) OPR (Succ.) in young male WKYs (n = 8–10); (**P**) OPR (Succ.) in young female WKYs (n = 7–8); (**Q**) ADP:O (G + M) in young male WKYs (n = 8–10); (**R**) ADP:O (G + M) in young female WKYs (n = 7–8); (**S**) ADP:O (Succ.) in young male WKYs (n = 8–10) and (**T**) ADP:O (Succ.) in young female WKYs (n = 7–8). Data are presented as mean ± SEM. *p*-values < 0.05 were considered significant; * *p* ≤ 0.05, ** *p* ≤ 0.01, *** *p* ≤ 0.001. *ADP—Adenosine-5’-diphosphate*; *Cmpd C—Compound C*; *CTR—control rats*; *G—glutamate*; *HFD—high-fat diet*; *M—malate*; *OPR—oxidative phosphorylation rate*; *O—one atom of oxygen*; *Succ.—succinate*.

Next, we assessed mitochondrial function by determining the oxygen consumption rate in isolated mitochondria derived from liver tissue. This evaluation focused on the coupling efficiency between the electron transport chain and the oxidative phosphorylation machinery across distinct respiratory states. We detected an increase in state 3 respiration (oxygen consumption after the addition of 200 μmol/L ADP) supported by glutamate and malate (G + M) (Figure 4A,B), as well as by succinate (Succ.) (Figure 4C,D), in both male and female HFD rats, indicative of enhanced ADP-stimulated oxidative phosphorylation through both complex I- and complex II-mediated pathways. This enhancement may represent either an adaptive or a maladaptive alteration in mitochondrial energy metabolism induced by HFD. Notably, state 3 respiration is stimulated by G + M and Succ. This stimulation was attenuated in both sexes when AMPK activity was inhibited during HFD exposure, underscoring the critical role of AMPK signalling in mitochondrial adaptations to HFD (Supplementary Figure S9).

Conversely, state 4 respiration (respiration after depletion of added ADP) (Figure 4E–H) and the state 3/state 4 ratio (Figure 4I–L) were not significantly influenced by either HFD or AMPK inhibition. Furthermore, the oxidative phosphorylation rate (OPR) measured with both substrate types was elevated in HFD-exposed males and females; however, this increase was absent under conditions of AMPK inhibition (Figure 4M,N,P). The ADP:O ratio was selectively increased in HFD-fed females when G + M served as the substrates, whereas no significant changes were observed with Succ. or in male WKY rats (Figure 4Q–T).

AMPK inhibition and/or HFD significantly modulated state 3 respiration (G + M, Succ.), OPR (G + M), and the ADP:O ratio (G + M) independently of sex, without evidence of sex-specific differences or interaction effects. In contrast, OPR (Succ.) was significantly affected by the interaction between HFD and AMPK inhibition. However, the effects were comparable between males and females, suggesting no marked sex-related divergence in response. These results indicate the activation of mitochondria-specific compensatory mechanisms of respiration in response to HFD, an effect that was abolished by AMPK inhibition.

Additionally, mitochondrial SOD activity was significantly increased in males subjected to HFD and in males co-treated with HFD and Cmpd C (Supplementary Figure S4C). In females, increased SOD activity was evident only in the HFD group under AMPK inhibition (Supplementary Figure S4D). Next, we evaluated mitochondria-specific superoxide production in isolated liver mitochondria using MitoSOX (Supplementary Figure S4E,F). Elevated mitochondrial superoxide production triggered by Cmpd C was observed in male and female WKY rats, whereas no significant alterations were detected in other groups. Together, these findings support the notion that mitochondria-specific compensatory mechanisms are activated in response to HFD, particularly when AMPK signalling is disrupted. The observed differences were attributed solely to treatment effects and were not influenced by interaction between sex and treatment (Supplementary Figure S15, Supplementary Table S2).

### 3.6. Expression of α1-AMPK and Sex Hormone Levels During MetS Development—Role of Sex

**Figure 5:**
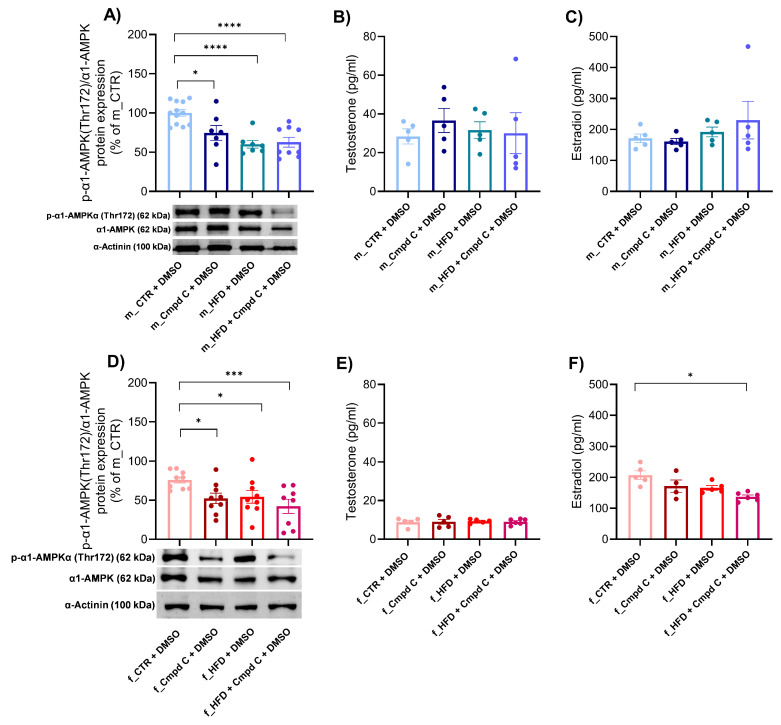
Expression of α1-AMPK and sex hormone levels during MetS development—role of sex. The activation of α1-AMPK via phosphorylation on Thr172 was analysed and expressed as p-α1-AMPK(Thr172)/α1-AMPK ratio in the rat aortic tissue by Western blot analysis in (**A**) young male WKYs (n = 8–12) and (**D**) young female WKYs (n = 9). Sex hormone plasma levels were determined by ELISA, concretely testosterone level in (**B**) young male WKYs (n = 5) and (**E**) young female WKYs (n = 5–6), and estradiol levels in (**C**) young male WKYs (n = 4–5) and (**F**) young female WKYs (n = 4–6). Data are presented as mean ± SEM. *p*-values < 0.05 were considered significant; * *p* ≤ 0.05, *** *p* ≤ 0.001, **** *p* ≤ 0.0001. *α1-AMPK—alpha one adenosine monophosphate-dependent protein kinase*; *Cmpd C—Compound C*; *CTR—control rats*; *HFD—high-fat diet*; *p-α1-AMPK(Thr172)—alpha one adenosine monophosphate-dependent protein kinase phosphorylated at threonine 172*.

We observed a reduction in the activation of the predominant vascular isoform of AMPK, α1-AMPK, in both male and female subjects following treatment with Cmpd C, HFD, and the combination of HFD with AMPK inhibition (Figure 5A,D). Quantitative analysis revealed that the decreased levels of phosphorylated α1-AMPK observed in the HFD + Cmpd C in males and females were not accompanied by a corresponding reduction in total α1-AMPK protein levels. Instead, the phospho/total α1-AMPK ratio was significantly lower compared to other groups, indicating impaired AMPK activation in this condition. Both HFD and/or AMPK inhibition, as well as sex, independently influenced the α1-AMPK activation (Supplementary Figure S11, Supplementary Table S2).

To further investigate the interplay between AMPK signalling and sex hormones, we subsequently analysed plasma sex hormone concentrations. In male rats, plasma estradiol levels remained unchanged (Figure 5C). Conversely, treatment with Cmpd C during HFD feeding in female rats significantly decreased estradiol levels (Figure 5F). Testosterone concentrations were not significantly affected by either HFD, AMPK inhibition, or their combination in any experimental group (Figure 5B,E).

### 3.7. Relation to Estradiol and α1-AMPK Signalling

To examine how physiological parameters associated with pathophysiological processes respond to hormonal and metabolic regulatory signals, we analysed their relationship to changes (log_2_ fold change) in circulating estradiol levels (*X*-axis) and AMPK activity (*Y*-axis), with the magnitude of response represented by log_2_ fold change (bubble size). Relative and absolute (fold) changes were compared across sexes and treatment groups (CTR vs. HFD, and CTR vs. HFD + Cmpd C), allowing us to identify estradiol-associated patterns and AMPK-dependent effects.

**Figure 6:**
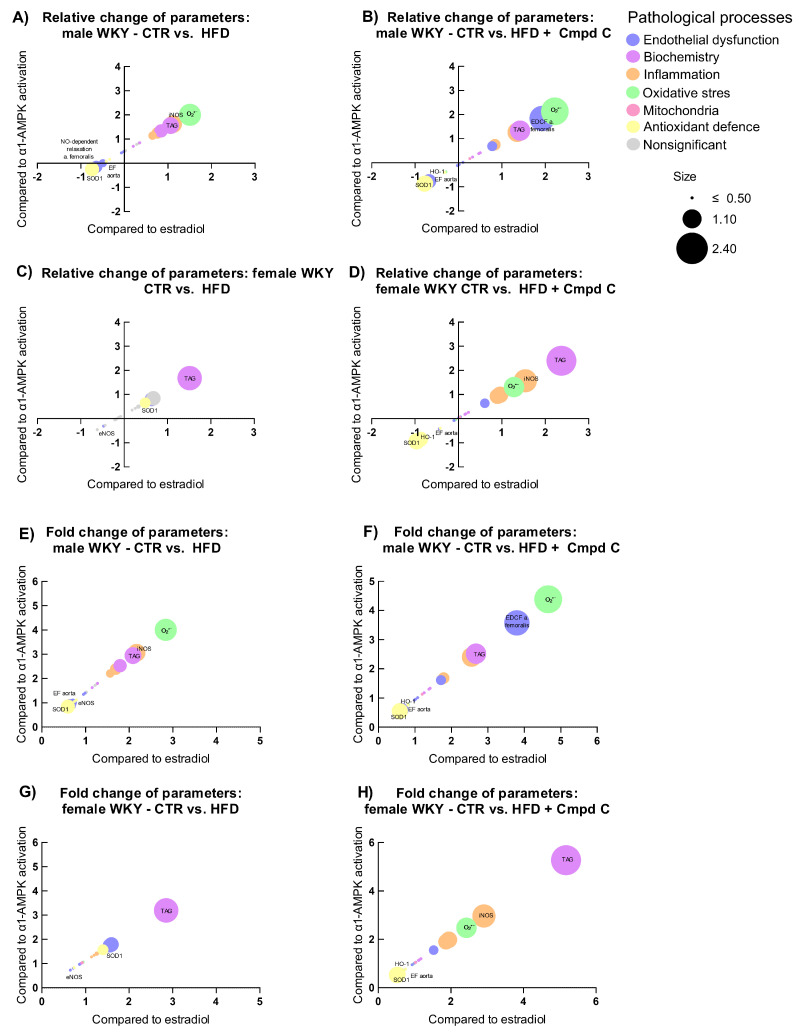
Sex-specific responses of physiological parameters to HFDand AMPK inhibition expressed as relative change induced by (**A**) HFD in young male WKYs; (**B**) HFD + Cmpd C in young male WKYs; (**C**) HFD in young female WKYs; (**D**) HFD + Cmpd C in young female WKYs; and as fold change induced by (**E**) HFD in young male WKYs; (**F**) HFD + Cmpd C in young male WKYs; (**G**) HFD in young female WKYs, and (**H**) HFD + Cmpd C in young female WKYs. Only parameters where sex-dependent significant (*p*-values < 0.05) differences were observed were analysed. *α1-AMPK—alpha one adenosine monophosphate-dependent protein kinase*; *Cmpd C—Compound C*; *CTR—control rats*; *EDCF—endothelium-derived contracting factors*; *EF- endothelial function*; *eNOS—endothelial nitric oxide synthase*; *HFD—high-fat diet; HO-1—heme oxygenase 1*; *iNOS—inducible nitric oxide synthase*; *NO—nitric oxide*; *O_2_•−—superoxide*; *SOD1—superoxide dismutase 1*; *TAG—triglycerides*.

In males, HFD induced clear signs of metabolic dysregulation, oxidative stress, and inflammation (Figure 6A,E), which were further intensified by AMPK inhibition; superoxide levels increased further, and antioxidant enzymes were more strongly suppressed (Figure 6B,F). However, the additive impact of AMPK inhibition appeared modest. In contrast, females showed minimal changes under HFD alone. However, AMPK inhibition abolished this resistance, triggering marked increases in inflammatory and oxidative markers, underscoring a strong AMPK-dependent protective mechanism in females (Figure 6G,H).

## 4. Discussion

Understanding the key factors in cardiovascular protection is crucial for improving prevention strategies. Particular attention should be given to the distinct cardiovascular needs of each sex, as well as the sex-specific development of CVDs associated with MetS. Research indicates that, while the cumulative incidence of CVD increases steadily with age in men, it remains stable in women until menopause, after which it rises sharply [4,5]. MetS manifests differently in men and women due to hormone variations, fat distribution, and inflammatory processes. Estrogen provides premenopausal women with protection against insulin resistance and is associated with higher levels of anti-inflammatory adiponectin. However, this protective effect diminishes after menopause, increasing their risk of developing MetS. Men, on the other hand, tend to accumulate more visceral fat due to the effects of testosterone and exhibit stronger inflammatory responses. Elevated levels of pro-inflammatory cytokines, such as IL-6 and TNF-α, contribute to their increased risk of MetS [17]. During the development of MetS, AMPK activity is reduced. This reduction impairs the enzyme’s role in stimulating glucose uptake, fatty acid oxidation, and inhi-biting lipogenesis and gluconeogenesis. Dysregulated AMPK worsens insulin sensitivity, increases fat accumulation, and causes chronic inflammation. Conversely, activating AMPK through exercise, calorie restriction, or pharmacological agents improves insulin sensitivity and metabolic health. Thus, AMPK dysregulation is both a pathogenic factor in MetS and a promising therapeutic target for its prevention and treatment [18]. Given the established crosstalk between sex hormones, including estrogen [7] and testosterone [8], and AMPK signalling, this study aims to investigate the precise role of this enzyme in the development of MetS.

To date, no study has investigated the cardiovascular role of AMPK in the development and progression of MetS in a sex-dependent manner. Our findings reveal significant sex-specific effects. For the first time, we demonstrate that these differences are closely linked to AMPK signalling, which emerges as a key contributing pathway. HFD induces MetS in male rats, while females are strongly protected against metabolic and cardiovascular damage. This difference is linked to AMPK, which is crucial for cardiovascular health by regulating metabolic homeostasis and protecting against pathological conditions. Beyond metabolism, AMPK regulates cardiovascular-specific pathways, including endothelial function, oxidative stress, inflammation, cardiac metabolism, and the prevention of hypertrophy, thereby serving as a key mediator of cardiovascular protection. Thus, AMPK is a central mediator in both metabolic and cardiovascular health [6,19]. Unexpectedly, we demonstrate that AMPK inhibition has minimal impact on the development of MetS in males, yet plays a pivotal role in females. These results challenge the conventional view of AMPK as a universal metabolic regulator, instead revealing a sex-specific dependency that redefines our understanding of metabolic regulation. This highlights the importance of AMPK in modulating metabolic homeostasis in a sex-specific manner.

We further investigated the impact of MetS on ED development. This dysfunction is primarily driven by chronic low-grade inflammation and metabolic imbalances, which reduce NO bioavailability and increase oxidative stress [20]. These interconnected me-chanisms contribute to the progression of cardiovascular complications and may exhibit sex-specific variations. ED in the aorta developed in male rats fed an HFD without an additive effect of Cmpd C, an AMPK inhibitor. MetS-related ED in male rats was prima-rily associated with downregulation of the AMPK/PI3K/eNOS signalling pathway, leading to reduced NO bioavailability and consequent impairment of endothelium-dependent vasorelaxation [21]. Inhibition of AMPK did not further exacerbate ED in the aorta. There is limited direct evidence that AMPK inhibition in the aorta affects PI3K/Akt/eNOS si-gnalling; instead, its primary effect appears to involve regulation of smooth muscle contractility via the RhoA/ROCK pathway, rather than NO signalling [22]. In contrast, ED was observed in both experimental groups in the mesenteric artery, with an evident addi-tive effect of AMPK inhibition. In mesenteric arteries, the downregulation of AMPK disrupts the AMPK/PI3K/eNOS signalling cascade, reducing eNOS phosphorylation and NO bioavailability, thereby contributing to ED and impaired vasodilation [23]. Additionally, the mesenteric artery relies more on endothelium-derived hyperpolarising factor (EDHF) than NO for vascular relaxation; thus, disruption of AMPK further impairs these compensatory mechanisms. Young females exhibited a protective effect against HFD-induced ED; however, this protection was abolished following AMPK inhibition, suggesting a newly identified female-specific role of AMPK in maintaining endothelial homeostasis and cardiovascular resilience in females.

Similar to the aorta, in the femoral artery of males, ED was induced by an HFD and combined with Cmpd C, with no additive effect. In females, however, the NO-dependent relaxation component in the femoral artery is significantly increased under an HFD, indicating the activation of compensatory mechanisms (possibly involving the upregulation of NO or alternative vasodilatory pathways) to preserve endothelial function. This suggests that the vascular system in females can adapt to metabolic stress by enhancing NO-mediated relaxation through an AMPK-dependent process. Due to sample size limitations, protein expression levels were not measured in the femoral and mesenteric arteries. However, future experiments could include these analyses to provide further mechanistic insight.

Interestingly, there is a clear difference between *Nos3* mRNA and protein expression. Our data indicate that mRNA expression of *Nos3* in aortic tissue in both males and females increased in HFD animals when AMPK was inhibited, as compared to HFD groups. In contrast, the protein expression was significantly reduced. It appears that reduced eNOS protein levels due to AMPK inhibition resulted in compensatory upregulation of *Nos3* mRNA in the aortas of rats on an HFD. Sozio et al. [24] showed that PPARα (peroxisome proliferator-activated receptor alpha) signalling is linked with AMPK [24] and can affect the expression of eNOS directly [25]. AMPK is known to inhibit the transcriptional activity of PPARα, and vice versa. AMPK activators, such as AICAR and metformin, decrease the basal and agonist-stimulated activities of PPARα, while inhibition of AMPK with Cmpd C activates PPARα. On the contrary, certain PPARα agonists activate AMPK in a receptor-independent manner. This regulation is essential for managing energy metabolism, as AMPK activation allows cells to prioritise short-term energy generation over long-term metabolic adaptations mediated by PPARα [24]. eNOS reduction at protein levels can be mediated by post-translational regulatory mechanisms. For instance, miR-155 and miR-195, which are induced by inflammatory stimuli, have been shown to decrease eNOS activity [18]. Furthermore, AMPK inhibition may interfere with the beneficial effects of miR-155 [26] or miR-195 [27]. Moreover, it is well established that eNOS activity is critically dependent on its dimerisation, which is highly susceptible to oxidative stress. Under oxidative conditions, eNOS monomers are more prone to ubiquitination and subsequent degradation [28]. Moreover, HFD may exacerbate this process by altering cellular conditions, favouring eNOS ubiquitination and degradation, further compromising endothelial function.

Oxidative stress-mediated ED are central to the pathophysiology of MetS, creating a bidirectional relationship that exacerbates the condition’s hallmark features of MetS, such as insulin resistance, hypertension, dyslipidemia, and obesity [29]. Excess reactive oxygen species (ROS) overwhelm the antioxidant defences, leading to oxidative stress [30]. ROS can directly damage endothelial cells by oxidising lipids, proteins, and DNA, which impairs their function. Additionally, ROS reduces the bioavailability of NO through NOS uncoupling and/or peroxynitrite formation and increases the activity of vasoconstrictors, disrupting vascular homeostasis [31]. In MetS, hyperglycemia and dyslipidemia activate NADPH oxidase (NOX) enzymes, particularly NOX2 and NOX4, in endothelial cells, adi-pocytes, and vascular smooth muscle cells [31]. Our data demonstrated increased ROS production in the aortic tissue of HFD-fed male rats, with a statistically significant, additive effect of AMPK inhibition. In contrast, female rats did not show elevated ROS formation if AMPK activity was preserved, suggesting a possible protective role of AMPK in reducing oxidative stress in females. This effect is attributed to the role of AMPK in maintaining redox homeostasis. AMPK plays a critical role in cellular energy balance, metabolism and oxidative stress, and is known for offering cardioprotection and antioxidant defence that benefits vascular health [6]. Its multifaceted role establishes it as a potential therapeutic target for diseases linked to oxidative stress. Interestingly, AMPK is also influenced by redox conditions, such as oxidative stress-mediated mitochondrial dysfunction, which results in alterations of the AMP:ATP and ADP:ATP ratios. This, in turn, activates AMPK through allosteric activation, promotes Thr172 phosphorylation, and/or inhibits Thr172 dephosphorylation [5,6]. Moreover, ROS can directly oxidise conserved cysteine residues on the AMPK catalytic α subunit and modify its conformation, contributing to its activation independently of changes in adenine nucleotide levels [32]. Activation of AMPK enhances antioxidant defences via AMPK-Nrf2 signalling cascade crosstalk [6]. Activated AMPK and Nrf2 collaborate at multiple levels to regulate the cellular stress response, even in distinct disease contexts. Likely, various processes controlled by AMPK occur simultaneously, either in collaboration or in opposition, to modulate Nrf2 activity in a context-specific manner [33]. Female rats exhibit more robust Nrf2-mediated antioxidant responses in the aorta compared to males, which are associated with estrogen-enhanced Nrf2 signalling and increased expression of downstream antioxidant enzymes, such as HO-1. This contributes to better oxidative stress resistance and endothelial function in females. In contrast, males generally show lower basal Nrf2 activity and higher oxidative stress in the aorta [34]. Our results indicated an impaired Nrf2-dependent antioxidant response in male HFD animals; however, in females, HFD alone did not result in these changes. Furthermore, our findings emphasise the significance of AMPK and the interaction between Nrf2 and AMPK in regulating antioxidant homeostasis during the development and progression of MetS. Importantly, we demonstrated the AMPK-specific role of sex in antioxidant protection.

Additionally, chronic inflammation associated with obesity triggers NOX activation by releasing pro-inflammatory cytokines (e.g., TNF-α, IL-6), which stimulate redox-sensitive signalling pathways [35]. This leads to an increased production of ROS, contributing to oxidative stress and vascular dysfunction. All these factors create a complex interaction that heightens cardiovascular risk. The oxidative stress-damaged endothelium is a source of pro-inflammatory signals, which boost the expression of adhesion molecules and cytokines, thereby recruiting immune cells to the vascular wall [36]. Moreover, macrophage infiltration and the release of pro-inflammatory cytokines (e.g., TNF-α, IL-6) exacerbate ED by enhancing ROS production and impairing endothelial function [37]. It has been demonstrated that chronic inflammation can reduce AMPK activity, creating a cycle that exacerbates metabolic issues. TNF-α can upregulate protein phosphatase 2C, inhibiting AMPK signalling and reducing AMPK phosphorylation and activity [38]. Reduced AMPK activity in MetS is associated with increased inflammation, insulin resistance, and metabolic dysregulation. This creates a vicious cycle where inflammation reduces AMPK acti-vity, which in turn exacerbates inflammation and metabolic disturbances [18]. Our data showed increased pro-inflammatory markers in aortic tissue due to HFD in male rats compared to females, who were protected against inflammation induced by HFD. It has been reported that females may be better protected against HFD-induced inflammation due to a preferential adipose tissue expansion in less inflammatory subcutaneous depots. In contrast, males tend to accumulate more visceral adipose tissue, particularly gonadal white adipose tissue, which exhibits greater macrophage infiltration and infla-mmatory responses during the development of obesity. Despite increased adiposity, females appear relatively protected from such inflammation in visceral fat depots. Additionally, females produce fewer pro-inflammatory lipid mediators and show increased resistance to accumulating pro-inflammatory immune cells [39]. Moreover, our data highlighted the role of AMPK during the development of MetS in female rats. AMPK has been shown to modulate inflammation during MetS by inhibiting pro-inflammatory pathways and promoting anti-inflammatory responses. Specifically, AMPK suppresses the nuclear factor-κB (NF-κB) signalling pathway, which is a key regulator of inflammation [40]. Furthermore, AMPK activation can decrease the levels of pro-inflammatory cytokines such as TNF-α and IL-6, which are elevated in MetS. This reduction in cytokine levels contributes to reduced inflammation and improved insulin sensitivity [40].

Mitochondrial dysfunction is another primary source of ROS in MetS. Mitochondria serve as sensors for environmental stressors, such as hyperglycemia. During mitochondrial respiration, electrons leak from the electron transport chain, particularly at Complexes I and III, forming superoxide anions through their interaction with molecular oxygen. Elevated levels of free fatty acids and glucose, characteristic of MetS, enhance mitochondrial activity, thereby increasing the probability of electron leakage and further amplifying ROS production. Additionally, reverse electron transfer at complex I, driven by a high proton motive force, increases ROS generation [41]. Additionally, impaired oxidative phosphorylation contributes to the primary characteristics of diabetes, including mitochondrial dysfunction and impaired glucose tolerance [42]. Triggered mitochondrial dysfunction can lead to apoptosis of endothelial cells, further compromising vascular integrity. AMPK plays a critical role in mitochondrial regulation by acting as an energy sensor that promotes mitochondrial biogenesis, dynamics, and mitophagy to maintain energy balance and cellular homeostasis [43].

Our data provide mechanistic insight into mitochondrial adaptation to metabolic stress. AMPK appears to play a central role in coordinating mitochondrial respiration in response to HFD, consistent with reports that nearly every mitochondrial insult triggers AMPK activation, thereby protecting mitochondrial integrity [44]. However, the persistence of antioxidant adaptations, such as elevated mitochondrial SOD activity despite AMPK inhibition, suggests the involvement of alternative regulatory pathways. This multifaceted response underscores the complexity and redundancy of mitochondrial defence mechanisms.

We observed substantial sex-dependent differences in MetS development, consistent with known sex-specific MetS. Women exhibit a higher prevalence of MetS (29% vs. 23% in men), driven by factors such as elevated body mass index, low HDL cholesterol, and central obesity. In contrast, men show higher rates of hypertension and hypertriglyceridemia [45]. These differences reflect variations in fat distribution and hormonal influences, particularly estrogen’s impact on vascular health. Estrogens appear to play a vital role in mediating the differences between males and females regarding their cardiovascular systems [46]. It is observed that postmenopausal women experience a significant decline in estrogen production from ovarian follicles, placing them at a higher risk of developing CVDs compared to men of the same age [46]. Our data emphasised the role of AMPK in the sex-dependent variations seen in MetS development. While AMPK inhibition did not yield an additive effect on HFD in males, it was crucial for MetS development in females, which could not be induced solely by the HFD diet. Therefore, we analysed sex hormone concentrations. Testosterone levels remained unchanged, while estradiol levels significantly decreased in females fed HFD and treated with AMPK inhibition. Given that MetS was induced in females only under reduced estradiol levels and decreased AMPK activity, our results reveal a critical dependency of endothelial homeostasis and cardiovascular resilience on the interplay between AMPK and estrogen signalling in females. Building on prior evidence that the interaction between AMPK and estrogen signalling is bidirectional, where estrogens activate AMPK and AMPK, which, in turn, modulate estrogen signalling [5], our findings further underscore the physiological significance of this crosstalk in metabolic disease contexts.

### Limitations of This Study

This study used only young animals, limiting the applicability of the results to older groups, where hormone levels and effects may differ due to ageing. This restricts generalizability and overlooks interactions between sex hormones and age-related changes. This study concentrates on the function of AMPK in the progression of ED within MetS. Nevertheless, the impact of AMPK on other organs, particularly the heart—considering cardiovascular complications—and its influence on the overall metabolism and bioenergetics of the organism, remains inadequately understood. Cmpd C, while widely used as an AMPK inhibitor, exhibits a broad and non-selective kinase inhibition profile. Besides AMPK, it has been reported to inhibit multiple other kinases, including VEGFR2, ALK2, AKT, and mTORC1/2, among others involved in cellular stress response pathways [47,48,49]. This lack of specificity complicates the interpretation of results, as the observed effects cannot be attributed solely to AMPK inhibition. Additionally, compound C has been shown to activate pathways such as the Calpain/Cathepsin pathway [47] and induce processes like autophagy [49], which may further complicate data interpretation. Therefore, off-target effects remain an essential consideration and potential limitation of this study. Future investigations should utilise more selective pharmacological inhibitors or complementary genetic approaches to target AMPK signalling and validate the current findings specifically.

## 5. Conclusions

In the present study, we demonstrated, for the first time, significant sex-dependent differences in the development and progression of MetS induced by an HFD, specifically in the context of AMPK-estrogen signalling crosstalk. Our findings provide novel insights into the critical, sex-specific role of AMPK in maintaining cardiovascular system integrity and vascular homeostasis, highlighting the essential interplay between AMPK and estrogen signalling in protecting against metabolic and vascular dysfunction. Our results revealed that MetS and associated cardiovascular comorbidities triggered by hyperglycemia and dyslipidemia in young male rats arise from complex interactions involving vascular oxidative stress, inflammation, mitochondrial dysfunction, and ED. MetS was induced only in young females when both AMPK and estrogen signalling were disrupted. An HFD alone did not lead to MetS in females due to the protective roles of AMPK and estrogen, conferring cardiovascular resilience (Graphical abstract). Our data suggest that targeting the AMPK-estrogen signalling pathway in females may provide an effective therapeutic strategy. This approach could particularly benefit patients at high cardiovascular risk, those with pre-existing cardiometabolic disease, or elderly pre-menopausal/menopausal women. Future prospective clinical trials are needed to confirm whether AMPK-estrogen-specific therapeutic strategies can effectively mitigate the adverse cardiovascular comorbidities associated with metabolic dysregulation.

## Data Availability

The original data presented in this study are openly available in the Supplementary Materials.

## Supplementary Materials

The following supporting information can be downloaded at: https://www.mdpi.com/article/10.3390/antiox14070843/s1, Figure S1: Treatment protocol; Figure S2: Induction of metabolic syndrome (MetS) in male and female rats—role of AMPK; Figure S3: Changes of maximal (Max.) Ach relaxation and EDCF-dependent contraction in mesenteric and femoral arteries; Figure S4: Changes of SOD activity and mitochondrial superoxide production in male and female rats—role of AMPK; Figure S5: Two-way ANOVA statistical analysis of the induction of endothelial dysfunction in the aorta results; Figure S6: Two-way ANOVA statistical analysis of the induction of endothelial dysfunction in mesenteric and femoral arteries; Figure S7: Two-way ANOVA statistical analysis of the induction of vascular oxidative stress results; Figure S8: Two-way ANOVA statistical analysis of the induction of vascular inflammation results; Figure S9: Two-way ANOVA statistical analysis of the induction of mitochondrial dysfunction I results; Figure S10: Two-way ANOVA statistical analysis of the induction of mitochondrial dysfunction II results; Figure S11: Two-way ANOVA statistical analysis of the expression of α1-AMPK and sex hormone levels during MetS development results; Figure S12: Two-way ANOVA statistical analysis of the induction of metabolic syndrome results; Figure S13: Two-way ANOVA statistical analysis of the induction of endothelial dysfuncton in *a. mesenterica* results; Figure S14: Two-way ANOVA statistical analysis of the induction of endothelial dysfunction in *a. femoralis*; Figure S15: Two-way ANOVA statistical analysis of the changes of SOD activity and mitochondrial superoxide production results; Figure S16: Four-parameter logistic (4PL) nonlinear regression analysis results of endothelial function; Figure S17: Corelation matrix of analysed results I; Figure S18: Corelation matrix of analysed results II; Table S1: Mean and SEM values for all measured parameters; Table S2: Two-way ANOVA statistical analysis results—Source of Variation; Table S3: Four-parameter logistic (4PL) nonlinear regression analysis results; Table S4: Kruskal-Wallis test analysis results—EC50 endothelial function; Table S5: Tukey-Kramer multiple comparisons—EC50 endothelial function.

## Abbreviations

The following abbreviations are used in this manuscript:

Ach	Acetylcholine
AMPK	Adenosine Monophosphate-Dependent Protein Kinase
ATP	Adenosine Triphosphate
AUC	Area Under the Curve
BMI	Body Mass Index
Bnip3	Bcl-2 Interacting Protein 3
BW	Body Weight
CaMKK2	Calcium/Calmodulin-Dependent Protein Kinase Kinase 2
Cmpd C	Compound C
COX2	Cyclooxygenase-2
CTR	Control
DMSO	Dimethyl Sulfoxide
CVDs	Cardiovascular Diseases
DHE	Dihydroethidium
ED	Endothelial Dysfunction
EDTA	Ethylenediaminetetraacetic Acid
EGTA	Ethylene Glycol Tetraacetic Acid
eNOS	Endothelial Nitric Oxide Synthase
ETC	Electron Transport Chain
FELASA	Federation of European Laboratory Animal Science Associations
FFA	Free Fatty Acids
GnRH	gonadotropin-releasing hormone
HDL	High-Density Lipoprotein
HFD	High-Fat Diet
HO-1	Heme oxygenase 1
HPLC	High-Performance Liquid Chromatography
IL10	Interleukin 10
Il1b	Interleukin 1-β
iNOS	Inducible Nitric Oxide Synthase
Kiss1	Kiss1 metastasis suppressor
LKB1	liver kinase B1
MetS	Metabolic Syndrome
NO	Nitric Oxide
NOS	Nitric Oxide Synthase
*Nos3*	Endothelial Nitric Oxide Synthase
NOX	NADPH Oxidase
NOX2	NADPH Oxidase 2
NOX4	NADPH Oxidase 4
Nrf2	Nuclear factor erythroid 2-related factor 2
OGTT	Oral Glucose Tolerance Test
PGC-1α	Peroxisome Proliferator-Activated Receptor Gamma Coactivator 1 Alpha
PMSF	Phenylmethylsulfonyl Fluoride
PPARα	Peroxisome Proliferator-Activated Receptor Alpha
p-α-AMPK	Alpha Adenosine Monophosphate-Dependent Protein Kinase Phosphorylated
qRT-PCR	Quantitative Real-Time Polymerase Chain Reaction
RET	Reverse Electron Transfer
ROS	Reactive Oxygen Species
rWAT/BW	Retroperitoneal White Adipose Tissue/Body Weight Ratio
sBP	Systolic Blood Pressure
SOD	Superoxide Dismutase
TAG	Triglycerides
TNFα	Tumour Necrosis Factor-α.
α1-AMPK	Alpha 1 Adenosine Monophosphate-Dependent Protein Kinase
