# Supplementary material for: Sex-Specific Cardiovascular Protection in Developing Metabolic Syndrome: The Role of AMPK
Source: Antioxidants (Basel). 2025 Jul 9;14(7):843. doi: 10.3390/antiox14070843 (PMC12291724; doi:10.3390/antiox14070843)
Supplement: Supplementary file 1 [file antioxidants-14-00843-s001.zip › antioxidants-3700677-supplementary.pdf]

Sex-Specific Cardiovascular Protection in Developing Metabolic Syndrome: The Role of AMPK Supplement

Miroslava Kvandova[1], Anna Zemancikova[1], Andrea Berenyiova[1], Iveta Waczulikova[2], Silvia Magyarova[1], Andrea Micurova[1], Jozef Torok[1], Marian Grman[3], Lenka Tomasova[3], Anton Misak[3], Zuzana Vysoka[1,4], Martina Manikova[1,4], Milan Zvarik[2], Patrick Mydla[2], Jana Vlkovicova[5], Peter Balis[1]* , Angelika Puzserova[1]*

[1] Institute of Normal and Pathological Physiology, Centre of Experimental Medicine, Slovak Academy of Sciences, Dúbravská cesta 9, 841 04 Bratislava, Slovakia; andrea.berenyiova@savba.sk (AB); andrea.micurova@savba.sk (AM); angelika.puzserova@savba.sk (AP); anna.zemancikova@savba.sk (AZ); jozef.torok@savba.sk (JT); miroslava.kvandova@savba.sk (MK); peter.balis@savba.sk (PB); silvia.magyarova@savba.sk (SM).

[2] Faculty of Mathematics, Physics and Informatics, Comenius University Bratislava, Mlynská dolina F1, 842 48 Bratislava, Slovakia; iveta.waczulikova@fmph.uniba.sk (IW); patrick.mydla@fmph.uniba.sk (PM); milan.zvarik@fmph.uniba.sk (MZ).

[3] Institute of Clinical and Translational Research, Biomedical Research Center, Slovak Academy of Sciences, Dúbravská cesta 9, 845 05, Bratislava, Slovakia; anton.misak@savba.sk (AMi); lenka.tomasova@savba.sk (LT); marian.grman@savba.sk (MG).

[4] Faculty of Chemical and Food Technology, Slovak University of Technology in Bratislava, Radlinského 9, 812 37 Bratislava, Slovakia; manikova.mata@gmail.com (MM); 1zuzana.vysoka@gmail.com (ZV).

[5] Institute for Heart Research, Centre of Experimental Medicine, Slovak Academy of Sciences, Dúbravská cesta 9, 841 04 Bratislava, Slovakia; jana.vlkovicova@savba.sk (JV).

Correspondence: miroslava.kvandova@savba.sk; Tel.: +4212/32296064; Institute of Normal and Pathological Physiology, Centre of Experimental Medicine, Slovak Academy of Sciences, Dúbravská cesta 9, 841 04 Bratislava, Slovakia

*These authors contributed equally to this work and are considered joined last authors

1. Methods

1.1. Animal model

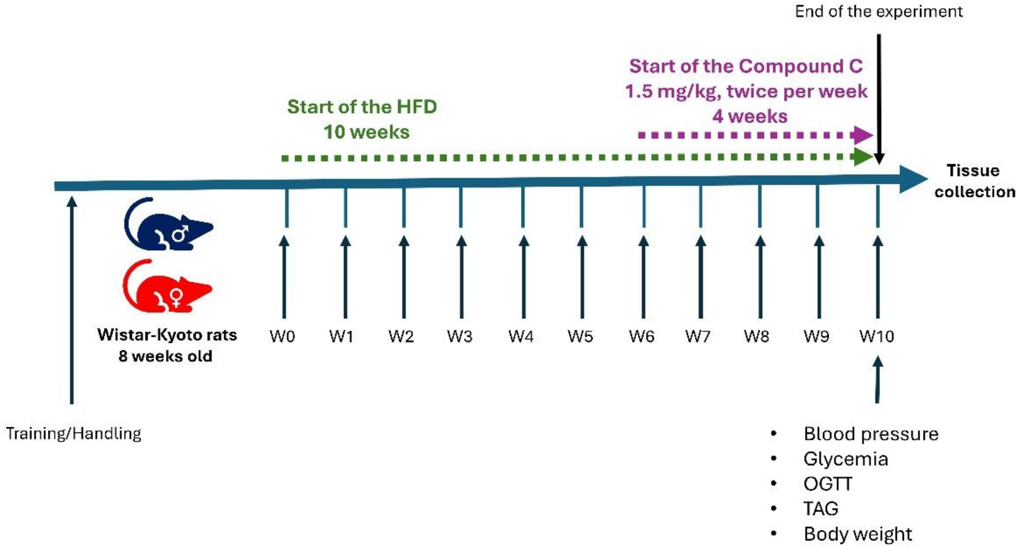

Supplementary Figure S1. Treatment protocol.

2. Results

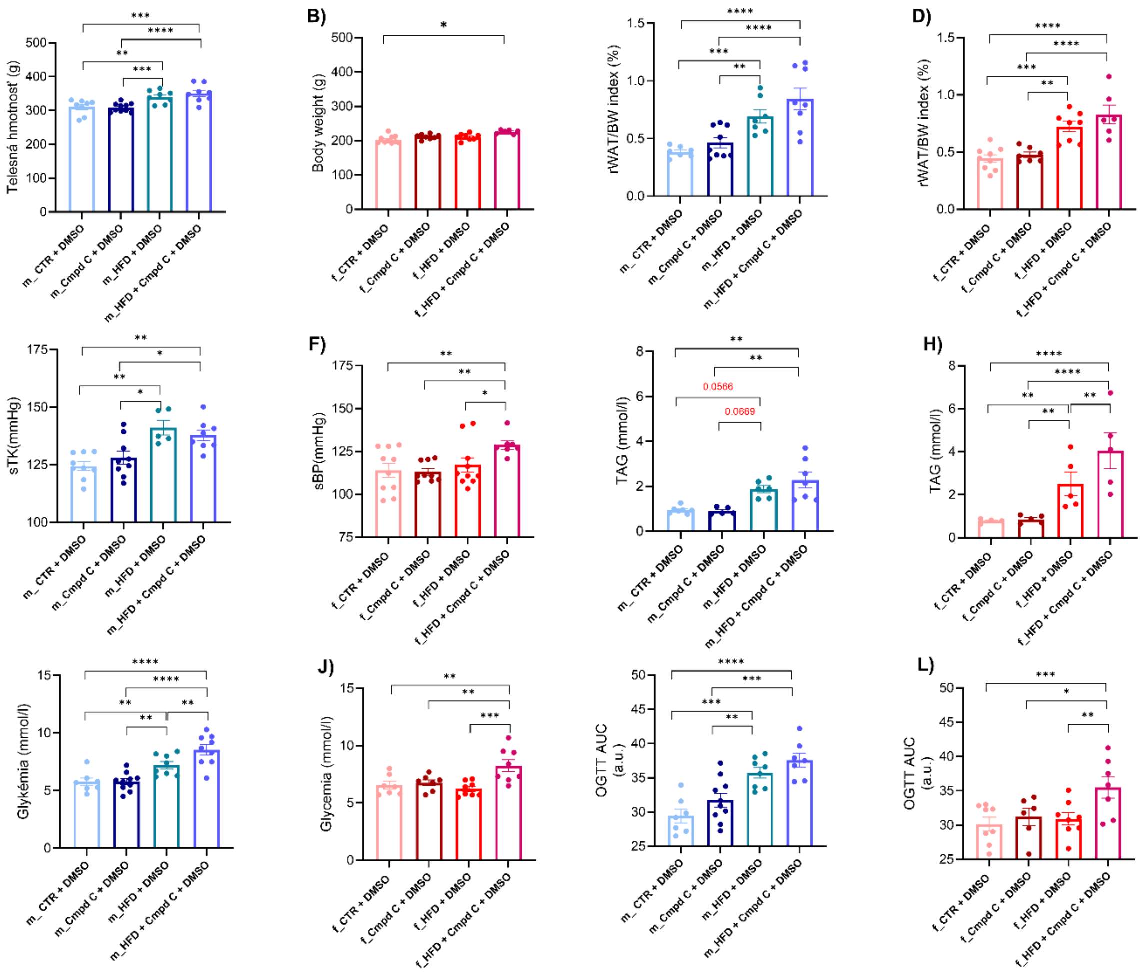

Supplementary Figure S2. Induction of metabolic syndrome (MetS) in male and female rats – role of AMPK. The development of MetS was [ssed using the following metabolic parameters: (A) body weight in male WKYs (n= 8 – 10); (B) body weight in female WKYs (n= 6 – 10); (C) retroperitoneal white adipose tissue/body weight ratio in male WKYs (n= 6 – 10); (D) retroperitoneal white adipose tissue/body weight ratio in female WKYs (n= 7 – 9); (E) systolic blood pressure in male WKYs (n= 5 – 9); (F) systolic blood pressure in female WKYs (n= 7 – 10);(G) fasting plasma triglycerides level in male WKYs (n= 5 – 7); (H) fasting plasma triglycerides level in female WKYs (n= 4 – 5); (I) fasting glycemia level in male WKYs (n= 7 – 10); (J) fasting glycemia level in female WKYs (n= 7 – 8); (K) insulin resistance determined by oral glucose tolerance test in male WKYs (n= 7 – 10); and (L) insulin resistance determined by oral glucose tolerance test in female WKYs (n= 6 – 8). Data are presented as mean ± SEM. P-values <0.05; were considered significant; * P ≤ 0.05, ** P ≤ 0.01, *** P ≤ 0.001, **** P ≤ 0.0001. *a.u.- arbitrary units; AUC- area under the curve; BW- body weight; Cmpd C- Compound C; CTR- control rats; HFD- high-fat diet; OGTT- oral glucose tolerance test; rWAT/BW- retroperitoneal adipose tissue/body weight ratio; sBP- systolic blood pressure; TAG- triglycerides.*

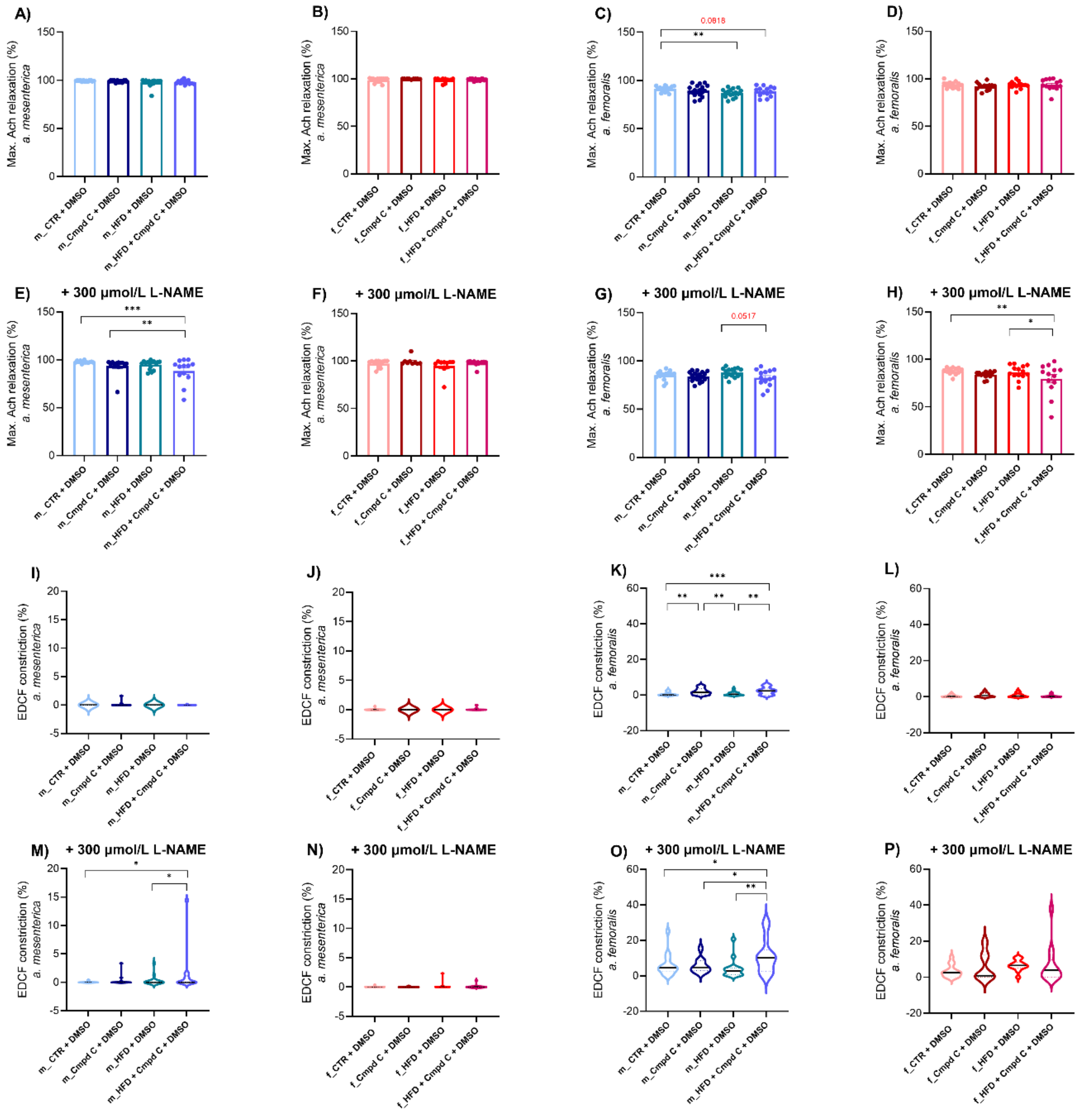

Supplementary Figure S3. Changes of maximal (Max.) Ach relaxation and EDCF-dependent contraction in mesenteric and femoral arteries. From Ach curves maximal value of relaxation was read for (A) Ach curve of young male WKYs (n= 12 – 16) and (B) Ach curve of young female WKY (n= 9 – 15) of *a. mesenterica*; and (C)

Ach curve of young male WKYs (n= 14 – 18) and (D) Ach curve of young female WKYs (n= 13 – 14) of *a. femoralis*; (E) Ach curve with 300 µmol/L L-NAME of young male WKYs (n= 12 – 16) and (F) Ach curve with 300 µmol/L L-NAME of young female WKY (n= 9 – 15) of *a. mesenterica*; and (G) Ach curve with 300 µmol/L L-NAME of young male WKYs (n= 14 – 18) and (H) Ach curve with 300 µmol/L L-NAME of young female WKYs (n= 13 – 14) of *a. femoralis*. From Ach curves EDCF- dependent contraction was calculated for (I) Ach curve of young male WKYs (n= 12 – 16) and (J) Ach curve of young female WKY (n= 9 – 15) of *a. mesenterica*; and (K) Ach curve of young male WKYs (n= 14 – 18) and (L) Ach curve of young female WKYs (n= 13 – 14) of *a. femoralis*; (M) Ach curve with 300 µmol/L L-NAME of young male WKYs (n= 12 – 16) and (N) Ach curve with 300 µmol/L L-NAME of young female WKY (n= 9 – 15) of *a. mesenterica*; and (O) Ach curve with 300 µmol/L L-NAME of young male WKYs (n= 14 – 18) and (P) Ach curve with 300 µmol/L L-NAME of young female WKYs (n= 13 – 14) of *a. femoralis*. Data are presented as mean ± SEM. P-values <0.05; were considered significant*;* * P ≤ 0.05, ** P ≤ 0.01, *** P ≤ 0.001, **** P ≤ 0.0001. *Cmpd C- Compound C; CTR- control rats; EDCF- endothelium-derived contracting factors; HFD- high-fat diet; L-NAME- N^G-nitro-L-arginine methyl ester.*

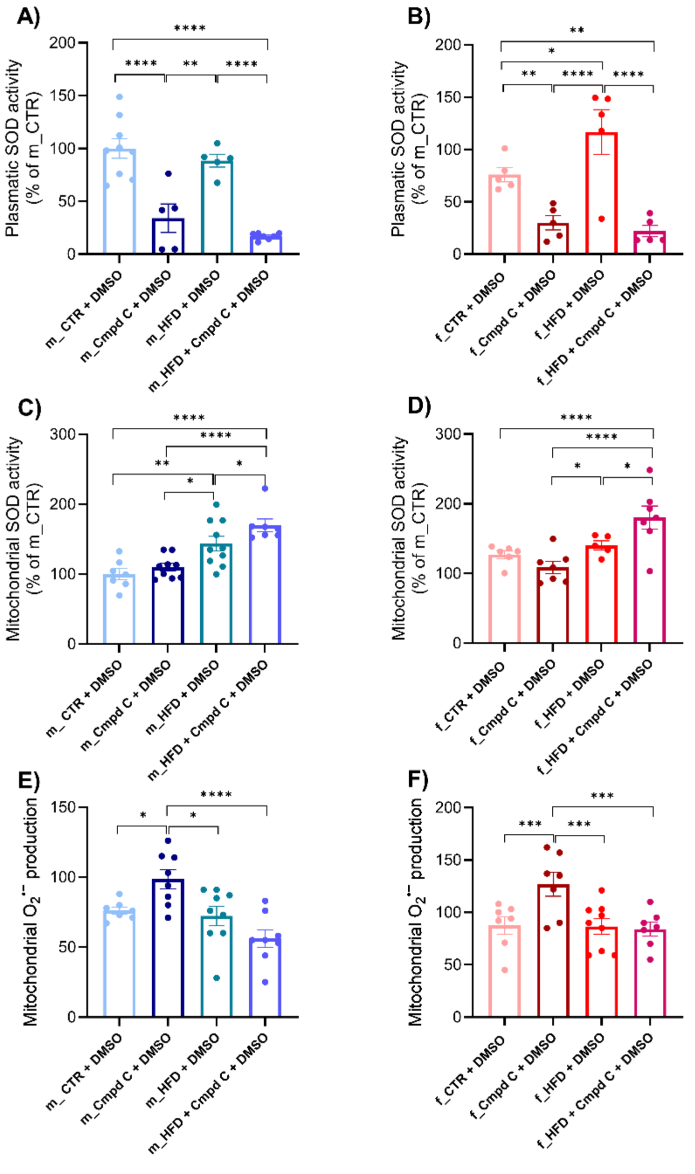

Supplementary Figure S4. Changes of SOD activity and mitochondrial superoxide production in male and female rats – role of AMPK. Total SOD activity was analysed in plasma samples of (A) young male WKYs

(n= 5 – 9) and (B) young female WKY(n= 5). Mitochondrial SOD activity was measured in (C) young male WKYs (n= 7 – 10) and (D) young female WKYs (n= 5 – 7). Mitochondrial specific production of superoxide determined by MitoSOX in (E) young male WKYs (n= 7 – 9) and (F) young female WKYs (n= 7 – 9). Data are presented as mean ± SEM. P-values <0.05; were considered significant*;* * P ≤ 0.05, ** P ≤ 0.01, *** P ≤ 0.001, **** P ≤ 0.0001. *Cmpd C- Compound C; CTR- control rats; HFD- high-fat diet; SOD- superoxide dismutase.*

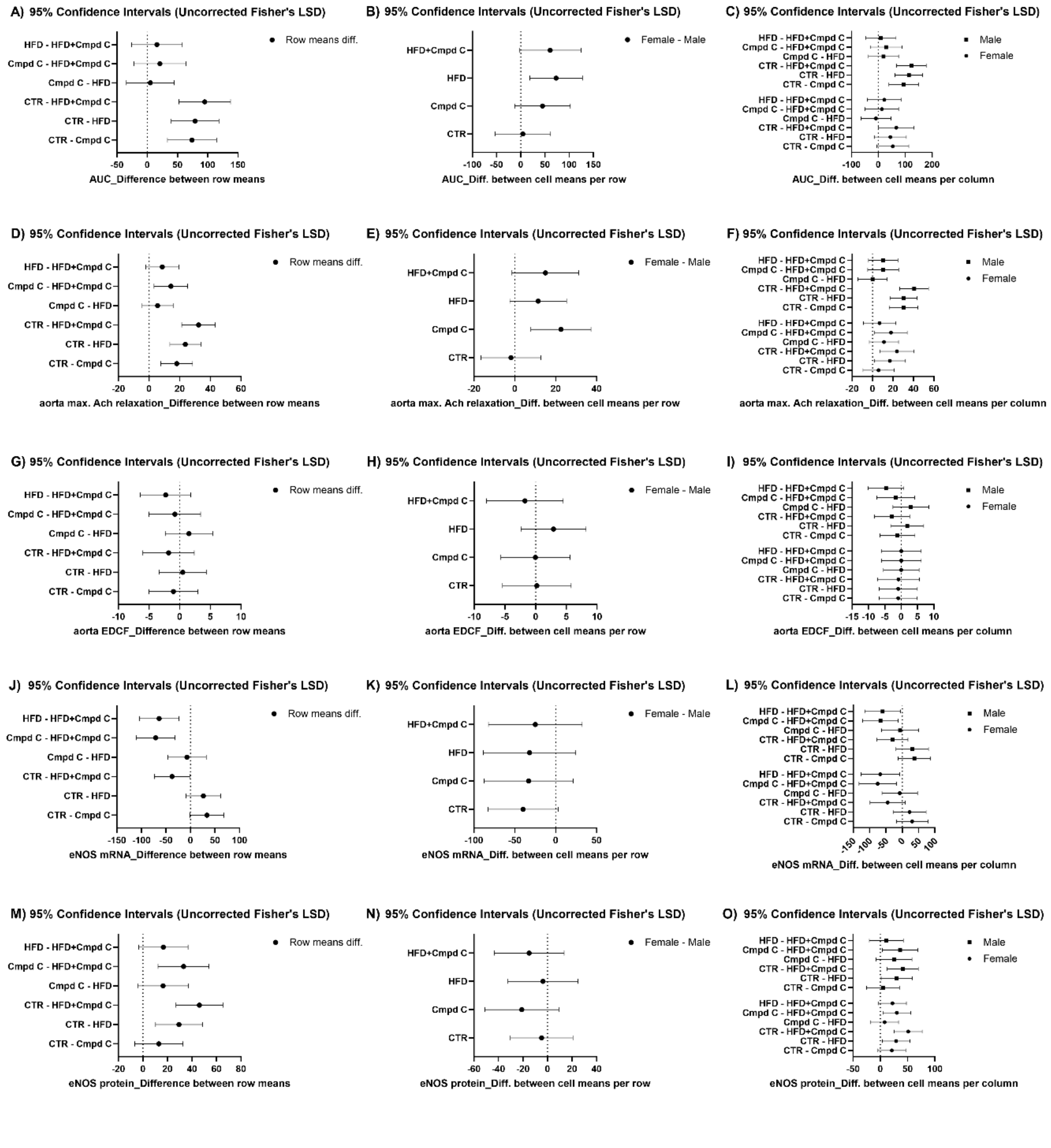

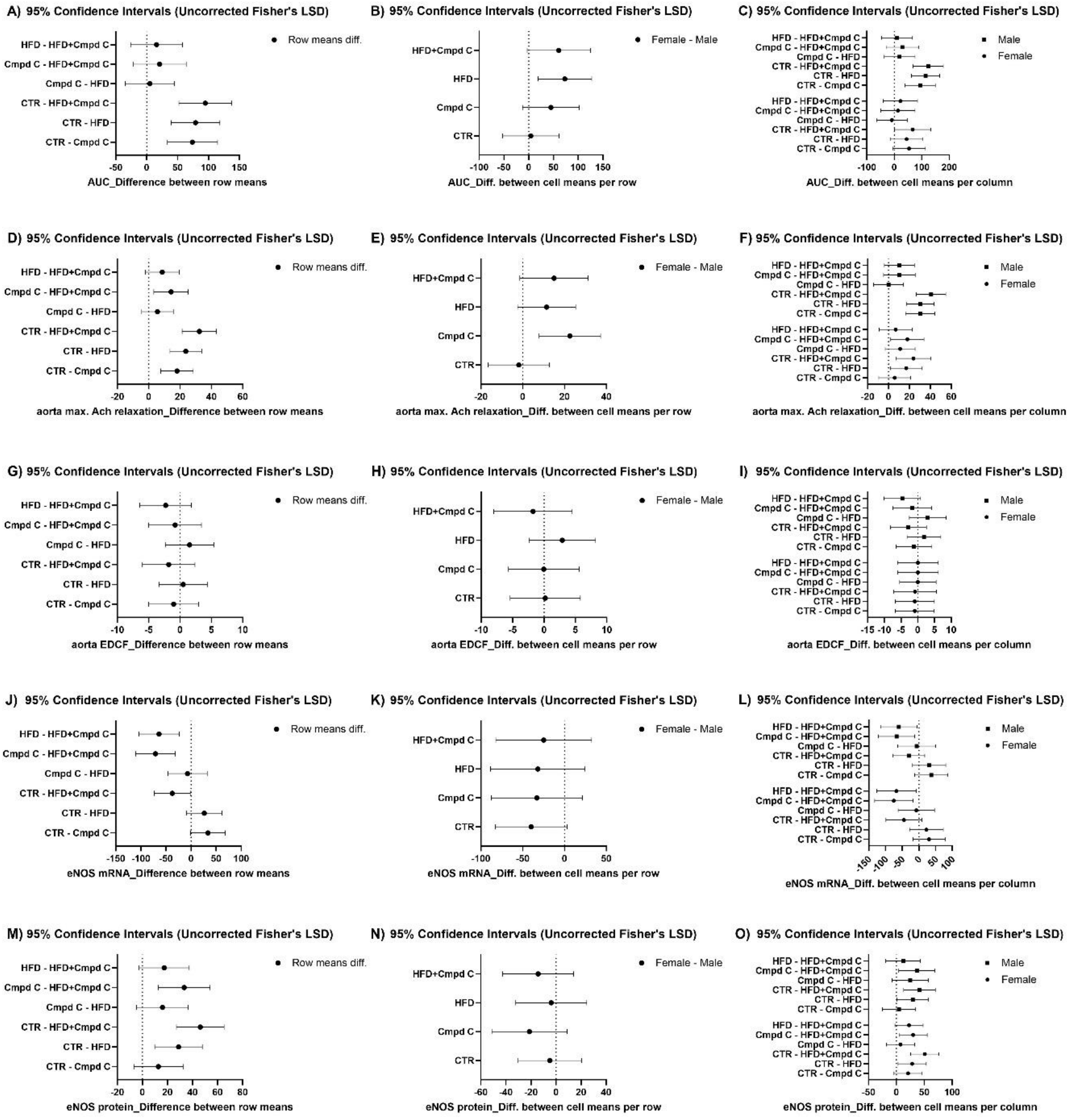

Supplementary Figure S5. Two-way ANOVA statistical analysis of the induction of endothelial dysfunction in the aorta results. (A) Differences between row means – AUC; (B) Differences between cell means per row – AUC; (C) Differences between cell means per column – AUC; (D) Differences between row means – max. Ach relaxation; (E) Differences between cell means per row – max. Ach relaxation; (F) Differences between cell means per column – max. Ach relaxation; (G) Differences between row means – EDCF contraction; (H) Differences between cell means per row – EDCF contraction; (I) Differences between cell means per column – EDCF contraction; (J) Differences between row means – *Nos3* mRNA expression; (K) Differences between cell means per row – *Nos3* mRNA expression; (L) Differences between cell means per column – *Nos3* mRNA expression; (M) Differences between row means – eNOS protein expression; (N) Differences between cell means per row –eNOS protein expression and (O) Differences between cell means per column – eNOS protein expression. *AUC- area under the curve; Cmpd C- Compound C; CTR- control rats; EDCF- Endothelium-derived contracting factors; eNOS- endothelial nitric oxide synthase; HFD- high-fat diet.*

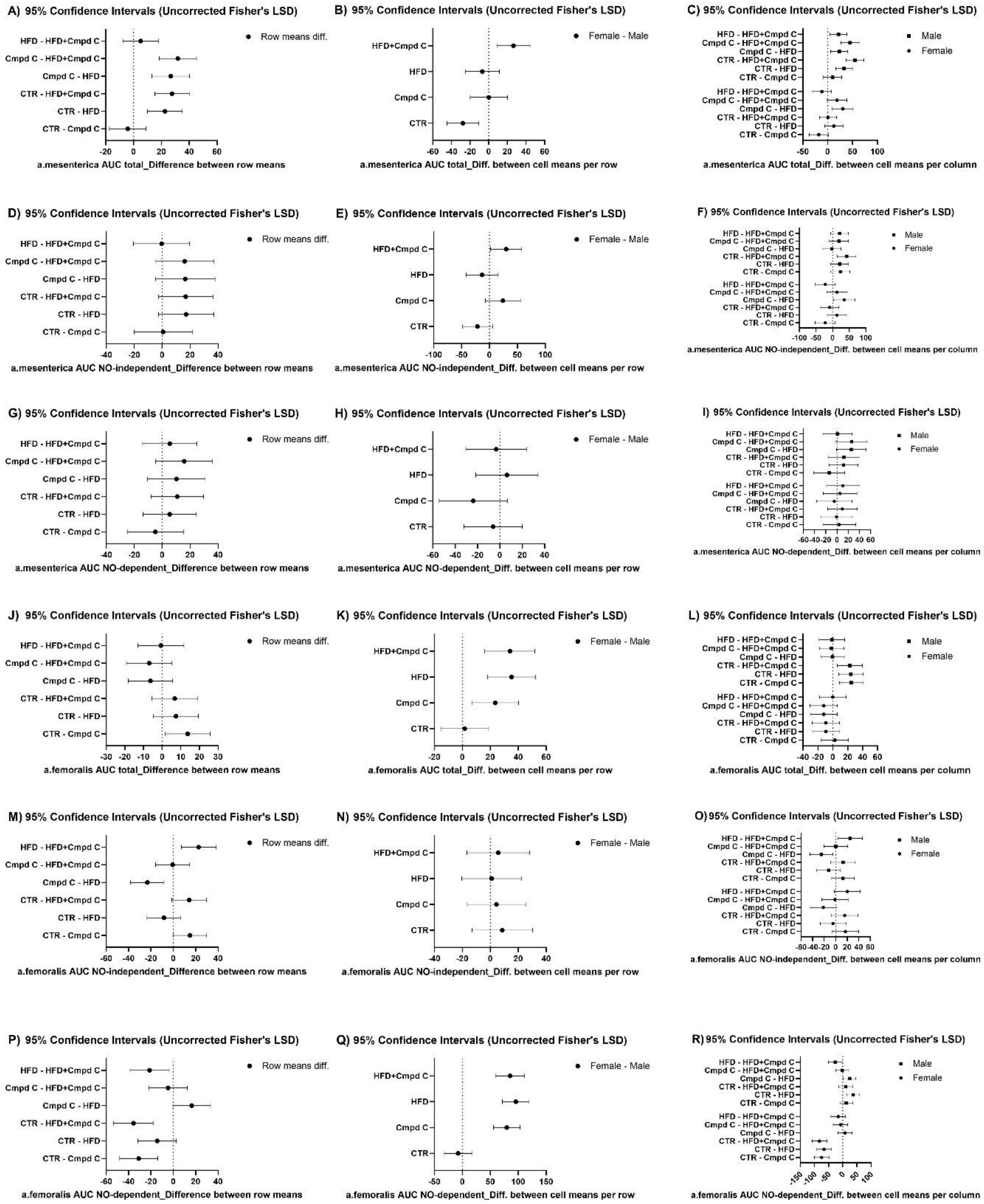

Supplementary Figure S6. Two-way ANOVA statistical analysis of the induction of endothelial dysfunction in mesenteric and femoral arteries. (A) Differences between row means – a. mesenterica AUC total; (B) Differences between cell means per row – a. mesenterica AUC total; (C) Differences between cell means per column – a. mesenterica AUC total; (D) Differences between row means – a. mesenterica AUC NO-independent; (E) Differences between cell means per row – a. mesenterica AUC NO-independent; (F) Differences between cell means per column – a. mesenterica AUC NO-independent; (G) Differences between row means – a. mesenterica AUC NO-dependent; (H) Differences between cell means per row – a. mesenterica AUC NO-dependent; (I) Differences between cell means per column – a. mesenterica AUC NO-dependent; (J) Differences between row means – a. femoralis AUC total; (K) Differences between cell means per row – a. femoralis AUC total; (L) Differences between cell means per column – a. femoralis AUC total; (M) Differences between row means – a.

femoralis AUC NO-independent; (N) Differences between cell means per row – a. femoralis AUC NO-independent; (O) Differences between cell means per column – a. femoralis AUC NO-independent; (P) Differences between row means – a. femoralis AUC NO-dependent; (Q) Differences between cell means per row – a. femoralis AUC NO-dependent and (R) Differences between cell means per column – a. femoralis AUC NO-dependent. *AUC- area under the curve; Cmpd C- Compound C; CTR- control rats; HFD- high-fat diet; NO- nitric oxide.*

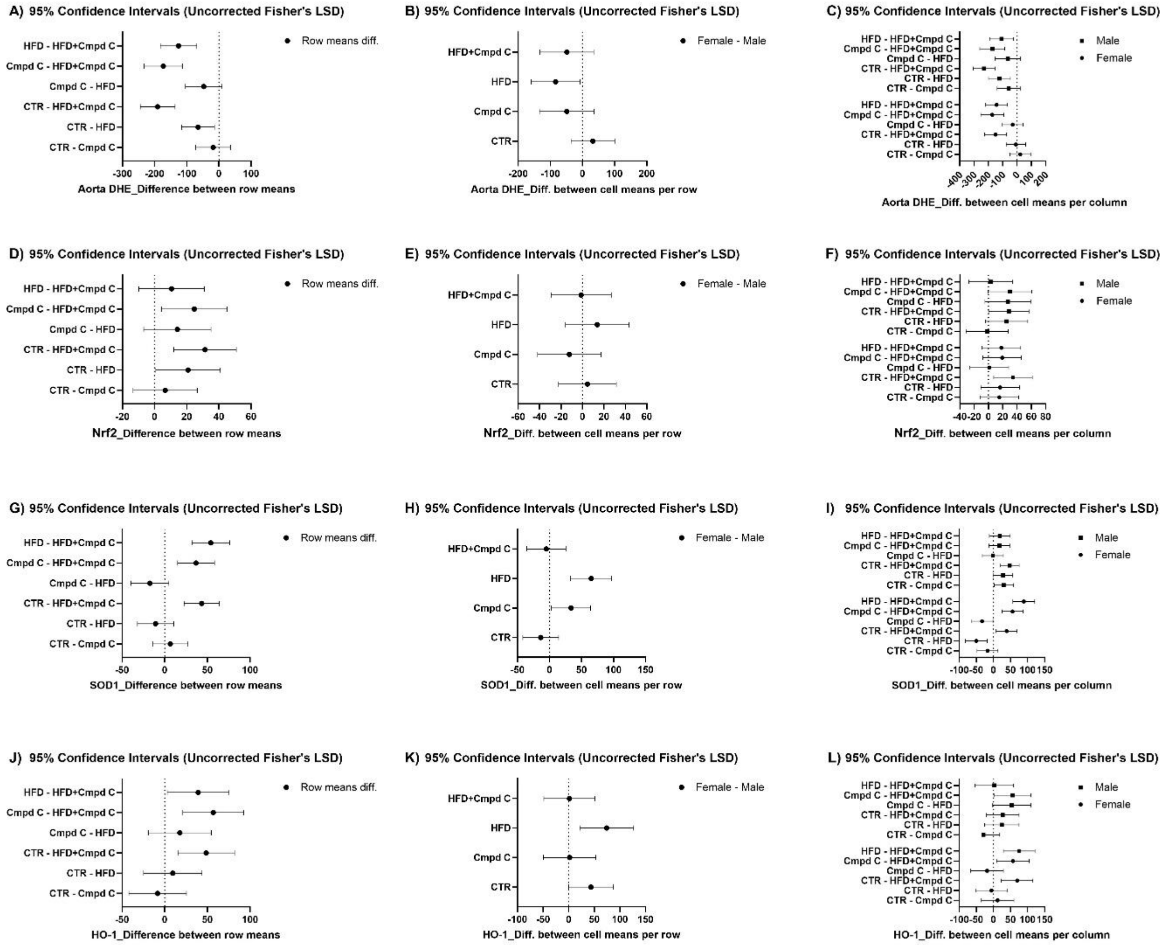

Supplementary Figure S7. Two-way ANOVA statistical analysis of the induction of vascular oxidative stress results. (A) Differences between row means – superoxide production; (B) Differences between cell means per row –superoxide production; (C) Differences between cell means per column – superoxide production; (D) Differences between row means – Nrf2 protein expression; (E) Differences between cell means per row – Nrf2 protein expression; (F) Differences between cell means per column – Nrf2 protein expression; (G) Differences between row means – SOD1 protein expression; (H) Differences between cell means per row – SOD1 protein expression; (I) Differences between cell means per column – SOD1 protein expression; (J) Differences between row means –HO-1 protein expression; (K) Differences between cell means per row – HO-1 protein expression and (L) Differences between cell means per column – HO-1 protein expression. *Cmpd C- Compound C; CTR- control rats; DHE- dihydroethidium; HFD- high-fat diet; HO-1- heme oxygenase 1; Nrf2- nuclear factor-like 2; SOD1- superoxide dismutase 1.*

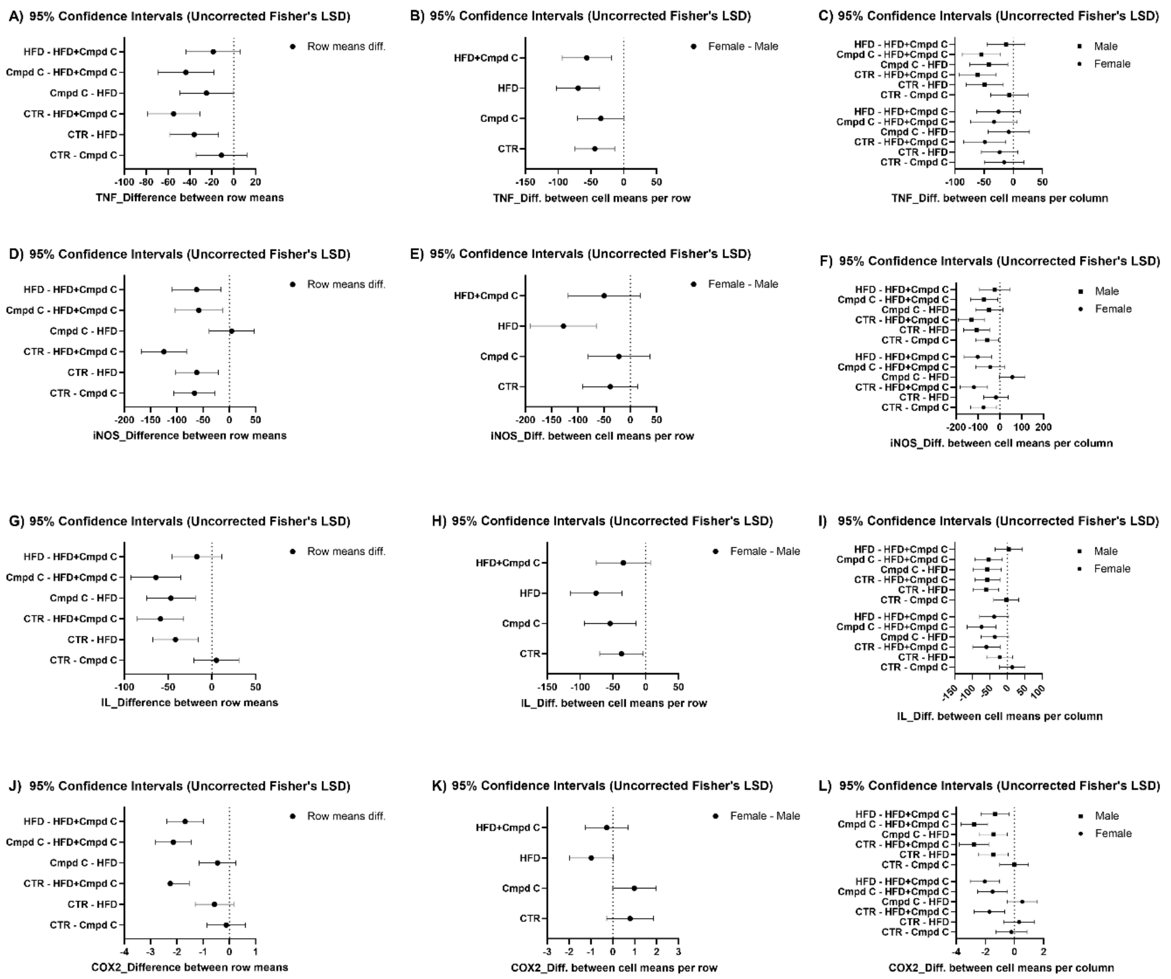

Supplementary Figure S8. Two-way ANOVA statistical analysis of the induction of vascular inflammation results. (A) Differences between row means – *Tnf*mRNA expression; (B) Differences between cell means per row – *Tnf* mRNA expression; (C) Differences between cell means per column – *Tnf* mRNA expression; (D) Differences between row means – *Inos* mRNA expression; (E) Differences between cell means per row – *Inos* mRNA expression; (F) Differences between cell means per column – *Inos* mRNA expression; (G) Differences between row means – *Il1b* mRNA expression; (H) Differences between cell means per row – *Il1b* mRNA expression; (I) Differences between cell means per column – *Il1b* mRNA expression; (J) Differences between row means – *Cox2* mRNA expression; (K) Differences between cell means per row – *Cox2* mRNA expression and (L) Differences between cell means per column – *Cox2* mRNA expression. *Cmpd C- Compound C; Cox2- cyclooxygenase-2; CTR- control rats; HFD- high-fat diet; Il1b- interleukin 1-β; Inos- inducible nitric oxide synthase; Tnf- tumour necrosis factor-alpha.*

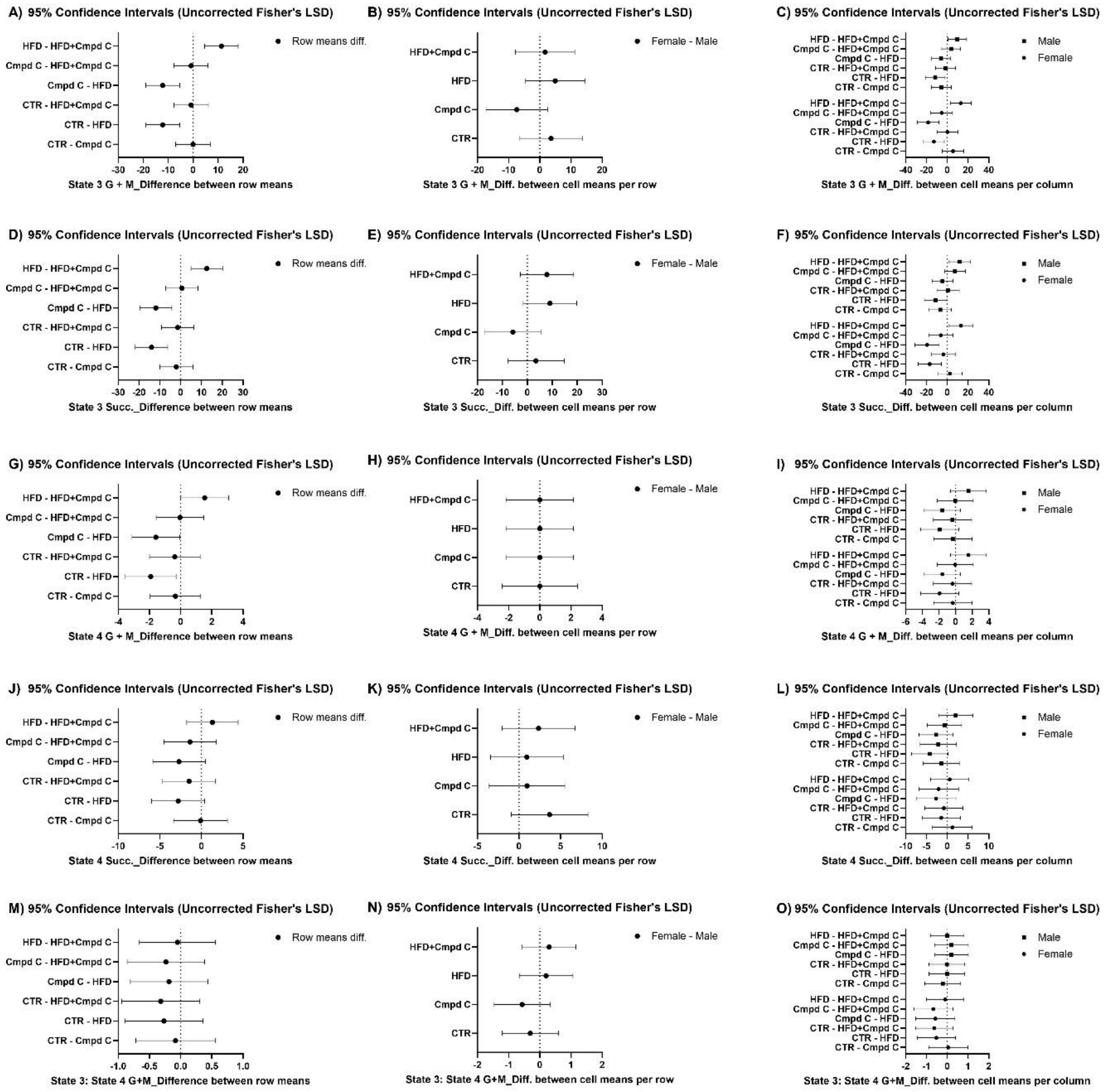

Supplementary Figure S9. Two-way ANOVA statistical analysis of the induction of mitochondrial dysfunction I results. (A) Differences between row means – State 3 G + M; (B) Differences between cell means per row – State 3 G + M; (C) Differences between cell means per column – State 3 G + M; (D) Differences between row means – State 3 Succ.; (E) Differences between cell means per row – State 3 Succ.; (F) Differences between cell means per column – State 3 Succ.; (G) Differences between row means – State 4 G + M; (H) Differences between cell means per row – State 4 G + M; (I) Differences between cell means per column – State 4 G + M; (J) Differences between row means – State 4 Succ.; (K) Differences between cell means per row – State 4 Succ.; (L) Differences between cell means per column – State 4 Succ.; (M) Differences between row means – State 3: State 4 G + M; (N) Differences between cell means per row – State 3: State 4 G + M and (O) Differences between cell means per column – State 3: State 4 G + M. *Cmpd C- Compound C; CTR- control rats; G- glutamate; HFD- high-fat diet; M- malate; Succ.- succinate.*

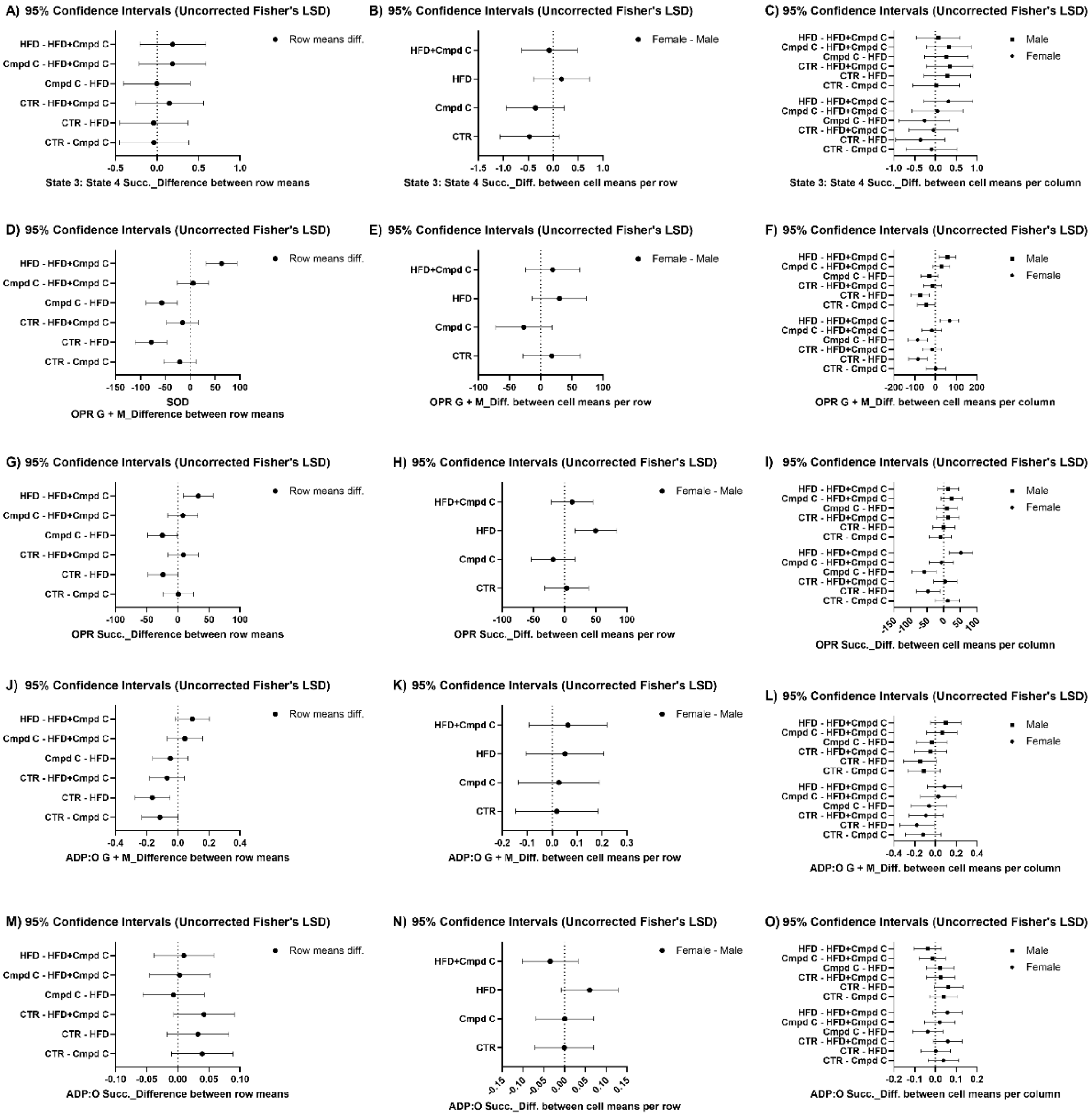

Supplementary Figure S10. Two-way ANOVA statistical analysis of the induction of mitochondrial dysfunction II results. (A) Differences between row means – State 3: State 4 Succ.; (B) Differences between cell means per row – State 3: State 4 Succ.; (C) Differences between cell means per column – State 3: State 4 Succ.; (D) Differences between row means – OPR G + M; (E) Differences between cell means per row – OPR G + M; (F) Differences between cell means per column – OPR G + M; (G) Differences between row means – OPR Succ.; (H) Differences between cell means per row – OPR Succ.; (I) Differences between cell means per column – OPR Succ.; (J) Differences between row means – ADP:O G + M; (K) Differences between cell means per row – ADP:O G + M; (L) Differences between cell means per column – ADP:O G + M; (M) Differences between row means – ADP:O Succ.; (N) Differences between cell means per row – ADP:O Succ. and (O) Differences between cell means per column – ADP:O Succ. *ADP- Adenosine-5'-diphosphate; Cmpd C- Compound C; CTR- control rats; G- glutamate; HFD- high-fat diet; M- malate; OPR- oxidative phosphorylation rate; O- one atom of oxygen; Succ.- succinate.*

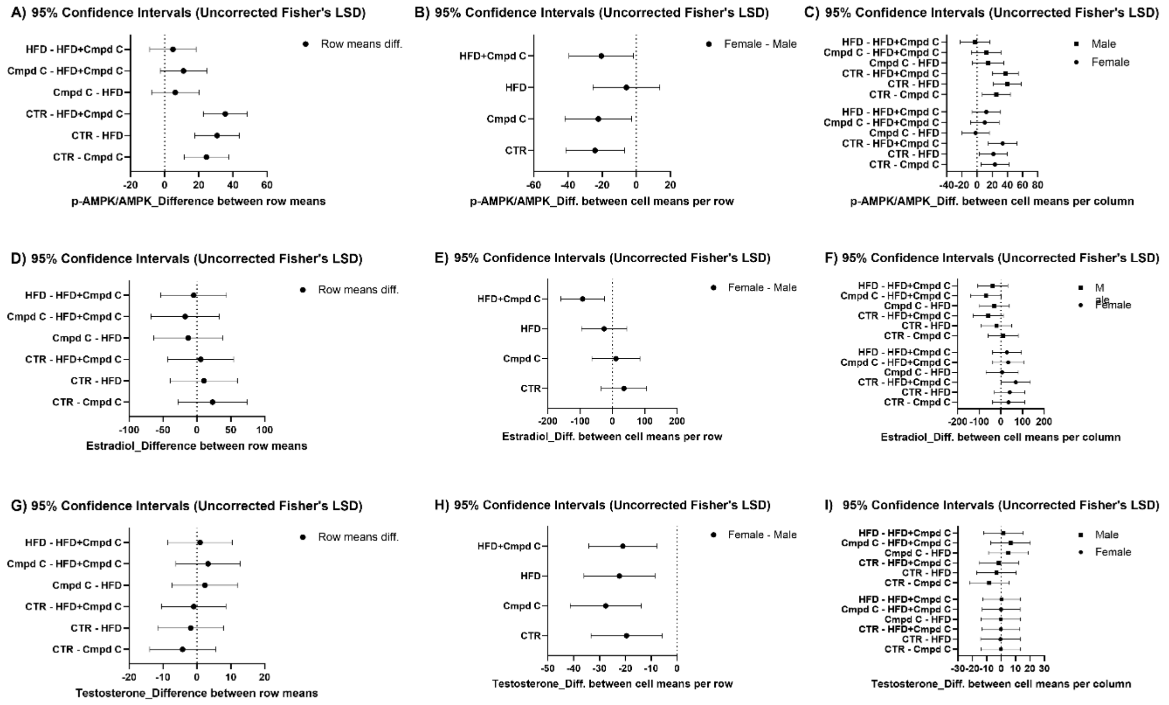

Supplementary Figure S11. Two-way ANOVA statistical analysis of the expression of α1-AMPK and sex hormone levels during MetS development results. (A) Differences between row means – p-α1-AMPK(Thr172)/α1-AMPK ratio; (B) Differences between cell means per row – p-α1-AMPK(Thr172)/α1-AMPK ratio; (C) Differences between cell means per column – p-α1-AMPK(Thr172)/α1-AMPK ratio; (D) Differences between row means – Estradiol level; (E) Differences between cell means per row – Estradiol level; (F) Differences between cell means per column – Estradiol level; (G) Differences between row means – Testosterone level; (H) Differences between cell means per row – Testosterone level and (I) Differences between cell means per column – Testosterone level. *α1-AMPK- alpha one adenosine monophosphate-dependent protein kinase; Cmpd C- Compound C; CTR- control rats; HFD- high-fat diet; p-α1-AMPK(Thr172)- alpha one adenosine monophosphate-dependent protein kinase phosphorylated at threonine 172.*

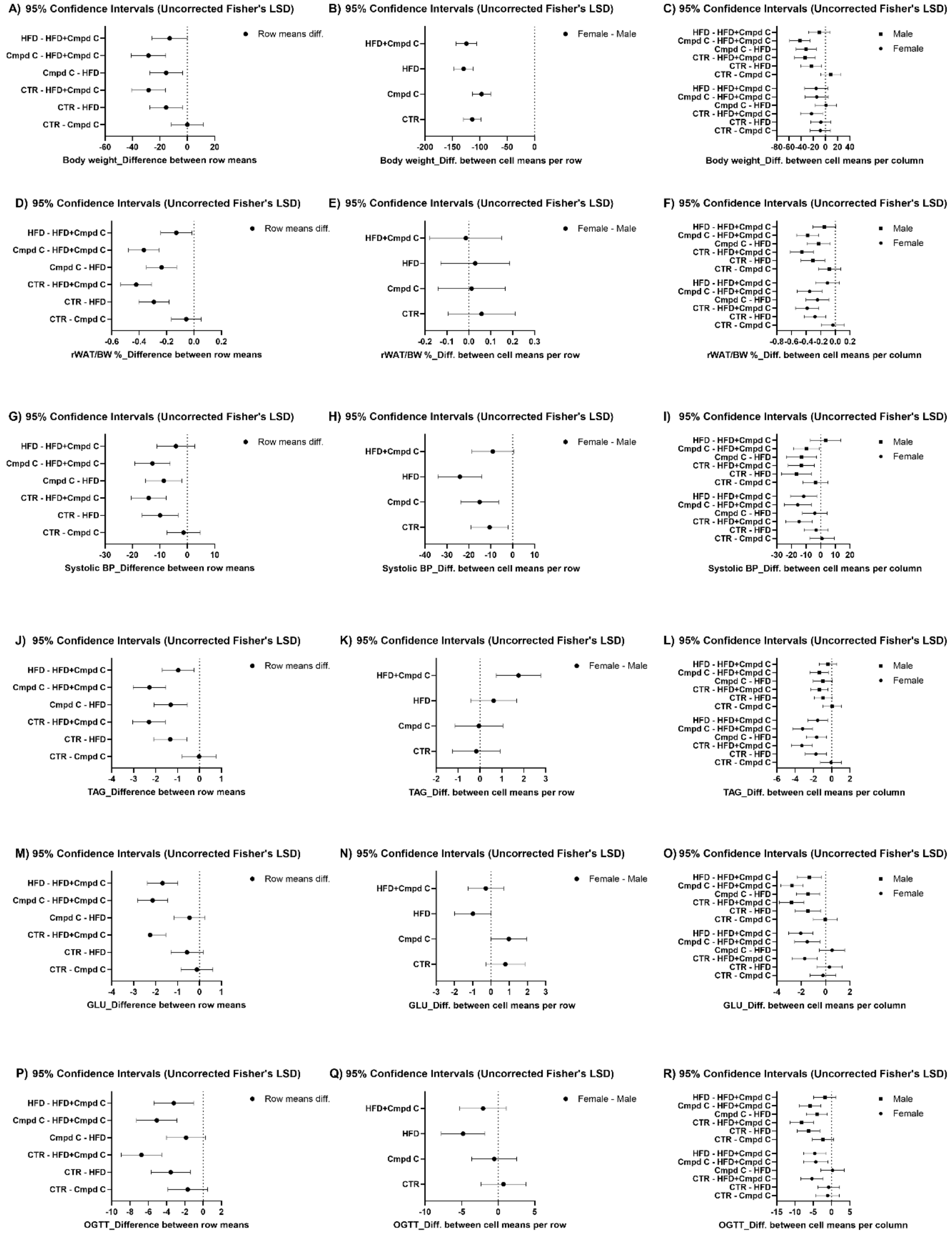

Supplementary Figure S12. Two-way ANOVA statistical analysis of the induction of metabolic syndrome results. (A) Differences between row means – BW; (B) Differences between cell means per row – BW; (C) Differences between cell means per column – BW; (D) Differences between row means – rWAT/BW ratio; (E) Differences between cell means per row – rWAT/BW ratio; (F) Differences between cell means per column – rWAT/BW ratio; (G) Differences between row means – sBP; (H) Differences between cell means per row – sBP; (I) Differences between cell means per column – sBP; (J) Differences between row means – TAG; (K) Differences between cell means per row – TAG; (L) Differences between cell means per column – TAG; (M) Differences between row

means – Glycemia; (N) Differences between cell means per row – Glycemia; (O) Differences between cell means per column – Glycemia; (P) Differences between row means – OGTT; (Q) Differences between cell means per row – OGTT and (R) Differences between cell means per column – OGTT. *BW- body weight; Cmpd C- Compound C; CTR- control rats; GLU- glucose level; HFD- high-fat diet; OGTT- oral glucose tolerance test; rWAT/BW- retroperitoneal white adipose tissue/body weight ratio; sBP- systolic blood pressure; TAG- triglycerides.*

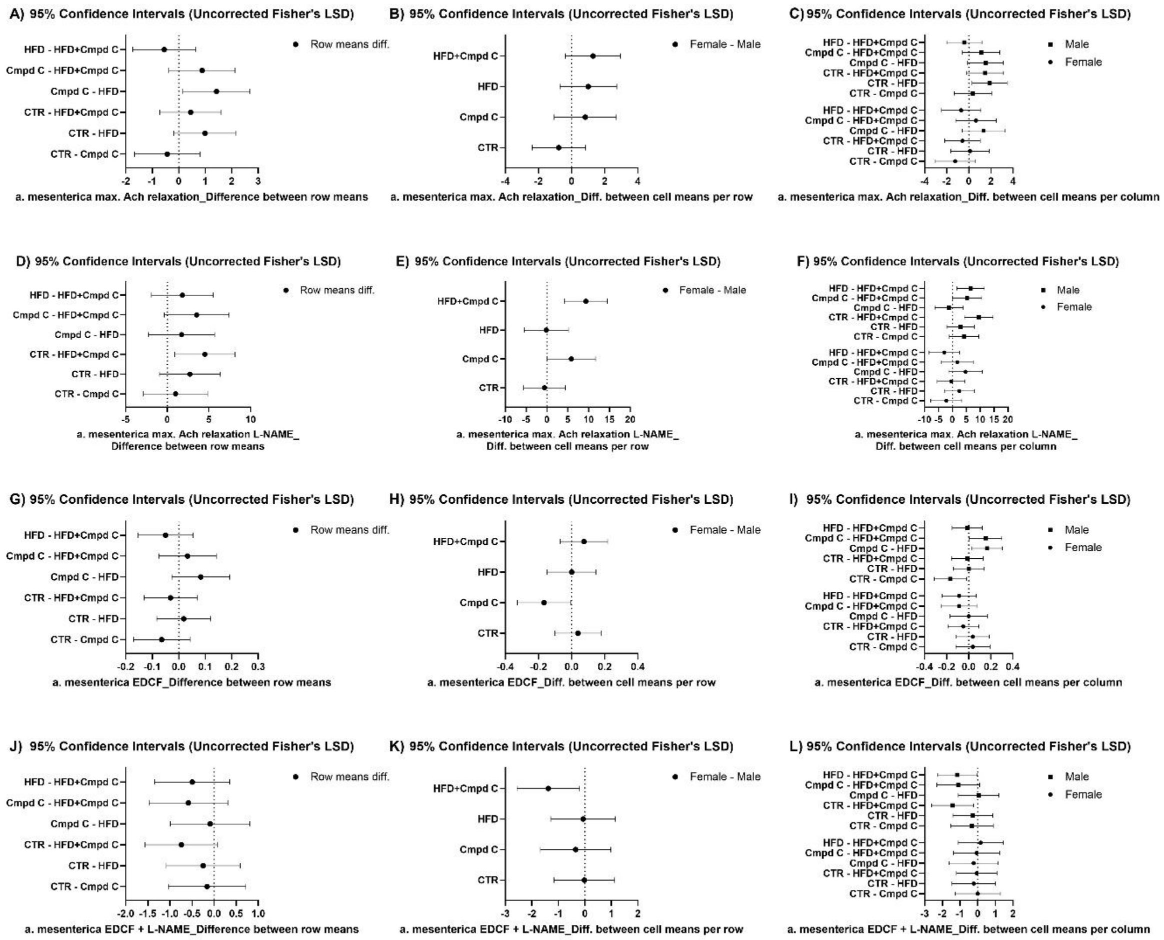

Supplementary Figure S13. Two-way ANOVA statistical analysis of the induction of endothelial dysfuncton in *a. mesenterica* results. (A) Differences between row means – *a. mesenterica* max. Ach relaxation; (B) Differences between cell means per row – *a. mesenterica* max. Ach relaxation; (C) Differences between cell means per column – *a. mesenterica* max. Ach relaxation; (D) Differences between row means – *a. mesenterica* max. Ach relaxation+ L-NAME; (E) Differences between cell means per row – *a. mesenterica* max. Ach relaxation + L-NAME; (F) Differences between cell means per column – *a. mesenterica* max. Ach relaxation + L-NAME; (G) Differences between row means – *a. mesenterica* EDCF; (H) Differences between cell means per row – *a. mesenterica* EDCF; (I) Differences between cell means per column – *a. mesenterica* EDCF; (J) Differences between row means – *a. mesenterica* EDCF + L-NAME; (K) Differences between cell means per row – *a. mesenterica* EDCF + L-NAME, and (L) Differences between cell means per column – *a. mesenterica* EDCF + L-NAME. *Cmpd C- Compound C; CTR- control rats; EDCF- endothelium-derived contracting factors; HFD- high-fat diet; L-NAME- N^G-nitro-L-arginine methyl ester.*

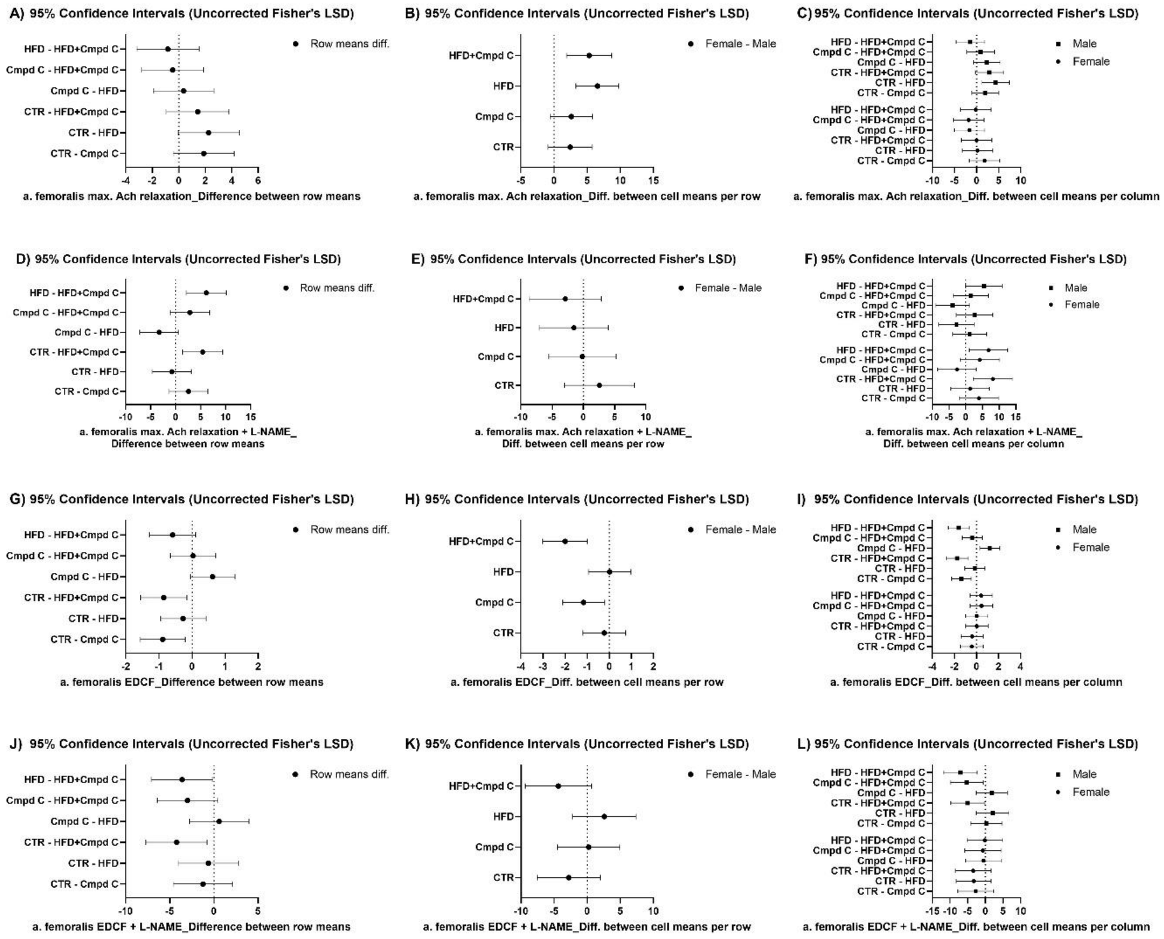

Supplementary Figure S14. Two-way ANOVA statistical analysis of the induction of endothelial dysfunction in *a. femoralis* results. (A) Differences between row means – *a. femoralis* max. Ach relaxation; (B) Differences between cell means per row – *a. femoralis* max. Ach relaxation; (C) Differences between cell means per column – *a. femoralis* max. Ach relaxation; (D) Differences between row means – *a. femoralis* max. Ach relaxation + L-NAME; (E) Differences between cell means per row – *a. femoralis* max. Ach relaxation + L-NAME; (F) Differences between cell means per column – *a. femoralis* max. Ach relaxation + L-NAME; (G) Differences between row means – *a. femoralis* EDCF; (H) Differences between cell means per row – *a. femoralis* EDCF; (I) Differences between cell means per column – *a. femoralis* EDCF; (J) Differences between row means – *a. femoralis* EDCF + L-NAME; (K) Differences between cell means per row – *a. femoralis* EDCF + L-NAME and (L) Differences between cell means per column – *a. femoralis* EDCF + L-NAME. *Cmpd C- Compound C; CTR-control rats; EDCF- endothelium-derived contracting factors; HFD- high-fat diet; L-NAME-* N^{G}*-nitro-L-arginine methyl ester.*

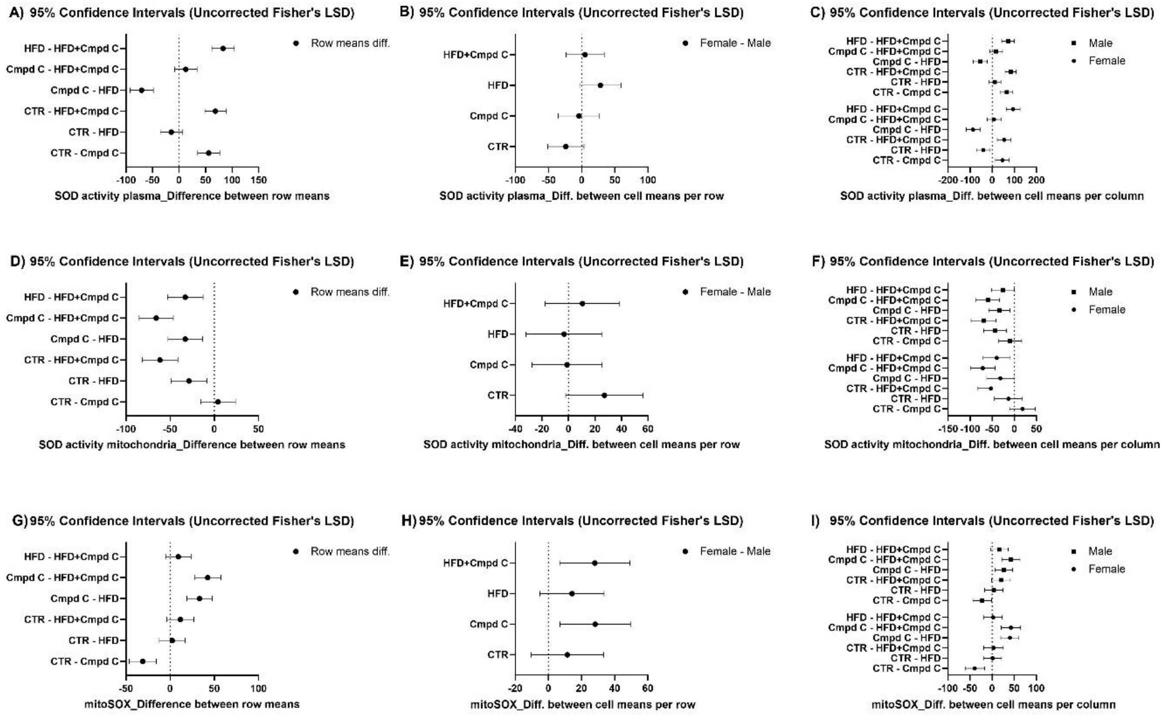

Supplementary Figure S15. Two-way ANOVA statistical analysis of the changes of SOD activity and mitochondrial superoxide production results. (A) Differences between row means – SOD activity plasma; (B) Differences between cell means per row – SOD activity plasma; (C) Differences between cell means per column – SOD activity plasma; (D) Differences between row means – SOD activity mitochondria; (E) Differences between cell means per row – SOD activity mitochondria; (F) Differences between cell means per column – SOD activity mitochondria; (G) Differences between row means – MitoSOX; (H) Differences between cell means per row – MitoSOX and (I) Differences between cell means per column – MitoSOX. *Cmpd C- Compound C; CTR- control rats; HFD- high-fat diet; SOD- superoxide dismutase.*

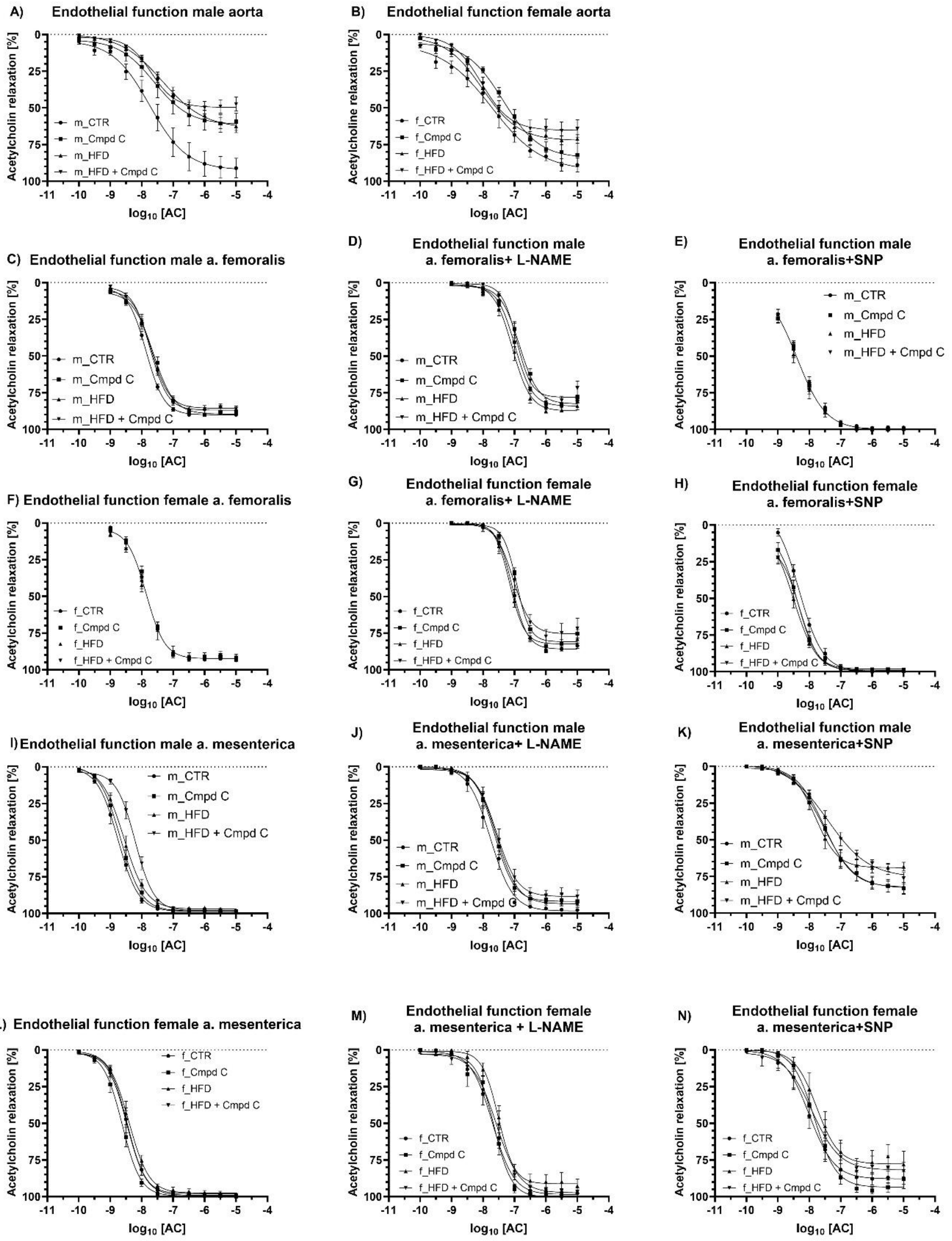

Supplementary Figure S16. Four-parameter logistic (4PL) nonlinear regression analysis results of endothelial function. (A) Endothellal function of male aorta; (B) Endothellal function of female aorta; (C) Endothellal function of male a. femoralis; (D) Endothellal function of male a. femoralis + 300 μM L-NAME; (E) Endothellal function of male a. femoralis – SNP; (F) Endothellal function of female a. femoralis; (G) Endothellal function of female a. femoralis + 300 μM L-NAME; (H) Endothellal function of female a. femoralis – SNP; (I) Endothellal function of male a. mesenterica; (J) Endothellal function of male a. mesenterica + 300 μM L-NAME; (K) Endothellal function of male a. mesenterica – SNP; (L) Endothellal function of female a. mesenterica; (M) Endothellal function of female a. mesenterica + 300 μM L-NAME; and (N) Endothellal function of female a. mesenterica – SNP. *Cmpd*

C- Compound C; CTR- control rats; HFD- high-fat diet; ; L-NAME- N^G-nitro-L-arginine methyl ester; SNP- Sodium nitroprusside.

All Groups Color map of correlations (Spreadsheet1) r>= -1 -0.80 -0.60 -0.40 -0.20 0 0.20 0.40 0.60 0.80 1

Variable	Sex M	Endothelial function AUC	Body weight	rWAT/BW %	Systolic blood pressure	TAG	Glucose	OGTT	SOD activity mitochondria	SOD activity plasma	Aorta superoxid DHE	eNOS mRNA expression	**TNF? mRNA expression**	**IL1ß mRNA expression**	COX2 mRNA expression	iNOSmRNA expression	Estradiol	Testosterone	p-AMPK/AMPK protein [illegible]	eNOS protein expression	Nrf2 protein expression	Catalase protein expression	SOD1 protein expression	HO-1 protein expression
Sex M	1,000	-0,280	0,941	-0,016	0,556	-0,220	-0,044	0,208	-0,119	0,018	0,169	0,342	0,581	0,514	-0,151	0,372	0,175	0,755	0,411	0,176	0,019	-0,158	-0,228	-0,161
Endothelial function AUC	-0,280	1,000	-0,675	-0,265	-0,390	-0,090	-0,229	-0,327	-0,106	0,138	-0,427	-0,373	-0,488	-0,504	-0,145	-0,503	0,210	-0,271	-0,055	0,105	0,096	-0,003	-0,006	0,010
Body weight	0,941	-0,675	1,000	0,214	0,643	-0,039	0,089	0,369	0,093	-0,092	0,266	0,457	0,674	0,697	0,105	0,532	0,084	0,763	0,303	0,065	-0,071	-0,251	-0,211	-0,168
rWAT/BW %	-0,016	-0,265	0,214	1,000	0,301	0,420	0,496	0,466	0,490	-0,205	0,477	0,415	0,441	0,451	0,413	0,383	-0,262	-0,341	-0,267	-0,464	-0,290	-0,093	-0,015	-0,154
Systolic blood pressure	0,556	-0,390	0,643	0,301	1,000	0,259	0,219	0,329	0,342	-0,221	0,431	0,314	0,435	0,487	0,146	0,314	0,031	0,518	0,144	-0,070	-0,062	-0,409	-0,481	-0,199
TAG	-0,220	-0,090	-0,039	0,420	0,259	1,000	0,568	0,548	0,564	-0,163	0,637	0,448	0,561	0,873	0,911	0,701	0,107	-0,237	-0,128	-0,220	-0,224	-0,520	-0,299	-0,481
Glucose	-0,044	-0,229	0,089	0,496	0,219	0,568	1,000	0,484	0,617	-0,320	0,481	0,448	0,277	0,264	0,506	0,448	0,122	-0,161	-0,219	-0,311	-0,096	-0,510	-0,297	-0,332
OGTT	0,208	-0,327	0,369	0,466	0,329	0,548	0,484	1,000	0,278	-0,353	0,403	0,370	0,535	0,602	0,292	0,538	0,331	0,054	-0,153	-0,395	-0,135	-0,341	-0,286	-0,394
SOD activity mitochondria	-0,119	-0,106	0,093	0,490	0,342	0,564	0,617	0,278	1,000	-0,240	0,414	0,231	0,179	0,133	0,555	-0,047	-0,212	-0,008	-0,162	-0,289	-0,174	-0,445	-0,471	-0,160
SOD activity plasma	0,018	0,138	-0,092	-0,205	-0,221	-0,163	-0,320	-0,353	-0,240	1,000	-0,233	-0,063	-0,017	0,051	-0,211	-0,422	-0,236	-0,303	0,301	0,064	0,160	0,348	0,429	-0,073
Aorta superoxid DHE	0,169	-0,427	0,266	0,477	0,431	0,637	0,481	0,403	0,414	-0,233	1,000	0,579	0,559	0,490	0,594	0,578	0,012	-0,037	0,114	-0,364	-0,218	-0,341	-0,385	-0,331
eNOS mRNA expression	0,342	-0,373	0,457	0,415	0,314	0,448	0,448	0,370	0,231	-0,063	0,579	1,000	0,406	0,467	0,306	0,502	0,010	0,023	-0,085	-0,035	-0,031	-0,217	0,058	0,031
**TNF? mRNA expression**	0,581	-0,488	0,674	0,441	0,435	0,561	0,277	0,535	0,179	-0,017	0,559	0,406	1,000	0,687	0,569	0,777	-0,109	0,127	0,098	-0,172	-0,230	-0,310	-0,206	-0,255
**IL1ß mRNA expression**	0,514	-0,504	0,697	0,451	0,487	0,873	0,264	0,602	0,133	0,051	0,490	0,467	0,687	1,000	0,451	0,696	-0,126	0,374	-0,085	-0,137	-0,245	-0,031	-0,016	0,062
COX2 mRNA expression	-0,151	-0,145	0,105	0,413	0,146	0,911	0,506	0,292	0,555	-0,211	0,594	0,306	0,569	0,451	1,000	0,668	-0,619	-0,261	-0,250	-0,290	-0,026	-0,661	-0,292	-0,352
iNOSmRNA expression	0,372	-0,503	0,532	0,383	0,314	0,701	0,448	0,538	-0,047	-0,422	0,578	0,502	0,777	0,696	0,668	1,000	-0,439	0,182	-0,197	-0,090	-0,245	-0,259	0,144	0,085
Estradiol	0,175	0,210	0,084	-0,262	0,031	0,107	0,122	0,331	-0,212	-0,236	0,012	0,010	-0,109	-0,126	-0,619	-0,439	1,000	0,137	0,024	0,122	0,144	-0,058	-0,104	-0,082
Testosterone	0,755	-0,271	0,763	-0,341	0,518	-0,237	-0,161	0,054	-0,008	-0,303	-0,037	0,023	0,127	0,374	-0,261	0,182	0,137	1,000	0,302	0,130	0,104	-0,185	-0,145	0,018
p-AMPK/AMPK protein expression	0,411	-0,055	0,303	-0,267	0,144	-0,128	-0,219	-0,153	-0,162	0,301	0,114	-0,085	0,098	-0,085	-0,250	-0,197	0,024	0,302	1,000	0,344	0,167	0,222	-0,207	-0,145
eNOS protein expression	0,176	0,105	0,065	-0,464	-0,070	-0,220	-0,311	-0,395	-0,289	0,064	-0,364	-0,035	-0,172	-0,137	-0,290	-0,090	0,122	0,130	0,344	1,000	0,392	0,130	-0,071	0,215
Nrf2 protein expression	0,019	0,096	-0,071	-0,290	-0,062	-0,224	-0,096	-0,135	-0,174	0,160	-0,218	-0,031	-0,230	-0,245	-0,026	-0,245	0,144	0,104	0,167	0,392	1,000	0,138	0,172	-0,024
Catalase protein expression	-0,158	-0,003	-0,251	-0,093	-0,409	-0,520	-0,510	-0,341	-0,445	0,348	-0,341	-0,217	-0,310	-0,031	-0,661	-0,259	-0,058	-0,185	0,222	0,130	0,138	1,000	0,441	0,373
SOD1 protein expression	-0,228	-0,006	-0,211	-0,015	-0,481	-0,299	-0,297	-0,286	-0,471	0,429	-0,385	0,058	-0,206	-0,016	-0,292	0,144	-0,104	-0,145	-0,207	-0,071	0,172	0,441	1,000	0,601
HO-1 protein expression	-0,161	0,010	-0,168	-0,154	-0,199	-0,481	-0,332	-0,394	-0,160	-0,073	-0,331	0,031	-0,255	0,062	-0,352	0,085	-0,082	0,018	-0,145	0,215	-0,024	0,373	0,601	1,000

Supplementary Figure S17. Corelation matrix of analysed results.

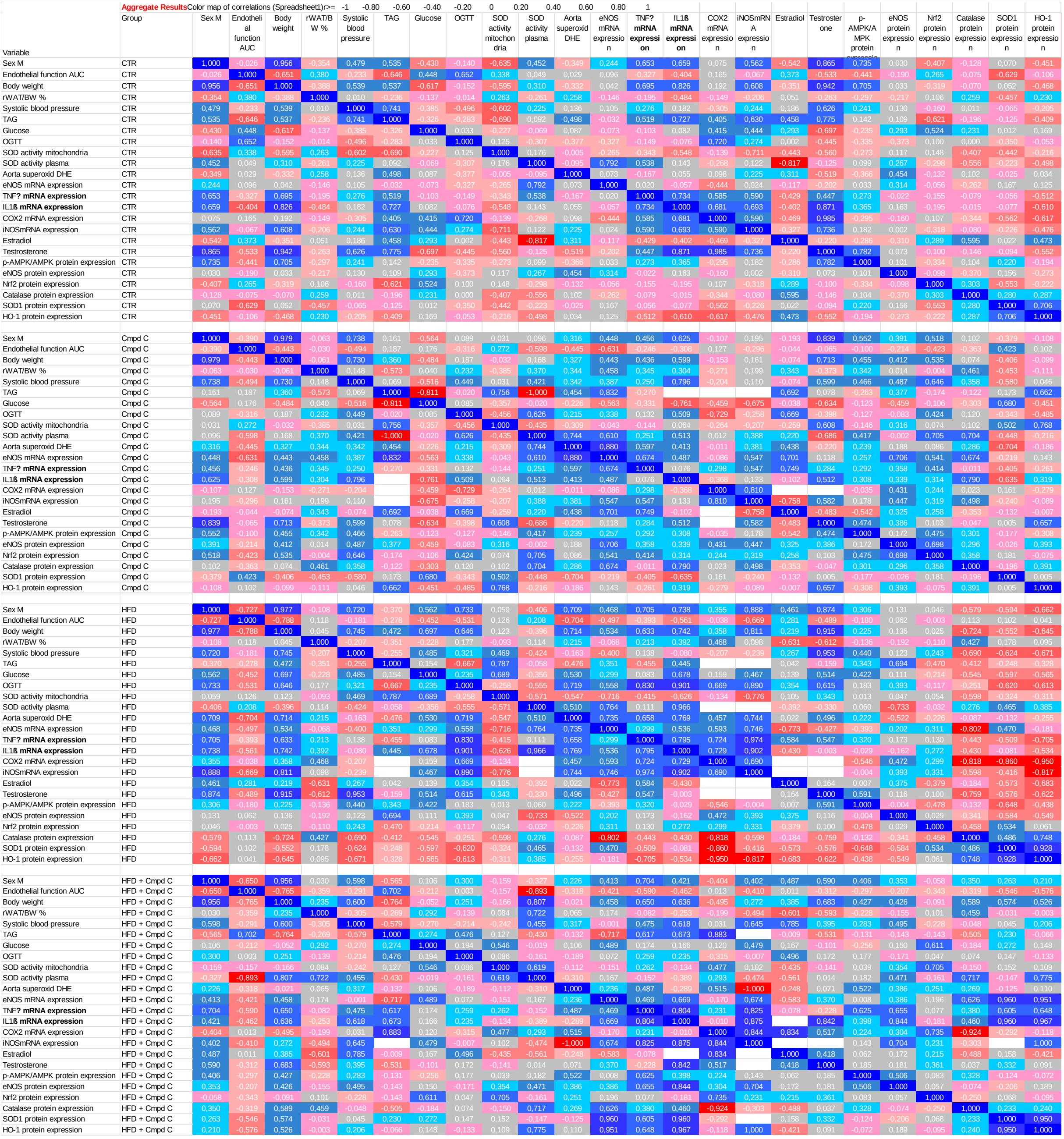

Supplementary Figure S18. Corelation matrix of analysed results.

Supplementary Table S1. Mean and SEM values for all measured parameters.

		m_CTR	m_Cmpd C	m_HFD	m_HFD + Cmpd C	f_CTR	f_Cmpd C	f_HFD	f_HFD + Cmpd C
**Body weight**	mean	310,3000	308,5000	340,3000	350,6000	203,3000	211,8000	210,9000	226,2000
	SEM	8,0330	4,0500	6,6490	9,3330	3,5900	2,5480	3,4090	2,2720
**rWAT/BW %**	mean	0,3857	0,4648	0,6932	0,8436	0,4440	0,4776	0,7223	0,8299
	SEM	0,0178	0,0441	0,0595	0,0939	0,0333	0,0230	0,0458	0,0823
**Systolic blood pressure**	mean	1,8770	2,8530	3,2030	2,3410	114,0000	113,1000	117,1000	128,8000

	SEM	1,8770	2,8530	3,2030	2,3410	4,0840	1,9230	4,2060	2,3980
**TAG**	mean	0,9471	0,9060	1,8820	2,2860	0,7775	0,8560	2,5040	4,0400
	SEM	0,0594	0,0700	0,1550	0,3496	0,0533	0,0826	0,5447	0,8357
**Glycemia**	mean	5,7570	5,7800	7,2130	8,5440	6,5430	6,7570	6,2250	8,2630
	SEM	0,3380	0,2489	0,3182	0,4441	0,3358	0,2608	0,2234	0,5234
**OGTT**	mean	29,4300	31,7400	35,7300	37,5800	30,1500	31,2000	30,9300	35,5100
	SEM	0,9990	1,0030	0,7713	1,0390	0,9993	1,2580	0,9075	1,5900
**Endothelial function aorta -10**	mean	4,7170	2,7595	0,7310	0,5307	7,4271	2,0933	2,2989	0,2283
	SEM	1,6169	1,9631	0,2060	0,1976	1,5682	0,7693	0,6528	0,2084
**Endothelial function aorta -9,5**	mean	10,9026	6,3957	2,9910	2,7968	18,7771	9,4578	8,9644	4,2650
	SEM	2,7920	3,2656	0,4649	0,5881	3,8938	2,4835	2,2792	1,1390
**Endothelial function aorta -9**	mean	11,2341	8,5327	4,4190	3,9252	22,1557	9,8911	13,5533	7,7750
	SEM	2,7046	3,8235	0,5699	0,7292	3,9087	2,2485	2,9464	1,6338
**Endothelial function aorta -8,5**	mean	25,3115	13,7050	9,7350	8,5068	29,9371	16,9811	24,6578	19,4500
	SEM	4,8431	4,3047	1,1990	1,1795	5,2532	2,4091	4,1923	3,1476
**Endothelial function aorta -8**	mean	38,4632	22,1726	15,9200	16,3931	39,8771	23,7089	36,3333	33,2733
	SEM	6,7718	5,9658	1,8237	1,8345	6,0809	1,7480	3,8325	3,8786
**Endothelial function aorta -7,5**	mean	56,7298	34,9919	29,0950	31,5208	55,8400	39,3933	51,7867	50,4833
	SEM	8,5273	7,3933	3,2824	3,2806	7,0249	2,7273	4,1550	4,8389
**Endothelial function aorta -7**	mean	73,3771	46,1373	40,4240	43,2295	68,4029	56,1600	63,5478	60,3017
	SEM	9,1464	7,5869	4,2731	3,0583	4,5177	4,2183	3,4166	5,2068
**Endothelial function aorta -6,5**	mean	83,3118	55,0499	49,4570	50,1327	79,2514	71,2644	69,3289	64,0000
	SEM	9,0583	6,5253	4,3436	3,5910	3,8248	3,3425	2,7669	5,1674
**Endothelial function aorta -6**	mean	88,1364	58,4701	55,0980	49,8485	83,0586	77,9511	68,0867	63,6783
	SEM	7,9841	6,1497	3,4581	4,0418	2,4893	4,2752	2,7594	5,3438
**Endothelial function aorta -5,5**	mean	90,4487	60,6747	60,2060	48,9953	88,9329	80,0878	72,2722	65,5983
	SEM	6,9656	5,6038	3,5203	4,1594	3,8429	5,9669	2,4829	5,4689

**Endothelial function aorta -5**	mean	90,9781	59,5332	62,5100	47,4907	88,9071	82,1611	71,0344	64,1983
	SEM	6,2568	5,3650	4,0655	4,4152	4,4771	6,7249	2,3406	5,4314
**Endothelial function aorta - AUC**	mean	262,8818	168,6375	149,4800	139,7000	267,2000	217,2667	222,6000	200,5167
	SEM	29,4745	24,0733	10,3080	9,8911	18,7426	10,6476	12,1603	17,5604
***Nos3* mRNA expression**	mean	100,0000	87,6600	94,2300	154,5000	84,7300	54,5800	62,2700	129,6000
	SEM	8,9670	18,8300	15,4200	13,9500	11,1100	9,0620	11,2500	5,5940
**eNOS protein expression**	mean	100,0000	95,3500	70,6300	58,4900	95,1300	74,1600	66,810	44,2100
	SEM	1,5670	110,1000	7,8310	6,300	11,2000	9,9330	12,2900	9,5410
**Endothelial function a. mesenterica -10**	mean	2,5011	1,6978	1,5881	2,0048	1,5302	1,0472	1,0155	1,4848
	SEM	1,0980	0,9873	0,7194	0,7703	0,7500	1,0472	1,0155	0,6294
**Endothelial function a. mesenterica -9,5**	mean	10,1251	6,4935	5,2052	5,8416	6,8341	6,7951	6,0889	7,6637
	SEM	0,9864	1,3720	1,0849	1,3204	1,0560	1,6559	1,3731	1,6700
**Endothelial function a. mesenterica -9**	mean	33,8535	28,2541	17,8014	9,6910	14,6267	23,4099	11,9989	16,8322
	SEM	6,5369	5,9590	3,0504	1,1765	2,1516	7,1826	1,3862	1,7487
**Endothelial function a. mesenterica -8,5**	mean	67,6106	63,6518	48,2689	27,5837	48,1190	58,3260	40,6152	42,2613
	SEM	4,9165	4,6018	5,4201	5,0246	5,1441	6,5822	6,4562	6,2575
**Endothelial function a. mesenterica -8**	mean	91,3277	86,9399	78,9484	63,5180	82,7680	90,4516	74,9191	79,7151
	SEM	1,7476	3,1027	2,3313	4,8825	3,1558	2,0758	4,7643	3,3887
**Endothelial function a. mesenterica -7,5**	mean	96,9015	96,2110	93,6112	90,5351	94,7892	97,9511	93,2276	95,8384
	SEM	0,6234	0,8684	1,0550	1,6867	1,1594	0,3963	0,9802	1,0658
**Endothelial function a. mesenterica -7**	mean	97,5708	97,7890	95,6540	95,8452	96,7308	98,5728	95,6377	98,4644
	SEM	0,3049	0,3281	0,9603	0,5975	0,8893	0,2968	1,3433	0,3268
**Endothelial function a. mesenterica -6,5**	mean	97,3509	97,5575	95,8325	96,6122	97,4954	98,7775	96,3389	98,3357
	SEM	0,4433	0,4564	1,0238	0,5937	0,8043	0,3372	1,4618	0,4283
**Endothelial function a. mesenterica -6**	mean	98,1221	97,5652	95,5786	96,7162	97,8572	99,3324	96,9711	98,4129

	SEM	0,2823	0,5892	1,0358	0,6533	0,7682	0,2117	1,1412	0,4720
**Endothelial function a. mesenterica - 5,5**	mean	98,8700	98,4520	96,2931	96,8938	98,1126	99,5590	97,4952	98,4023
	SEM	0,2298	0,4710	1,0095	0,7273	0,6694	0,1866	1,1942	0,4919
**Endothelial function a. mesenterica -5**	mean	99,4162	98,8811	97,5321	97,9220	98,5992	99,8736	98,5354	99,1397
	SEM	0,1851	0,4236	0,9692	0,5676	0,5718	0,1025	0,7263	0,3078
**Endothelial function a. mesenterica - L-NAME -10**	mean	0,0000	0,0000	0,0000	0,2102	0,0000	0,0000	0,4675	0,6625
	SEM	0,0000	0,0000	0,0000	0,2102	0,0000	0,0000	0,4675	0,6625
**Endothelial function a. mesenterica - L-NAME -9,5**	mean	0,3198	0,0000	0,0562	0,8501	0,5355	0,2163	0,0000	1,4797
	SEM	0,3198	0,0000	0,0562	0,5892	0,3652	0,2163	0,0000	1,0816
**Endothelial function a. mesenterica - L-NAME -9**	mean	1,2198	2,1204	0,4103	3,4033	1,4770	2,1284	0,0275	4,3947
	SEM	0,8790	1,1281	0,2825	1,2001	0,5611	1,1040	0,0185	2,0121
**Endothelial function a. mesenterica - L-NAME -8,5**	mean	9,3471	7,8054	6,0604	9,0411	5,8849	15,1740	5,4809	9,6853
	SEM	3,2942	3,1299	2,2628	3,1529	1,7058	6,7419	2,9077	3,9654
**Endothelial function a. mesenterica - L-NAME -8**	mean	34,2711	21,7795	25,5748	18,2122	16,4695	20,2918	13,5455	23,7304
	SEM	6,6604	6,3926	5,4518	4,4802	4,4827	7,9431	4,4840	7,2337
**Endothelial function a. mesenterica - L-NAME -7,5**	mean	60,9660	53,7381	50,5043	47,7902	51,0686	62,4010	45,6348	52,0554
	SEM	7,0639	5,8273	5,7764	6,3121	6,0555	5,8579	9,3223	7,8713
**Endothelial function a. mesenterica - L-NAME -7**	mean	92,5180	82,1863	84,8568	72,3186	86,0869	93,9324	82,8133	84,0899
	SEM	0,8752	4,3088	1,8435	4,8092	4,5526	1,6601	2,8673	3,6739
**Endothelial function a. mesenterica - L-NAME -6,5**	mean	95,4727	91,2372	91,9876	84,9250	92,7622	97,2557	90,2091	94,3606
	SEM	0,5070	2,4764	1,4858	3,4116	2,3509	1,3488	3,2644	1,1728
**Endothelial function a. mesenterica - L-NAME -6**	mean	96,6172	91,9125	93,3655	86,6444	94,4547	98,0147	90,7257	95,8814

	SEM	0,4549	2,7785	1,3480	3,4775	1,7342	1,3869	3,5352	0,9822
**Endothelial function a. mesenterica - L-NAME -5,5**	mean	97,4189	92,4949	94,0797	85,1830	96,4599	98,9718	91,9487	96,9086
	SEM	0,3521	2,6431	1,2049	4,1551	1,1053	1,3687	3,8862	0,8619
**Endothelial function a. mesenterica - L-NAME -5**	mean	97,7812	93,2500	94,5839	86,8770	97,1951	99,4303	94,4845	97,5960
	SEM	0,3325	2,7852	1,1672	3,9534	0,9118	1,3773	2,8254	0,8048
**Endothelial function a. mesenterica - SNP -10**	mean	0,0000	0,0000	0,0000	0,0000	0,5945	0,0000	0,0000	0,5551
	SEM	0,0000	0,0000	0,0000	0,0000	0,5945	0,0000	0,0000	0,5551
**Endothelial function a. mesenterica - SNP -9,5**	mean	0,0000	0,8354	1,3241	0,0000	0,9585	0,0000	0,0000	2,9026
	SEM	0,0000	0,8354	0,6265	0,0000	0,6536	0,0000	0,0000	2,9026
**Endothelial function a. mesenterica - SNP -9**	mean	2,2591	3,6150	5,2810	4,2639	7,9862	3,9654	2,1764	5,2548
	SEM	0,9913	1,1300	1,0162	2,1123	2,7955	1,3473	1,1624	3,4202
**Endothelial function a. mesenterica - SNP -8,5**	mean	7,9695	8,1981	12,8983	10,6223	16,0253	10,9337	8,5149	18,2179
	SEM	1,7842	1,9899	2,0294	2,9552	5,1819	2,7356	3,2558	5,1222
**Endothelial function a. mesenterica - SNP -8**	mean	21,2009	26,2777	32,7915	20,5011	40,8821	37,2973	23,3709	41,5443
	SEM	3,4507	3,9407	5,3597	3,2789	6,8464	6,2549	7,2237	7,3850
**Endothelial function a. mesenterica - SNP -7,5**	mean	42,7749	45,6272	58,9449	36,8441	64,0317	64,2478	48,8018	66,5315
	SEM	4,3192	5,8403	5,8396	4,5286	6,3817	9,3531	7,7383	8,0277
**Endothelial function a. mesenterica - SNP -7**	mean	64,7454	65,5813	69,5903	51,3141	79,7874	84,9771	68,5928	79,7357
	SEM	4,2724	4,9998	4,6089	4,1500	5,1587	5,7654	6,3074	5,9986
**Endothelial function a. mesenterica - SNP -6,5**	mean	73,3032	74,1119	73,0035	60,7029	85,5729	93,2743	82,1512	85,6359
	SEM	4,0937	4,2334	3,9274	3,4278	3,7717	2,9515	3,7210	4,2807
**Endothelial function a.**	mean	78,7725	79,6234	73,0911	65,4641	86,7575	95,0285	80,6697	81,0753

**mesenterica - SNP -6**									
	SEM	3,4170	3,3018	3,5141	3,2348	3,3113	2,7793	5,5112	5,4844
**Endothelial function a. mesenterica - SNP -5,5**	mean	81,2147	80,7881	71,2505	68,7848	86,3653	93,2582	74,5807	79,8123
	SEM	3,3134	3,4058	2,8861	3,7502	3,2867	3,1456	6,1050	6,3639
**Endothelial function a. mesenterica - SNP -5**	mean	82,3829	81,5401	69,3072	74,7921	85,2464	88,7683	76,0223	78,4947
	SEM	3,3600	3,2784	2,7128	3,5357	3,9387	4,8511	7,3721	6,5541
**Endothelial function a. mesenterica - AUC**	mean	371,3462	361,6083	338,3813	316,6077	343,6933	361,8000	331,5300	343,1154
	SEM	6,7099	6,5219	5,9396	6,5534	6,5313	7,7609	6,9142	4,5260
**Endothelial function a. mesenterica - AUC - NO-independent**	mean	268,5231	244,9750	247,1000	225,9462	246,8933	269,0333	233,9300	255,8462
	SEM	8,2369	9,7308	7,6594	13,0752	8,7735	10,4486	11,6949	11,3952
**Endothelial function a. mesenterica - AUC- NO-dependent**	mean	80,0200	116,8000	119,0273	87,2857	96,8000	92,7667	97,6000	84,7571
	SEM	11,2406	5,3634	10,4444	11,1655	6,7092	10,5601	9,3769	14,0732
**Endothelial function a. femoralis -9**	mean	4,6368	5,2849	5,1899	2,9210	2,9534	3,7935	8,0602	7,0134
	SEM	1,2988	1,1787	1,1680	1,0456	0,9938	1,3616	1,7714	1,2873
**Endothelial function a. femoralis -8,5**	mean	14,5623	12,0334	11,4072	9,6713	11,5901	10,2748	18,1085	14,6116
	SEM	1,7531	1,6938	1,0035	2,0930	1,5043	2,0470	3,3735	2,2643
**Endothelial function a. femoralis -8**	mean	37,2144	26,1847	26,0454	24,7350	36,0256	34,0474	40,8954	41,5016
	SEM	2,7548	3,1138	2,8291	3,3564	3,4415	3,7582	4,4178	3,3954
**Endothelial function a. femoralis -7,5**	mean	71,2515	50,8779	59,3542	59,9011	69,0082	72,6073	72,9589	75,3560
	SEM	1,9853	3,8830	2,8006	2,6349	3,1240	2,3885	2,8473	3,6288
**Endothelial function a. femoralis -7**	mean	86,8253	78,1201	80,2974	81,8048	87,7534	88,8188	90,2751	89,9029
	SEM	0,9119	2,5612	1,4631	1,3067	1,5376	1,2084	1,0163	2,4186
**Endothelial function a. femoralis -6,5**	mean	90,3476	87,4012	85,9255	87,7378	92,4347	91,0219	92,5930	92,9284

	SEM	0,7785	1,4690	1,0550	1,2985	1,0920	1,1718	1,0942	1,8717
**Endothelial function a. femoralis -6**	mean	90,3991	89,1331	85,3859	87,0786	92,7797	90,0979	91,8739	92,5828
	SEM	0,7441	1,2262	1,0848	1,5399	0,9252	1,2977	1,1410	1,7638
**Endothelial function a. femoralis -5,5**	mean	89,6737	87,3298	84,0378	85,3138	92,2493	90,0387	91,5027	91,6897
	SEM	0,6870	1,2956	1,0288	1,5531	0,9476	1,1746	1,1469	1,6665
**Endothelial function a. femoralis -5**	mean	90,6567	87,3300	86,2016	86,0489	93,3184	91,0931	92,7354	93,3558
	SEM	0,6716	1,4941	1,0676	1,5826	0,9744	1,0763	0,9945	1,5651
**Endothelial function a. femoralis - L-NAME -9**	mean	0,0000	0,4671	0,0000	0,0000	0,0000	0,0000	0,0000	0,0000
	SEM	0,0000	0,4671	0,0000	0,0000	0,0000	0,0000	0,0000	0,0000
**Endothelial function a. femoralis - L-NAME -8,5**	mean	0,8393	1,0200	0,0000	0,5983	0,0000	0,0000	0,0000	0,7995
	SEM	0,5736	0,8160	0,0000	0,5983	0,0000	0,0000	0,0000	0,7995
**Endothelial function a. femoralis - L-NAME -8**	mean	4,9658	2,3017	6,4192	1,6460	2,7265	1,7125	1,4451	3,9766
	SEM	1,8732	1,6510	1,6608	1,2277	1,9409	0,8470	0,7936	1,8166
**Endothelial function a. femoralis - L-NAME -7,5**	mean	12,7726	11,9962	17,0928	10,3169	12,3517	8,5532	19,4041	17,3791
	SEM	2,2693	2,2767	3,2476	2,8373	2,9140	2,2050	3,6216	4,8819
**Endothelial function a. femoralis - L-NAME -7**	mean	39,6326	30,2003	45,7158	34,7152	47,7884	35,2154	55,0743	43,2632
	SEM	2,6886	3,3136	4,8134	5,8286	4,7143	3,5255	3,8379	6,7587
**Endothelial function a. femoralis - L-NAME -6,5**	mean	72,3227	62,2015	77,7941	66,9702	77,9352	72,8469	79,6388	68,1758
	SEM	2,0582	2,8347	2,9106	4,2052	1,8108	2,0333	2,5869	6,3339
**Endothelial function a. femoralis - L-NAME -6**	mean	84,9141	82,9849	87,3954	82,1724	87,0340	83,0460	86,3060	78,5231
	SEM	1,1454	1,0955	1,0749	2,3651	0,8964	0,9693	1,9439	4,3982
**Endothelial function a. femoralis - L-NAME -5,5**	mean	83,6254	83,1192	86,6753	80,9964	85,8578	81,6676	83,1012	75,7651
	SEM	1,4994	1,2248	1,2710	2,3042	0,9292	1,0009	2,2337	6,1303

**Endothelial function a. femoralis - L-NAME -5**	mean	79,0623	78,1520	83,9050	71,3905	84,1498	77,7369	79,7961	72,8516
	SEM	2,5589	1,8491	2,2227	4,1486	1,4728	2,2922	2,3175	7,3914
**Endothelial function a. femoralis - SNP -9**	mean	20,2012	22,3636	19,8051	25,0826	4,7809	14,2747	18,6121	20,2700
	SEM	3,2039	2,2168	2,9383	3,0102	2,2757	3,6719	3,6554	4,1753
**Endothelial function a. femoralis - SNP -8,5**	mean	44,6588	40,7691	45,0599	47,6304	29,4083	38,3156	45,1492	42,0532
	SEM	3,4122	2,9420	4,4884	4,6435	4,0014	3,8321	4,3903	6,2694
**Endothelial function a. femoralis - SNP -8**	mean	66,7691	69,0964	69,2277	74,7049	67,1283	74,9103	77,9025	75,2372
	SEM	3,1823	3,4937	3,5116	4,0666	4,5313	2,7545	3,0072	5,0649
**Endothelial function a. femoralis - SNP -7,5**	mean	84,3390	85,4022	86,6153	88,9682	86,2419	88,5206	92,2339	91,8865
	SEM	2,1084	1,9407	1,5936	2,4297	3,5620	2,1776	1,3702	2,3903
**Endothelial function a. femoralis - SNP -7**	mean	94,7347	96,3954	95,5678	96,8171	96,6948	94,9870	98,0877	97,2613
	SEM	0,9606	0,6720	0,6386	1,1100	1,3781	1,7778	0,5097	0,9749
**Endothelial function a. femoralis - SNP -6,5**	mean	97,4830	99,4903	98,7150	99,1572	99,1976	97,1534	99,2660	98,4803
	SEM	0,5927	0,3155	0,4113	0,4261	0,4010	1,2532	0,2717	0,5930
**Endothelial function a. femoralis - SNP -6**	mean	98,7388	99,7224	99,4252	99,5579	99,7721	97,5188	99,6752	99,0237
	SEM	0,3963	0,2652	0,4457	0,2396	0,1532	1,0571	0,1632	0,3959
**Endothelial function a. femoralis - SNP -5,5**	mean	98,3992	99,7066	99,5425	99,6414	99,7424	97,4173	99,7041	99,0900
	SEM	0,4288	0,2653	0,3205	0,1951	0,1330	1,0768	0,1530	0,3763
**Endothelial function a. femoralis - SNP -5**	mean	98,6795	99,7344	99,8257	99,7558	99,9016	97,7914	99,6817	99,3450
	SEM	0,3885	0,2656	0,3230	0,1364	0,0984	1,0417	0,1639	0,3149
**Endothelial function a. femoralis - AUC**	mean	263,2941	238,6895	239,0750	240,3571	264,9692	262,1769	274,2929	274,3692

	SEM	3,8773	7,2286	4,7552	5,0510	5,5402	5,9073	6,7670	8,2353
**Endothelial function a. femoralis - AUC - NO-independent**	mean	169,3063	156,5737	181,5188	156,5500	177,8923	160,9538	182,4286	162,1654
	SEM	5,1640	5,5013	7,0983	9,1066	5,6281	4,4372	6,4673	14,9705
**Endothelial function a. femoralis - AUC- NO-dependent**	mean	94,6500	82,1158	57,5563	83,8071	87,0769	161,4923	153,1643	169,2500
	SEM	6,9958	7,8067	6,2288	7,8472	3,5503	7,1757	9,0968	16,4980
**Superoxide production**	mean	72,1000	130,4000	194,9000	303,0000	104,3000	81,5500	111,5000	254,4000
	SEM	13,5700	49,1500	12,3800	49,8900	16,8800	18,6400	16,7600	43,8700
**Nrf2 protein expression**	mean	100,0000	101,8000	74,6200	71,5600	104,6000	89,5100	88,3800	70,3200
	SEM	4,2920	25,3700	17,1500	12,1900	10,7400	9,7020	14,8600	10,1100
**SOD1 protein expression**	mean	100,0000	69,7500	71,2600	52,3800	86,2500	103,3000	136,4000	47,3600
	SEM	5,8490	10,5300	13,9000	10,6200	14,3000	8,2120	15,9600	5,0040
**HO-1 protein expression**	mean	100,0000	129,2000	75,2900	72,5200	143,5000	131,0000	149,4000	73,8200
	SEM	4,3510	11,2100	5,5640	11,1100	26,4900	26,8900	19,8500	8,5370
***Tnf* mRNA expression**	mean	100,0000	106,7000	148,9000	161,2000	55,9500	71,7300	79,3900	104,7000
	SEM	11,5200	11,2500	13,2300	12,2800	5,6120	17,1500	13,1600	8,8340
***Inos* mRNA expression**	mean	100,0000	158,2000	206,8000	230,5000	61,8000	136,4000	79,3200	180,6000
	SEM	13,2200	18,0700	6,4890	43,8000	5,9570	33,8100	18,2000	15,0800
***Il1b* mRNA expression**	mean	100,0000	103,4000	161,0000	157,3000	63,1000	49,2300	85,2100	123,2000
	SEM	9,6680	9,4470	7,6380	15,9700	3,7740	19,2300	20,5200	18,7900
***Cox2* mRNA expression**	mean	100,0000	89,3500	141,2000	147,4000	95,6200	100,4000	104,4000	208,7000
	SEM	11,8400	1,4060	13,8500	9,1910	13,0700	41,7200	26,0100	29,9700
**p-α1-AMPK(Thr172)/α1-AMPK ratio**	mean	100,0000	74,6000	60,1800	62,6800	75,9200	52,4300	54,4000	42,2300
	SEM	4,1560	9,5500	4,9400	6,3160	3,8230	6,6340	8,1180	8,9980
**Estradiol**	mean	171,6000	160,9000	191,9000	229,8000	207,2000	171,7000	166,1000	137,7000
	SEM	13,8000	10,0300	15,8400	60,6200	13,8400	19,8000	7,6160	5,5930
**Testosterone**	mean	28,3800	36,6500	31,6300	30,0600	8,8370	9,0450	9,3270	9,0580
	SEM	3,8950	6,1950	4,3580	10,5900	0,9770	1,2670	0,4478	0,6038
**SOD activity plasma**	mean	100,0000	34,1300	88,5300	16,9000	76,0000	30,0000	116,5000	22,0500

	SEM	9,3100	13,5200	6,0420	1,1770	6,9460	6,9390	21,4800	5,4110
**State 3 G + M**	mean	99,5000	105,0000	111,0000	101,3000	103,1000	97,6200	116,0000	103,0000
	SEM	2,2430	1,8820	3,0190	3,2350	2,3010	2,7680	7,0120	2,6030
**State 3 Succ.**	mean	125,4000	132,0000	136,6000	124,6000	128,8000	126,3000	145,6000	132,5000
	SEM	2,0720	2,7140	3,6140	2,7030	2,6180	3,5560	7,9540	3,2830
**State 4 G + M**	mean	16,8500	17,1900	18,7700	17,2300	18,3700	17,5700	19,0800	17,2400
	SEM	0,7477	0,6357	0,8839	0,8341	0,7322	0,7173	1,6680	1,3220
**State 4 Succ.**	mean	26,2900	27,7500	30,4500	28,4200	29,9600	28,7200	31,4000	30,7800
	SEM	1,2570	1,0860	1,5270	1,4350	1,3160	1,2800	2,5570	1,7770
**State 3/State 4 G + M**	mean	5,9640	6,1780	5,9780	5,9800	5,6600	5,6120	6,1810	6,2810
	SEM	0,2317	0,2358	0,1844	0,3107	0,1921	0,2875	0,2329	0,6272
**State 3/State 4 Succ.**	mean	4,8260	4,8040	4,5440	4,4790	4,3520	4,4490	4,7120	4,4010
	SEM	0,1849	0,1563	0,1526	0,2338	0,1917	0,2390	0,1729	0,2693
**OPR G + M**	mean	365,5000	409,1000	437,6000	380,2000	383,2000	382,0000	467,7000	399,5000
	SEM	7,2020	10,2000	14,7600	12,1700	20,9700	15,8400	24,9900	15,2400
**OPR Succ.**	mean	324,1000	334,0000	324,6000	310,7000	327,4000	315,7000	374,5000	322,8000
	SEM	9,3440	9,0810	10,9300	9,0010	9,7280	12,6000	21,1800	12,2100
**ADP:O G + M**	mean	2,1370	2,2480	2,2840	2,1840	2,1560	2,2750	2,3350	2,2470
	SEM	0,0757	0,0493	0,0504	0,0406	0,0881	0,0342	0,0312	0,0594
**ADP:O Succ.**	mean	1,5980	1,5580	1,5350	1,5730	1,5970	1,5590	1,5960	1,5380
	SEM	0,0293	0,0190	0,0097	0,0243	0,0235	0,0477	0,0184	0,0161
**SOD activity mitochondria**	mean	100,0000	109,9000	143,8000	169,7000	127,1000	108,8000	140,5000	180,3000
	SEM	7,8000	5,4780	10,2800	9,1580	5,6520	8,5270	6,3980	16,6400
**TBARS**	mean	7,1040	7,7820	6,8410	6,5350	8,3830	8,6330	7,9580	7,6640
	SEM	0,3279	0,2164	0,2304	0,1464	0,5996	0,5757	0,5990	0,9202
**MitoSOX**	mean	76,1400	98,6300	72,3300	56,1300	87,5700	126,9000	86,5600	84,1400
	SEM	2,5770	6,7660	6,8270	6,3370	8,3920	11,3900	7,3520	6,6810
**Max. relaxation aorta**	mean	93,2800	62,9800	62,9700	52,5800	91,4000	85,5600	74,4100	67,5400
	SEM	7,0790	5,7120	4,1260	4,3460	3,4330	4,8280	2,8550	5,8440
**EDCF aorta**	mean	2,3050	3,4450	0,4600	5,0890	2,4960	3,3960	3,3710	3,3370
	SEM	1,2150	2,4160	0,3285	1,7480	1,8740	3,3960	1,9180	1,7870
**Max. relaxation a. mesenterica**	mean	99,4200	99,0500	97,5300	97,9300	98,6400	99,8700	98,5400	99,2300

	SEM	0,1851	0,3250	0,9692	0,5644	0,5770	0,1025	0,7263	0,2888
**Max. relaxation a. mesenterica + L-NAME**	mean	97,8300	93,6200	94,9000	88,3400	97,2200	99,4500	94,7300	97,6800
	SEM	0,3223	2,5170	1,1440	3,5140	0,9148	1,3740	2,6060	0,8060
**EDCF a. mesenterica**	mean	0,0000	0,1663	0,0000	0,0128	0,0378	0,0000	0,0000	0,0869
	SEM	0,0000	0,1320	0,0000	0,0128	0,0378	0,0000	0,0000	0,0598
**EDCF a. mesenterica + L-NAME**	mean	0,0439	0,3662	0,3145	1,4650	0,0247	0,0185	0,2480	0,0834
	SEM	0,0321	0,2796	0,2194	1,0940	0,0247	0,0185	0,2312	0,0834
**Max. relaxation a. femoralis**	mean	91,3700	89,4000	87,0600	88,5000	93,8100	92,0000	93,6100	93,8100
	SEM	0,7266	1,2880	1,0790	1,3490	0,9354	1,0480	0,9191	1,6530
**Max. relaxation a.femoralis + L-NAME**	mean	85,1500	83,9700	87,9400	82,4900	87,7300	83,7900	86,4100	79,5900
	SEM	1,1550	1,0860	1,1320	2,3220	0,9799	0,9971	1,9560	4,5890
**EDCF a. femoralis**	mean	0,7161	2,0690	0,8593	2,4540	0,4878	0,9079	0,8785	0,4562
	SEM	0,2636	0,4137	0,2813	0,5485	0,1754	0,2987	0,3137	0,2052
**EDCF a. femoralis + L-NAME**	mean	6,0910	5,8170	4,0310	11,1000	3,3230	6,0540	6,6150	6,7420
	SEM	1,5600	0,9666	1,3000	2,5680	0,9251	2,1240	0,7865	2,9390

Supplementary Table S2. Two-way ANOVA statistical analysis results – Source of Variation.

	interaction	group	sex
a.femoralis AUC NO-dependent	**<0,0001**	**0,0003**	**<0,0001**
a.femoralis AUC NO-independent	0,9676	**0,0058**	0,3724
a.femoralis AUC total	**0,0264**	0,1680	**<0,0001**
a.mesenterica AUC NO-dependent	0,5416	0,4444	0,3405
a.mesenterica AUC NO-independent	**0,0238**	0,1567	0,5121
a.mesenterica AUC total	**0,0005**	**<0,0001**	0,6740
ADP:O G+M	0,9771	**0,0383**	0,3179
ADP:O Succ.	0,2688	0,3108	0,7111
Aortic superoxide production	0,1398	**<0,0001**	0,0628
Body weight	**0,0445**	**<0,0001**	**<0,0001**
Cox2 mRNA expression	**0,0242**	**<0,0001**	0,6261
EDCF a. femoralis	**0,0194**	**0,0262**	**0,0008**
EDCF a. femoralis + L-NAME	0,1968	0,0887	0,3789
EDCF a. mesenterica	0,1483	0,4529	0,7170
EDCF a. mesenterica + L-NAME	0,3374	0,3308	0,1432
EDCF aorta	0,7083	0,6793	0,8206
Endothelial function - aorta AUC	0,3513	**<0,0001**	**0,0026**
*Nos3*mRNA expression	0,9799	**0,0031**	**0,0176**
eNOS protein expression	0,8004	**<0,0001**	0,1202

Estradiol	0,0615	0,8215	0,3095
Glycemia	**0,0242**	**<0,0001**	0,6261
HO-1 protein expression	0,1379	**0,0105**	**0,0179**
Il1b mRNA expression	0,3990	**<0,0001**	**<0,0001**
Inos mRNA expression	0,0813	**<0,0001**	**0,0003**
Max. relaxation a. femoralis	0,2099	0,2389	**<0,0001**
Max. relaxation a. mesenterica	0,2868	0,1374	0,1792
Max. relaxation a. mesenterica + L-NAME	**0,0214**	0,0802	**0,0088**
Max. relaxation a.femoralis + L-NAME	0,5720	**0,0124**	0,7194
Max. relaxation aorta	0,1336	**<0,0001**	**0,0025**
MitoSOX	0,5471	**<0,0001**	**0,0003**
Nrf2 protein expression	0,6459	**0,0115**	0,8645
OGTT	0,0700	**<0,0001**	**0,0338**
OPR G+M	0,2902	**<0,0001**	0,3723
OPR Succ.	**0,0466**	**0,0450**	0,1739
p-α1AMPK/α1AMPK	0,5194	**<0,0001**	**0,0003**
rWAT/BW %	0,9319	**<0,0001**	0,5830
sBP	0,1317	**<0,0001**	**<0,0001**
SOD activity mitochondria	0,4229	**<0,0001**	0,2410
SOD activity plasma	0,1016	**<0,0001**	0,8650
SOD1 protein expression	**0,0021**	**<0,0001**	**0,0110**
State 3 G+M	0,2852	**0,0010**	0,7668
State 3 Succ.	0,2241	**0,0016**	0,1876
State 4 G+M	>0,9999	0,0764	>0,9999
State 4 Succ.	0,8031	0,2525	0,0810
State3 /State 4 Succ.	0,3967	0,7535	0,2037
State3 /State 4 G+M	0,4571	0,7083	0,6790
TAG	**0,0453**	**<0,0001**	**0,0463**
Testosterone	0,8450	0,8314	**<0,0001**
Tnf mRNA expression	0,4984	**0,0001**	**<0,0001**

Supplementary Table S3. Four-parameter logistic (4PL) nonlinear regression analysis results.

**Endothelial function**		**male**				**female**			
**Aorta**	**Bottom**	4,078	3,412	-0,2166	2,001	6,448	5,559	1,265	0,4541
	**Top**	92,33	61,38	63,8	49,65	92,85	84,19	72,29	65,34
	**LogIC50**	-7,75	-7,606	-7,346	-7,703	-7,724	-7,364	-8,025	-8,053
	**HillSlope**	0,735	0,7674	0,6491	1,151	0,5463	0,7468	0,7496	0,9377
	**IC50**	1,776E-08	2,48E-08	4,513E-08	1,983E-08	1,886E-08	4,329E-08	9,447E-09	8,841E-09
	**Span**	88,25	57,97	64,02	47,65	86,4	78,63	71,02	64,89
	**Null hypothesis**	One curve for all data sets				One curve for all data sets			
	**Alternative hypothesis**	Different curve for at least one data set				Different curve for each data set			
	**P value**	<0,0001				<0,0001			
	**Conclusion (alpha = 0.05)**	Reject null hypothesis				Reject null hypothesis			
	**Preferred model**	Different curve for at least one data set				Different curve for each data set			
	**F (DFn, DFd)**	18,77 (12, 391)				8,359 (12, 303)			
**a. femoralis**	**Bottom**	3,584	6,275	5,214	2,945	1,026	4,391	5,335	5,361

	**Top**	90,09	89,57	85,63	86,95	93,57	91,81	92,64	91,24
	**LogIC50**	-7,861	-7,637	-7,7	-7,735	-7,852	-7,819	-7,896	-7,886
	**HillSlope**	1,504	1,314	1,506	1,538	1,357	1,679	1,366	1,528
	**IC50**	1,378E-08	2,308E-08	1,995E-08	1,842E-08	1,407E-08	1,516E-08	1,269E-08	1,3E-08
	**Span**	86,51	83,29	80,41	84,01	92,54	87,42	87,31	85,88
	**Null hypothesis**	One curve for all data sets				One curve for all data sets			
	**Alternative hypothesis**	Different curve for at least one data set				Different curve for at least one data set			
	**P value**	<0,0001				0,0885			
	**Conclusion (alpha = 0.05)**	Reject null hypothesis				Do not reject null hypothesis			
	**Preferred model**	Different curve for at least one data set				One curve for all data sets			
	**F (DFn, DFd)**	5,565 (12, 308)				1,611 (12, 263)			
**a. femoralis + L-NAME**	**Bottom**	2,088	1,945	1,014	0,6385	0,3086	1,108	-0,3505	0,6159
	**Top**	84,08	82,64	87,34	78,15	85,83	80,83	82,4	75,46
	**LogIC50**	-6,986	-6,848	-7,071	-6,931	-7,075	-6,934	-7,152	-7,045
	**HillSlope**	1,557	1,455	1,496	1,835	1,79	2,06	1,842	1,556
	**IC50**	1,033E-07	1,419E-07	8,5E-08	1,173E-07	8,414E-08	1,164E-07	7,049E-08	9,02E-08
	**Span**	81,99	80,7	86,33	77,51	85,52	79,72	82,75	74,85
	**Null hypothesis**	One curve for all data sets				One curve for all data sets			
	**Alternative hypothesis**	Different curve for at least one data set				Different curve for at least one data set			
	**P value**	<0,0001				<0,0001			
	**Conclusion (alpha = 0.05)**	Reject null hypothesis				Reject null hypothesis			
	**Preferred model**	Different curve for at least one data set				Different curve for at least one data set			
	**F (DFn, DFd)**	4,805 (12, 308)				4,895 (12, 254)			
**a. femoralis SNP**	**Bottom**	-3,884	11,23	-14,99	7,44	-10,68	5,916	2,731	14,09
	**Top**	99,49	100,1	100,7	99,74	99,8	98,14	99,69	99
	**LogIC50**	-8,434	-8,263	-8,599	-8,369	-8,324	-8,379	-8,491	-8,314
	**HillSlope**	0,8606	1,045	0,8329	1,033	1,158	1,414	1,203	1,436
	**IC50**	3,678E-09	5,454E-09	2,517E-09	4,274E-09	4,741E-09	4,18E-09	3,226E-09	4,853E-09
	**Span**	103,4	88,92	115,7	92,3	110,5	92,22	96,96	84,91
	**Null hypothesis**	One curve for all data sets				One curve for all data sets			
	**Alternative hypothesis**	Different curve for each data set				Different curve for at least one data set			
	**P value**	0,7655				<0,0001			
	**Conclusion (alpha = 0.05)**	Do not reject null hypothesis				Reject null hypothesis			
	**Preferred model**	One curve for all data sets				Different curve for at least one data set			
	**F (DFn, DFd)**	0,6853 (12, 308)				6,626 (12, 254)			
**a. mesenterica**	**Bottom**	0,395	-0,5618	-0,1193	3,54	2,004	1,576	0,7903	2,006
	**Top**	98,56	98,33	97,03	98,05	97,96	99,57	97,54	99,1
	**LogIC50**	-8,758	-8,674	-8,569	-8,208	-8,461	-8,622	-8,403	-8,472
	**HillSlope**	1,323	1,304	1,225	1,458	1,512	1,475	1,361	1,441
	**IC50**	1,745E-09	2,116E-09	2,695E-09	6,194E-09	3,456E-09	2,387E-09	3,949E-09	3,375E-09
	**Span**	98,16	98,89	97,15	94,51	95,96	97,99	96,75	97,09
	**Null hypothesis**	One curve for all data sets				One curve for all data sets			
	**Alternative hypothesis**	Different curve for at least one data set				Different curve for each data set			
	**P value**	<0,0001				<0,0001			
	**Conclusion (alpha = 0.05)**	Reject null hypothesis				Reject null hypothesis			
	**Preferred model**	Different curve for at least one data set				Different curve for each data set			

	**F (DFn, DFd)**	18,25 (12, 358)				3,831 (12, 303)			
**a. mesenterica + L-NAME**	**Bottom**	-0,3234	1,126	0,2392	1,796	1,025	2,83	0,767	2,868
	**Top**	98,24	91,75	93,4	88,46	98,55	100,2	91,25	97,37
	**LogIC50**	-7,762	-7,626	-7,577	-7,567	-7,7	-7,655	-7,513	-7,583
	**HillSlope**	1,218	1,382	1,29	1,317	1,271	1,542	1,694	1,187
	**IC50**	1,73E-08	2,364E-08	2,65E-08	2,711E-08	1,996E-08	2,214E-08	3,068E-08	2,612E-08
	**Span**	98,56	90,62	93,16	86,66	97,52	97,38	90,49	94,5
	**Null hypothesis**	One curve for all data sets				One curve for all data sets			
	**Alternative hypothesis**	Different curve for at least one data set				Different curve for each data set			
	**P value**	<0,0001				0,0008			
	**Conclusion (alpha = 0.05)**	Reject null hypothesis				Reject null hypothesis			
	**Preferred model**	Different curve for at least one data set				Different curve for each data set			
	**F (DFn, DFd)**	3,597 (12, 358)				2,894 (12, 303)			
**a. mesenterica SNP**	**Bottom**	-0,6532	-0,7103	1,109	-2,385	0,242	-0,3918	0,1419	1,77
	**Top**	82,36	82,99	69,06	75,71	87,91	93,58	77,71	81,87
	**LogIC50**	-7,524	-7,547	-7,797	-7,413	-8,004	-7,838	-7,769	-7,908
	**HillSlope**	0,9543	0,8724	1,233	0,6661	1,088	1,246	1,244	0,9852
	**IC50**	2,991E-08	2,838E-08	1,594E-08	3,864E-08	9,908E-09	1,452E-08	1,701E-08	1,237E-08
	**Span**	83,02	83,7	67,95	78,09	87,67	93,97	77,57	80,1
	**Null hypothesis**	One curve for all data sets				One curve for all data sets			
	**Alternative hypothesis**	Different curve for at least one data set				Different curve for at least one data set			
	**P value**	<0,0001				0,0036			
	**Conclusion (alpha = 0.05)**	Reject null hypothesis				Reject null hypothesis			
	**Preferred model**	Different curve for at least one data set				Different curve for at least one data set			
	**F (DFn, DFd)**	5,251 (12, 358)				2,516 (12, 303)			

Supplementary Table S4. Kruskal-Wallis test analysis results – EC50 endothelial function.

**Kruskal-Wallis test**				
**a. femoralis**	Variables: m_CTR, m_Cmpd C, m_HFD, m_HFD+ mpd C		Variables: f_CTR, f_Cmpd C, f_HFD, f_HFD+Cmpd C	
	Mean rank: 9.56, 24.1, 20.75, 18.25		Mean rank: 16, 19.14, 14.57, 14.29	
	Groups = 4		Groups = 4	
	df = 3		df = 3	
	Total observations = 36		Total observations = 31	
	T = 8.952759		T = 1.258065	
	P = 0.0299		P = 0.7391	
	Critical t (32 df, 2-sided) = 2.036933		Critical t (27 df, 2-sided) = 2.051831	
	m_CTR vs m_Cmpd C	significant	f_CTR vs f_Cmpd C	not significant
	(14.5375 > 9.184075)	P = 0.0029	(3.142857 > 9.485457)	P = 0.5024
	m_CTR vs m_HFD	significant	f_CTR vs f_HFD	not significant
	(11.1875 > 9.680865)	P = 0.0249	(1.428571 > 9.485457)	P = 0.7597
	m_CTR vs m_HFD+ mpd C	not significant	f_CTR vs f_HFD+Cmpd C	not significant
	(8.6875 > 9.184075)	P = 0.0629	(1.714286 > 9.485457)	P = 0.7137
	m_Cmpd C vs m_HFD	not significant	f_Cmpd C vs f_HFD	not significant

	(3.35 > 9.184075)	P = 0.4629	(4.571429 > 10.288423)	P = 0.37
	m_Cmpd C vs m_HFD+ mpd C	not significant	f_Cmpd C vs f_HFD+Cmpd C	not significant
	(5.85 > 8.658829)	P = 0.1783	(4.857143 > 10.288423)	P = 0.3413
	m_HFD vs m_HFD+ mpd C	not significant	f_HFD vs f_HFD+Cmpd C	not significant
	(2.5 > 9.184075)	P = 0.5831	(0.285714 > 10.288423)	P = 0.955
**a. femoralis + L-NAME**	Variables: m_CTR, m_Cmpd C, m_HFD, m_HFD+ mpd C		Variables: f_CTR, f_Cmpd C, f_HFD, f_HFD+Cmpd C	
	Mean rank: 14.38, 24, 12.5, 21.1		Mean rank: 14.22, 24.14, 8.86, 15.14	
	Groups = 4		Groups = 4	
	df = 3		df = 3	
	Total observations = 36		Total observations = 30	
	T = 7.15518		T = 10.933845	
	P = 0.0671		P = 0.0121	
	Critical t (32 df, 2-sided) = 2.036933		Critical t (26 df, 2-sided) = 2.055529	
	m_CTR vs m_Cmpd C	significant	f_CTR vs f_Cmpd C	significant
	(9.625 > 9.495696)	P = 0.0471	(9.920635 > 7.601695)	P = 0.0125
	m_CTR vs m_HFD	not significant	f_CTR vs f_HFD	not significant
	(1.875 > 10.009342)	P = 0.7053	(5.365079 > 7.601695)	P = 0.1588
	m_CTR vs m_HFD+ mpd C	not significant	f_CTR vs f_HFD+Cmpd C	not significant
	(6.725 > 9.495696)	P = 0.1589	(0.920635 > 7.601695)	P = 0.8054
	m_Cmpd C vs m_HFD	significant	f_Cmpd C vs f_HFD	significant
	(11.5 > 9.495696)	P = 0.0192	(15.285714 > 8.062815)	P = 0.0006
	m_Cmpd C vs m_HFD+ mpd C	not significant	f_Cmpd C vs f_HFD+Cmpd C	significant
	(2.9 > 8.952628)	P = 0.5141	(9 > 8.062815)	P = 0.0301
	m_HFD vs m_HFD+ mpd C	not significant	f_HFD vs f_HFD+Cmpd C	not significant
	(8.6 > 9.495696)	P = 0.0743	(6.285714 > 8.062815)	P = 0.1211
**a. femoralis - SNP**	Variables: m_CTR, m_Cmpd C, m_HFD, m_HFD+ mpd C		Variables: f_CTR, f_Cmpd C, f_HFD, f_HFD+Cmpd C	
	Mean rank: 18.06, 23.85, 12, 18.7		Mean rank: 18.11, 15.57, 9.57, 18	
	Groups = 4		Groups = 4	
	df = 3		df = 3	
	Total observations = 36		Total observations = 30	
	T = 5.641047		T = 4.531388	
	P = 0.1304		P = 0.2095	
	Critical t (32 df, 2-sided) = 2.036933		Critical t (26 df, 2-sided) = 2.055529	
	m_CTR vs m_Cmpd C	not significant	f_CTR vs f_Cmpd C	not significant
	(5.7875 > 9.750454)	P = 0.2355	(2.539683 > 8.84672)	P = 0.5602
	m_CTR vs m_HFD	not significant	f_CTR vs f_HFD	not significant
	(6.0625 > 10.277881)	P = 0.2384	(8.539683 > 8.84672)	P = 0.0579
	m_CTR vs m_HFD+ mpd C	not significant	f_CTR vs f_HFD+Cmpd C	not significant
	(0.6375 > 9.750454)	P = 0.8949	(0.111111 > 8.84672)	P = 0.9796
	m_Cmpd C vs m_HFD	significant	f_Cmpd C vs f_HFD	not significant
	(11.85 > 9.750454)	P = 0.0188	(6 > 9.383363)	P = 0.2002
	m_Cmpd C vs m_HFD+ mpd C	not significant	f_Cmpd C vs f_HFD+Cmpd C	not significant

	(5.15 > 9.192817)	P = 0.2623	(2.428571 > 9.383363)	P = 0.5992
	m_HFD vs m_HFD+ mpd C	not significant	f_HFD vs f_HFD+Cmpd C	not significant
	(6.7 > 9.750454)	P = 0.1712	(8.428571 > 9.383363)	P = 0.0763
**a. mesenterica**	Variables: m_CTR, m_Cmpd C, m_HFD, m_HFD+ mpd C		Variables: f_CTR, f_Cmpd C, f_HFD, f_HFD+Cmpd C	
	Mean rank: 10.21, 13.75, 16.25, 27.89		Mean rank: 15.8, 9.08, 19.08, 15.43	
	Groups = 4		Groups = 4	
	df = 3		df = 3	
	Total observations = 34		Total observations = 29	
	T = 14.834194		T = 4.383021	
	P = 0.002		P = 0.223	
	Critical t (30 df, 2-sided) = 2.042272		Critical t (25 df, 2-sided) = 2.059539	
	m_CTR vs m_Cmpd C	not significant	f_CTR vs f_Cmpd C	not significant
	(3.535714 > 8.190584)	P = 0.385	(6.716667 > 8.801678)	P = 0.1286
	m_CTR vs m_HFD	not significant	f_CTR vs f_HFD	not significant
	(6.035714 > 7.798996)	P = 0.1245	(3.283333 > 8.801678)	P = 0.4495
	m_CTR vs m_HFD+ mpd C	significant	f_CTR vs f_HFD+Cmpd C	not significant
	(17.674603 > 7.975409)	P < 0.0001	(0.371429 > 8.39956)	P = 0.9282
	m_Cmpd C vs m_HFD	not significant	f_Cmpd C vs f_HFD	significant
	(2.5 > 7.506794)	P = 0.5016	(10 > 9.840575)	P = 0.0467
	m_Cmpd C vs m_HFD+ mpd C	significant	f_Cmpd C vs f_HFD+Cmpd C	not significant
	(14.138889 > 7.689914)	P = 0.0007	(6.345238 > 9.482615)	P = 0.1804
	m_HFD vs m_HFD+ mpd C	significant	f_HFD vs f_HFD+Cmpd C	not significant
	(11.638889 > 7.271413)	P = 0.0027	(3.654762 > 9.482615)	P = 0.4348
**a. mesenterica + L-NAME**	Variables: m_CTR, m_Cmpd C, m_HFD, m_HFD+ mpd C		Variables: f_CTR, f_Cmpd C, f_HFD, f_HFD+Cmpd C	
	Mean rank: 17.14, 20.13, 22, 10.44		Mean rank: 13.2, 12.83, 17.67, 17.14	
	Groups = 4		Groups = 4	
	df = 3		df = 3	
	Total observations = 34		Total observations = 29	
	T = 7.12483		T = 1.867258	
	P = 0.068		P = 0.6004	
	Critical t (30 df, 2-sided) = 2.042272		Critical t (25 df, 2-sided) = 2.059539	
	m_CTR vs m_Cmpd C	not significant	f_CTR vs f_Cmpd C	not significant
	(2.982143 > 9.775278)	P = 0.538	(0.366667 > 9.25861)	P = 0.9356
	m_CTR vs m_HFD	not significant	f_CTR vs f_HFD	not significant
	(4.857143 > 9.307927)	P = 0.2951	(4.466667 > 9.25861)	P = 0.3299
	m_CTR vs m_HFD+ mpd C	not significant	f_CTR vs f_HFD+Cmpd C	not significant
	(6.698413 > 9.518472)	P = 0.161	(3.942857 > 8.835617)	P = 0.3668
	m_Cmpd C vs m_HFD	not significant	f_Cmpd C vs f_HFD	not significant
	(1.875 > 8.959191)	P = 0.6721	(4.833333 > 10.35144)	P = 0.3454
	m_Cmpd C vs m_HFD+ mpd C	significant	f_Cmpd C vs f_HFD+Cmpd C	not significant
	(9.680556 > 9.17774)	P = 0.0394	(4.309524 > 9.974898)	P = 0.3821

	m_HFD vs m_HFD+ mpd C	significant	f_HFD vs f_HFD+Cmpd C	not significant
	(11.555556 > 8.678268)	P = 0.0108	(0.52381 > 9.974898)	P = 0.9147
**a. mesenterica - SNP**	Variables: m_CTR, m_Cmpd C, m_HFD, m_HFD+ mpd C		Variables: f_CTR, f_Cmpd C, f_HFD, f_HFD+Cmpd C	
	Mean rank: 20.36, 19.56, 14.5, 16.78		Mean rank: 13.4, 15.67, 18.67, 13.57	
	Groups = 4		Groups = 4	
	df = 3		df = 3	
	Total observations = 34		Total observations = 29	
	T = 1.874305		T = 1.699573	
	P = 0.5989		P = 0.637	
	Critical t (30 df, 2-sided) = 2.042272		Critical t (25 df, 2-sided) = 2.059539	
	m_CTR vs m_Cmpd C	not significant	f_CTR vs f_Cmpd C	not significant
	(0.794643 > 10.72129)	P = 0.8807	(2.266667 > 9.288267)	P = 0.6196
	m_CTR vs m_HFD	not significant	f_CTR vs f_HFD	not significant
	(5.857143 > 10.20871)	P = 0.2505	(5.266667 > 9.288267)	P = 0.2539
	m_CTR vs m_HFD+ mpd C	not significant	f_CTR vs f_HFD+Cmpd C	not significant
	(3.579365 > 10.439631)	P = 0.4892	(0.171429 > 8.863919)	P = 0.9685
	m_Cmpd C vs m_HFD	not significant	f_Cmpd C vs f_HFD	not significant
	(5.0625 > 9.826225)	P = 0.3011	(3 > 10.384598)	P = 0.5572
	m_Cmpd C vs m_HFD+ mpd C	not significant	f_Cmpd C vs f_HFD+Cmpd C	not significant
	(2.784722 > 10.065924)	P = 0.5763	(2.095238 > 10.006849)	P = 0.67
	m_HFD vs m_HFD+ mpd C	not significant	f_HFD vs f_HFD+Cmpd C	not significant
	(2.277778 > 9.518116)	P = 0.6286	(5.095238 > 10.006849)	P = 0.3044
**aorta**	Variables: m_CTR, m_Cmpd C, m_HFD, m_HFD+ mpd C		Variables: f_CTR, f_Cmpd C, f_HFD, f_HFD+Cmpd C	
	Mean rank: 15.45, 20.31, 23.6, 16.81		Mean rank: 15.43, 21.63, 11, 11	
	Groups = 4		Groups = 4	
	df = 3		df = 3	
	Total observations = 37		Total observations = 29	
	T = 3.430457		T = 7.950493	
	P = 0.3299		P = 0.047	
	Critical t (33 df, 2-sided) = 2.034515		Critical t (25 df, 2-sided) = 2.059539	
	m_CTR vs m_Cmpd C	not significant	f_CTR vs f_Cmpd C	not significant
	(4.857955 > 10.165935)	P = 0.338	(6.196429 > 8.127789)	P = 0.129
	m_CTR vs m_HFD	not significant	f_CTR vs f_HFD	not significant
	(8.145455 > 9.55928)	P = 0.0923	(4.428571 > 8.127789)	P = 0.2724
	m_CTR vs m_HFD+ mpd C	not significant	f_CTR vs f_HFD+Cmpd C	not significant
	(1.357955 > 10.165935)	P = 0.7875	(4.428571 > 8.73711)	P = 0.3065
	m_Cmpd C vs m_HFD	not significant	f_Cmpd C vs f_HFD	significant
	(3.2875 > 10.377748)	P = 0.5237	(10.625 > 7.85219)	P = 0.01
	m_Cmpd C vs m_HFD+ mpd C	not significant	f_Cmpd C vs f_HFD+Cmpd C	significant
	(3.5 > 10.939107)	P = 0.5196	(10.625 > 8.481334)	P = 0.0161
	m_HFD vs m_HFD+ mpd C	not significant	f_HFD vs f_HFD+Cmpd C	not significant

	(6.7875 > 10.377748)	P = 0.1924	(0 > 8.481334)	P > 0.9999

Supplementary Table S5. Tukey-Kramer multiple comparisons – EC50 endothelial function.

	Comparison	Mean difference L (95% CI)	|L/SE(L)|		Comparison	Mean difference L (95% CI)	|L/SE(L)|	
a. femoralis	m_CTR vs. m_Cmpd C	-0.226675(-0.428793 to -0.024557)	4.297141	P = 0.0231	f_Cmpd C vs. f_HFD	0.078286(-0.145402 to 0.301973)	1.354445	P = 0.7741
	m_CTR vs. m_HFD	-0.16175(-0.374802 to 0.051302)	2.908986	P = 0.1891	f_CTR vs. f_HFD	0.046286(-0.159944 to 0.252515)	0.868593	P = 0.9267
	m_CTR vs. m_HFD+ mpd C	-0.120675(-0.322793 to 0.081443)	2.28767	P = 0.3834	f_Cmpd C vs. f_HFD+Cmpd C	0.042(-0.181688 to 0.265688)	0.726655	P = 0.9551
	m_Cmpd C vs. m_HFD+ mpd C	0.106(-0.084559 to 0.296559)	2.131367	P = 0.4452	f_HFD vs. f_HFD+Cmpd C	-0.036286(-0.259973 to 0.187402)	0.62779	P = 0.9702
	m_Cmpd C vs. m_HFD	0.064925(-0.137193 to 0.267043)	1.230801	P = 0.82	f_CTR vs. f_Cmpd C	-0.032(-0.23823 to 0.17423)	0.600509	P = 0.9738
	m_HFD vs. m_HFD+ mpd C	0.041075(-0.161043 to 0.243193)	0.77867	P = 0.9457	f_CTR vs. f_HFD+Cmpd C	0.01(-0.19623 to 0.21623)	0.187659	P = 0.9991
a. femoralis + L-NAME	m_Cmpd C vs. m_HFD	0.201175(-0.044473 to 0.446823)	3.137923	P = 0.1398	f_Cmpd C vs. f_HFD	0.218571(-0.019783 to 0.456926)	3.557633	P = 0.0808
	m_HFD vs. m_HFD+ mpd C	-0.155575(-0.401223 to 0.090073)	2.426655	P = 0.3323	f_CTR vs. f_Cmpd C	-0.141381(-0.366104 to 0.083342)	2.440815	P = 0.3311
	m_CTR vs. m_Cmpd C	-0.138675(-0.384323 to 0.106973)	2.163049	P = 0.4323	f_Cmpd C vs. f_HFD+Cmpd C	0.111571(-0.126783 to 0.349926)	1.81602	P = 0.5808
	m_CTR vs. m_HFD+ mpd C	-0.093075(-0.338723 to 0.152573)	1.451782	P = 0.7353	f_HFD vs. f_HFD+Cmpd C	-0.107(-0.345355 to 0.131355)	1.741612	P = 0.6131
	m_CTR vs. m_HFD	0.0625(-0.196436 to 0.321436)	0.924846	P = 0.9134	f_CTR vs. f_HFD	0.07719(-0.147532 to 0.301913)	1.332624	P = 0.7825
	m_Cmpd C vs. m_HFD+ mpd C	0.0456(-0.185999 to 0.277199)	0.754413	P = 0.9503	f_CTR vs. f_HFD+Cmpd C	-0.02981(-0.254532 to 0.194913)	0.514635	P = 0.9831
a. femoralis - SNP	m_Cmpd C vs. m_HFD	0.460025(0.039004 to 0.881046)	4.186587	P = 0.0279	f_CTR vs. f_HFD	0.340302(-0.067335 to 0.747938)	3.238789	P = 0.1264
	m_HFD vs. m_HFD+ mpd C	-0.333325(-0.754346 to 0.087696)	3.033518	P = 0.1608	f_HFD vs. f_HFD+Cmpd C	-0.313571(-0.745935 to 0.118792)	2.813707	P = 0.2177

	m_CTR vs. m_HFD	0.295125(-0.14867 to 0.73892)	2.548038	P = 0.2912	f_Cmpd C vs. f_HFD	0.271429(-0.160935 to 0.703792)	2.435555	P = 0.333
	m_CTR vs. m_Cmpd C	-0.1649(-0.585921 to 0.256121)	1.500719	P = 0.7151	f_CTR vs. f_Cmpd C	0.068873(-0.338763 to 0.476509)	0.655493	P = 0.9663
	m_Cmpd C vs. m_HFD+ mpd C	0.1267(-0.270242 to 0.523642)	1.223014	P = 0.8228	f_Cmpd C vs. f_HFD+Cmpd C	-0.042143(-0.474506 to 0.390221)	0.378152	P = 0.9931
	m_CTR vs. m_HFD+ mpd C	-0.0382(-0.459221 to 0.382821)	0.34765	P = 0.9947	f_CTR vs. f_HFD+Cmpd C	0.02673(-0.380906 to 0.434366)	0.254402	P = 0.9979
a. mesenterica	m_CTR vs. m_HFD+ mpd C	-0.543(-0.857225 to -0.228775)	6.645084	P = 0.0003	f_Cmpd C vs. f_HFD	-0.247(-0.555842 to 0.061842)	3.111067	P = 0.1509
	m_Cmpd C vs. m_HFD+ mpd C	-0.434(-0.736977 to -0.131023)	5.508354	P = 0.0027	f_CTR vs. f_Cmpd C	0.189167(-0.08707 to 0.465404)	2.663864	P = 0.2604
	m_HFD vs. m_HFD+ mpd C	-0.3604(-0.646888 to -0.073912)	4.837484	P = 0.0093	f_Cmpd C vs. f_HFD+Cmpd C	-0.177024(-0.474632 to 0.120584)	2.313857	P = 0.3775
	m_CTR vs. m_HFD	-0.1826(-0.489875 to 0.124675)	2.285155	P = 0.3853	f_HFD vs. f_HFD+Cmpd C	0.069976(-0.227632 to 0.367584)	0.91465	P = 0.9157
	m_CTR vs. m_Cmpd C	-0.109(-0.431703 to 0.213703)	1.298869	P = 0.7952	f_CTR vs. f_HFD	-0.057833(-0.33407 to 0.218404)	0.814415	P = 0.9384
	m_Cmpd C vs. m_HFD	-0.0736(-0.369362 to 0.222162)	0.956923	P = 0.9051	f_CTR vs. f_HFD+Cmpd C	0.012143(-0.251474 to 0.27576)	0.179183	P = 0.9993
a. mesenterica + L-NAME	m_HFD vs. m_HFD+ mpd C	0.303067(-0.013573 to 0.619707)	3.680562	P = 0.0646	f_CTR vs. f_HFD	-0.177933(-0.659897 to 0.304031)	1.436124	P = 0.742
	m_Cmpd C vs. m_HFD+ mpd C	0.281542(-0.053322 to 0.616406)	3.233077	P = 0.124	f_Cmpd C vs. f_HFD	-0.188(-0.726852 to 0.350852)	1.35718	P = 0.7731
	m_CTR vs. m_HFD+ mpd C	0.153952(-0.193344 to 0.501248)	1.704623	P = 0.6283	f_CTR vs. f_HFD+Cmpd C	-0.122814(-0.582759 to 0.33713)	1.038706	P = 0.8823
	m_CTR vs. m_HFD	-0.149114(-0.488728 to 0.1905)	1.84	P = 0.6353	f_Cmpd C vs. f_HFD+Cmpd C	-0.132881(-0.652132 to 0.38637)	0.995485	P = 0.8946
	m_CTR vs. m_Cmpd C	-0.127589(-0.484255 to 0.229077)	1.375606	P = 0.7658	f_HFD vs. f_HFD+Cmpd C	0.055119(-0.464132 to 0.57437)	0.412927	P = 0.9911

	m_Cmpd C vs. m_HFD	-0.021525(-0.348415 to 0.305365)	0.253212	P = 0.9979	f_CTR vs. f_Cmpd C	0.010067(-0.471897 to 0.492031)	0.081249	P > 0.9999
a. mesenterica - SNP	m_Cmpd C vs. m_HFD	0.19725(-0.439041 to 0.833541)	1.192074	P = 0.8336	f_Cmpd C vs. f_HFD+Cmpd C	0.206762(-0.806259 to 1.219782)	0.793965	P = 0.9425
	m_CTR vs. m_HFD	0.178643(-0.482415 to 0.839701)	1.039172	P = 0.8823	f_HFD vs. f_HFD+Cmpd C	0.176429(-0.836592 to 1.189449)	0.677485	P = 0.963
	m_Cmpd C vs. m_HFD+ mpd C	0.150083(-0.501729 to 0.801896)	0.885425	P = 0.9229	f_CTR vs. f_Cmpd C	-0.147933(-1.08821 to 0.792343)	0.612012	P = 0.9723
	m_CTR vs. m_HFD+ mpd C	0.131476(-0.544535 to 0.807488)	0.747885	P = 0.9514	f_CTR vs. f_HFD	-0.1176(-1.057876 to 0.822676)	0.48652	P = 0.9857
	m_HFD vs. m_HFD+ mpd C	-0.047167(-0.663506 to 0.569173)	0.294277	P = 0.9967	f_CTR vs. f_HFD+Cmpd C	0.058829(-0.83849 to 0.956147)	0.25503	P = 0.9979
	m_CTR vs. m_Cmpd C	-0.018607(-0.712857 to 0.675643)	0.103064	P = 0.9999	f_Cmpd C vs. f_HFD	0.030333(-1.020928 to 1.081594)	0.112243	P = 0.9998
aorta	m_HFD vs. m_HFD+ mpd C	0.43455(-0.360244 to 1.229344)	2.091507	P = 0.4613	f_Cmpd C vs. f_HFD	0.54975(-0.326536 to 1.426036)	2.440442	P = 0.3321
	m_CTR vs. m_HFD	-0.347027(-1.079137 to 0.385083)	1.813264	P = 0.5804	f_Cmpd C vs. f_HFD+Cmpd C	0.537583(-0.408914 to 1.484081)	2.209406	P = 0.4174
	m_Cmpd C vs. m_HFD	-0.239675(-1.034469 to 0.555119)	1.153565	P = 0.8466	f_CTR vs. f_Cmpd C	-0.43675(-1.343793 to 0.470293)	1.873072	P = 0.5566
	m_Cmpd C vs. m_HFD+ mpd C	0.194875(-0.642911 to 1.032661)	0.889809	P = 0.9219	f_CTR vs. f_HFD	0.113(-0.794043 to 1.020043)	0.484619	P = 0.9858
	m_CTR vs. m_Cmpd C	-0.107352(-0.885924 to 0.671219)	0.527456	P = 0.982	f_CTR vs. f_HFD+Cmpd C	0.100833(-0.874208 to 1.075875)	0.402282	P = 0.9918
	m_CTR vs. m_HFD+ mpd C	0.087523(-0.691049 to 0.866094)	0.430027	P = 0.9901	f_HFD vs. f_HFD+Cmpd C	-0.012167(-0.958664 to 0.934331)	0.050004	P > 0.9999
